# A unique three-enzyme cascade mediates efficient regioselective and stereospecific epoxytetrahydrofuran ring formation in deoxyverrucosidin biosynthesis†

**DOI:** 10.1039/d5sc03423j

**Published:** 2025-07-21

**Authors:** Hui-Ling Wei, Xiao-Ling Chen, Yu Dai, Li Yang, Shu-Ming Li

**Affiliations:** a Philipps-Universität Marburg, Fachbereich Pharmazie, Institut für Pharmazeutische Biologie und Biotechnologie Robert-Koch-Straße 4 35037 Marburg Germany shuming.li@staff.uni-marburg.de; b Haikou Key Laboratory for Research and Utilization of Tropical Natural Products, Institute of Tropical Bioscience and Biotechnology, Chinese Academy of Tropical Agricultural Sciences 571101 Haikou P. R. China

## Abstract

The fungal octaketide deoxyverrucosidin shares the same α-pyrone core with several nonaketides, including aurovertins, citreoviridin, and asteltoxin. Deoxyverrucosidin features a unique epoxytetrahydrofuran ring. In this study, we demonstrate that this ring system is formed *via* a flavin-containing monooxygenase-mediated epoxidation on the polyene chain, followed by rearrangement with an epoxide expandase and a second epoxidation on the resulting 2,5-dihydrofuran ring with a cytochrome P450 enzyme. This catalytic cascade differs clearly from the formation of dioxybicyclooctane or tetrahydrofuran motifs in other α-pyrone-containing metabolites, involving one flavin-containing monooxygenase and one hydrolase for up to two rounds of epoxide ring formation and expansion.

## Abstract 2

A catalytic cascade consisting of a flavin-containing monooxygenase, an epoxide expandase, and a cytochrome P450 enzyme is responsible for the construction of an epoxytetrahydrofuran ring system.

## Graphical abstract

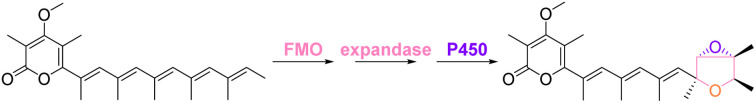

## Introduction

The remarkable structural diversity and complexity of fungal metabolites are results of cooperation of the backbone and tailoring enzymes in their biosynthetic pathways.^1^ Following the formation of core skeletons, tailoring enzymes play a vital role in modification of these fundamental building blocks into mature natural products.^2^ Converting linear frameworks to cyclic scaffolds can lead to morphed scaffolds, enhanced structural rigidity, and improved biological activities.^3^ Thus, elucidating the multicomponent biosynthetic processes would facilitate understanding nature's machinery logic, identifying novel enzymes, and optimally applying the potential of secondary metabolites as a source of novel chemicals.

**Fig. 1:**
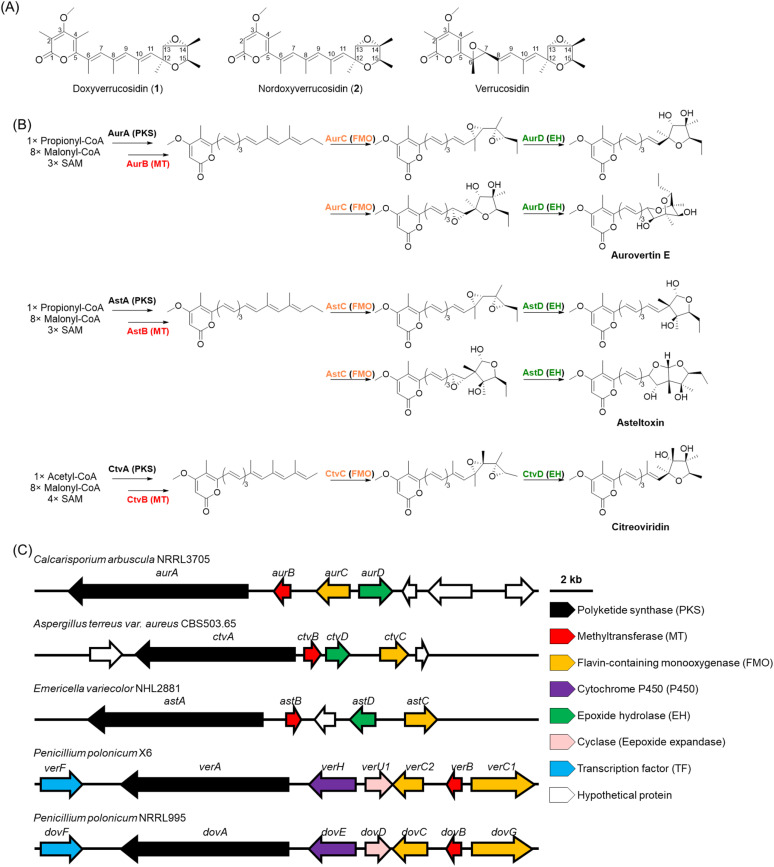
(A) Structures of deoxyverrucosidin (1), nordeoxyverrucosidin (2), and verrucosidin; (B) reported biosynthetic pathways of aurovertins, citreoviridin and asteltoxin; (C) biosynthetic gene clusters of deoxyverrucosidin and verrucosidin from *Penicillium polonicum*, aurovertins from *Calcarisporium arbuscula*, citreoviridin from *Aspergillus terreus*, and asteltoxin from *Emericella variecolor*.

Deoxyverrucosidin (1) and nordeoxyverrucosidin (2) (Fig. 1A) produced by *Penicillium* species belong to α-pyrone-containing polyketides.^4–7^ The members of this class are often characterized by a modified polyene linker such as a 3,6-dioxabicyclo-[3.1.0]-hexane (epoxytetrahydrofuran) ring in 1 and 2, a tetrahydrofuran (THF) ring in citreoviridin,^8^ or a dioxybicyclooctane (DBO) moiety in aurovertin^9^ and asteltoxin^10^ (Fig. 1B). These compounds exhibit potent inhibitory activity against mitochondrial oxidative phosphorylation by binding to the β subunit of F1-ATPase and have been implicated as potential therapeutics against cancer.^11^ The biosynthesis of aurovertin, citreoviridin, and asteltoxin has been reported to follow the same logic (Fig. 1B).^8–10^ Polyketide synthases (PKSs, AurA, CtvA, and AstA) are responsible for constructing the α-pyrone-polyene backbone. Subsequently, methyltransferases (MTs, AurB, CtvB, and AstB) catalyze O-methylation of the hydroxyl group at the α-pyrone ring to generate the necessary precursors for bisepoxidations mediated by flavin-containing monooxygenases (FMOs, AurC, CtvC, and AstC). Then, epoxide hydrolases (EHs, AurD, CtvD, and AstD) catalyze the conversion of the bisepoxides to tetrahydrofuran rings.^8–10^ In the biosynthesis of aurovertin E and asteltoxin, one additional epoxide is formed and hydrolyzed by the same FMOs and EHs as those in the first round, leading to the formation of a 2,6-dioxabicyclo-[3.2.1]-octane ring in aurovertin E^9^ and a 2,8-dioxabicyclo-[3.3.0]-octane ring in asteltoxin,^10^ respectively.

It has been reported that verrucosidin (Fig. 1A), a derivative of 1 with an additional epoxide ring on the polyene chain, exhibited potent antitumor activity.^12^ However, the biosynthesis of verrucosidin remains to be elucidated, although its biosynthetic gene cluster in *Penicillium polonicum* X6 was confirmed by targeted disruption of the PKS gene *verA*.^13^ The tailoring enzymes required for the epoxytetrahydrofuran formation and reaction order were unknown prior to this study. No homologue of the epoxide hydrolases AurD, CtvD, and AstD from the reported pathways was found in the reported *ver* cluster (Fig. 1C). Therefore, we expected intriguing biosynthetic logic and decided to investigate the biosynthesis of verrucosidin and its congeners.

**Fig. 2:**
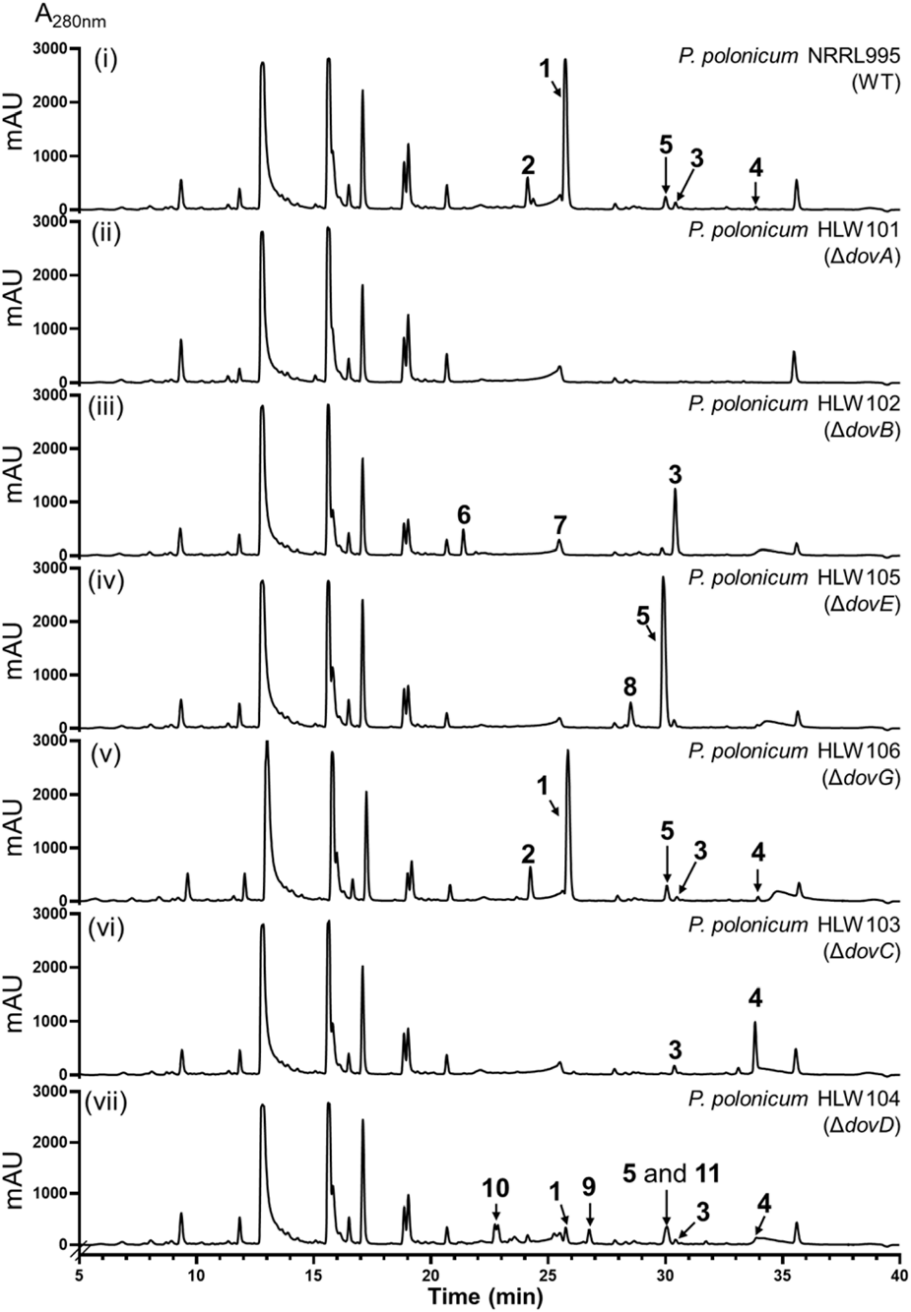
HPLC profiles of the metabolites from *P. polonicum* strains (i–vii). The chromatograms were monitored at 280 nm.

Mining the genome of *Penicillium polonicum* NRRL995 using the polyketide synthase gene *verA* from the identified verrucosidin cluster revealed the presence of one candidate cluster on the contig JAPDKX010000002.1 (GenBank) with a high sequence identity of 99% on the nucleotide level (Tables S1 and S2†). Both clusters contain seven putative genes with very high identity and the same orientation. *i.e.*, genes coding for one PKS, one MT, two FMOs, one P450 enzyme, one putative cyclase, and one transcription factor (Tables S1 and S3†). Isolation and structural elucidation of secondary metabolites indicated deoxyverrucosidin (1) to be the final pathway product in *Penicillium polonicum* NRRL995 (Fig. 2i). The cluster is therefore termed *dov* hereafter. In this study, we confirm the responsibility of the *dov* cluster for the biosynthesis of 1 and elucidate the reaction steps by gene deletion and heterologous expression experiments (Tables S4–S6 and Fig. S1 and S2†). We demonstrated that the epoxytetrahydrofuran ring is formed through a unique three-enzyme cascade, which differs clearly from that of other reported fungal mycotoxins.

## Results and discussion

### Analysis of the putative *dov* biosynthetic gene cluster

A BLASTn search using the DNA sequence of the *dov* cluster from *P. polonicum* NRRL995 provided evidence for the widespread distribution of orthologous clusters. The *dov* cluster was identified in seven of the ten sequenced *P. polonicum* strains with a sequence identity of 99% on the nucleotide level (Table S2†). A homologous *dov* cluster sharing a sequence identity of 97% was also found in *Penicillium* sp. CMV-2018d. *Penicillium glandicola* 3C contains a *dov-*like cluster with a lower sequence identity of 85% without a transcription factor (Table S2†). A BLASTp search using the seven Dov proteins listed in Table S1† as queries revealed nearly identical proteins between *P. polonicum* NRRL995 and *P. polonicum* X6, with a mere difference in the polypeptide length of the putative flavin-containing monooxygenases DovC and VerC2. Three enzymes from the *dov* cluster, *i.e*., DovA (PKS), DovB (MT), and DovC (FMO), share meaningful sequence identities of 38–50% on the amino acid level with their counterparts AurA/CtvA/AstA, AurB/CtvB/AstB, and AurC/CtvC/AstC mentioned above (Table S3† and Fig. 1). Like its homologues AurA/CtvA/AstA, DovA also shares the same domain architecture KS-AT-DH-cMT-KR-ACP. Notably, the *dov* and *ver* clusters are distinguished from *aur*, *ctv*, and *ast* clusters by the absence of an epoxide hydrolase, but the presence of three other enzymes, *i.e.,* a putative cyclase, a P450 enzyme, and an additional FMO. These unique tailoring enzymes may catalyze distinct reaction steps from the other known pathways listed in Fig. 1B.

### The newly identified *dov* cluster is responsible for the production of deoxyverrucosidin

To prove the pathway product encoded by the *dov* cluster, the *P. polonicum* NRRL995 wild-type (WT) strain was cultivated in a rice medium for 14 days. HPLC analysis revealed the presence of one dominant peak 1, accompanied by 2 and other minor peaks (3–5) (Fig. 2i). Compounds 1 and 2 were isolated and identified as deoxyverrucosidin and nordeoxyverrucosidin, respectively (Table S7 and Fig. S4–S6†), which differ from each other by one methyl group at the α-pyrone ring. These mycotoxins had been identified in two *Penicillium* strains^6,7^ (see the ESI† for fungal cultivation, metabolite isolation, and structure elucidation by NMR, MS, and ECD analyses).

To identify the relationship of the *dov* cluster with 1 and 2, we first knocked out *dovA* in *P. polonicum* by the split-marker strategy^14^ (Fig. S1A, see the ESI for genetic protocols†), which completely abolished the production of not only the major products 1 and 2, but also the minor peaks 3–5 observed in the WT strain (Fig. 2i and ii), proving unequivocally the responsibility of the *dov* cluster for the formation of 1–5.

**Fig. 3:**
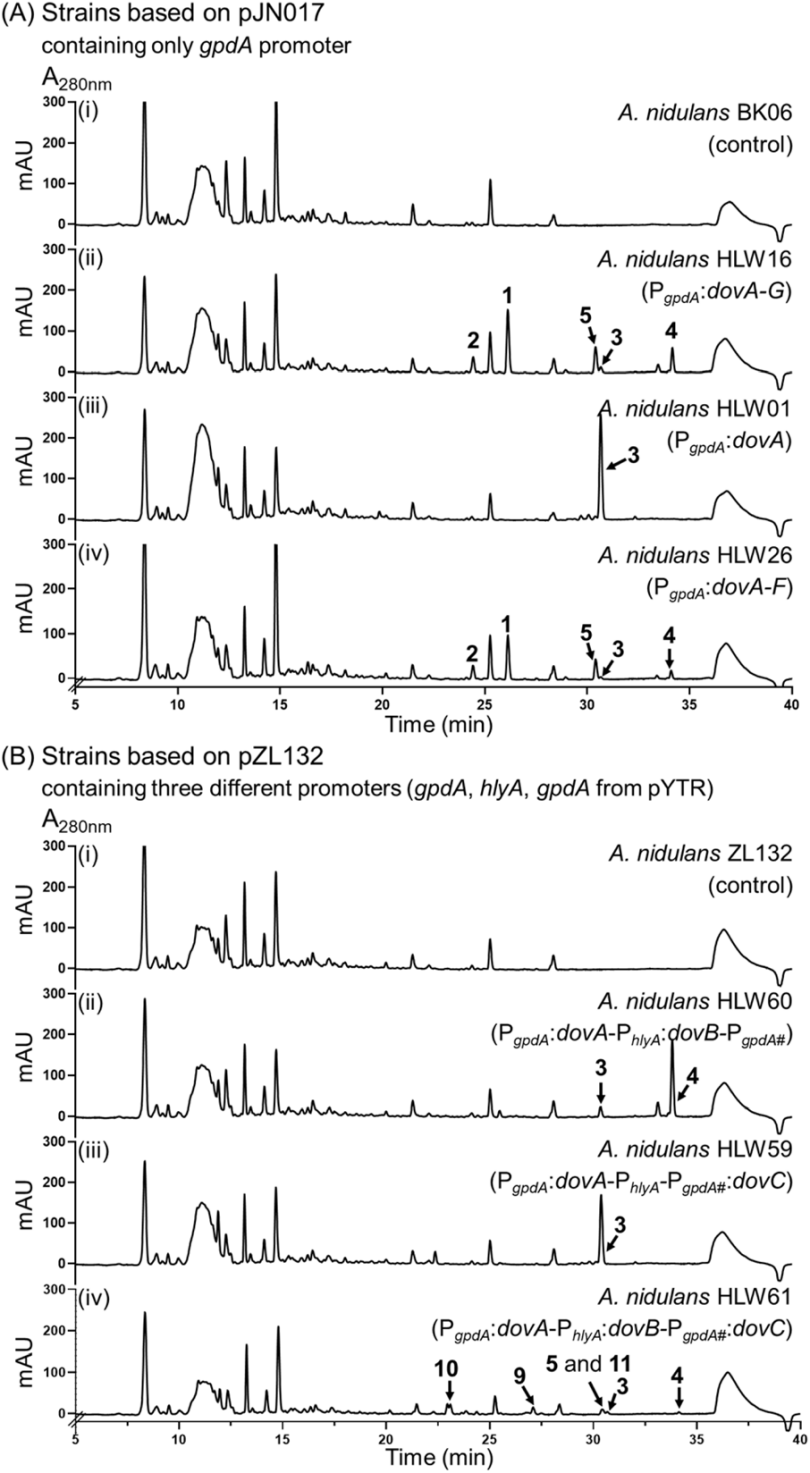
HPLC profiles of the metabolites from *A. nidulans* transformants (Ai–Aiv and Bi–Biv). The chromatograms were monitored at 280 nm.

To prove how many genes are necessary for the biosynthesis of 1 and 2, we introduced the seven genes *dovABCDEFG* into the genome of *A. nidulans* LO8030 (Fig. S2†) and cultivated the transformant *A. nidulans* HLW16 in a rice medium for 7 days for metabolite production. As shown in Fig. 3Aii, compounds 1–5 were clearly detected by HPLC analysis. In contrast, these compounds were absent in the control strain *A. nidulans* BK06 with the empty vector pJN017 (Fig. 3Ai). In comparison to the WT strain, compounds 4 and 5 are more accumulated in the transformant HLW16.

### The polyketide synthase DovA catalyzes chain elongation and methylation at each acetate unit

Having associated the *dov* cluster with the production of compounds 1, we proceeded to characterize the polyketide backbone catalyzed by DovA. Overexpression of DovA in *A. nidulans* HLW01 (Fig. S2†) resulted in the accumulation of a predominant peak sharing the same retention time, UV spectra, and MS data with compound 3 in the WT and HLW16 (Fig. 3Aiii). Isolation and structure elucidation proved 3 to be an α-pyrone derivative with highly methylated and conjugated polyene chain (Table S8, Fig. 5 and S7–S11†). This means that DovA assembles not only the polyketide chain but also its methylation, being in agreement with its homologues AurA/CtvA/AstA. However, AurA/CtvA/AstA add only three or four methyl groups to the nonaketides, while DovA methylates all eight acetate units in the octaketide 3. Identification of 3 in the transformant HLW01 also proved that DovA utilizes acetyl-CoA as the starter unit as in the case of CtvA, but differs from AurA and AstA, which accept propyl-CoA as the starter unit (Fig. 1B). Feeding with ^13^C-labelled precursors, ^13^CH_3_–COONa or [methyl-^13^C]-l-methionine, into the WT proved the incorporation of eight acetate units into 1 and 2. LC-MS analysis also showed that six and seven labeled methyl groups were incorporated into 1 and 2, respectively (Fig. S3†).

### Methylation catalyzed by DovB is not essential, but beneficial for deoxyverrucosidin biosynthesis

Studies on the biosynthetic pathways for aurovertins, citreoviridin, and asteltoxin have demonstrated that the FMO-catalyzed epoxidation at the distal polyene olefins requires *O*-methylation of α-pyrone. This methylation enhances the substrate's hydrophobicity and significantly improves the selectivity of FMOs for epoxidation at certain double bonds.^15^ However, recent research on aspernidgulene biosynthesis from *A. nidulans* indicated that an *O*-methylation is not necessary for the subsequent FMO-catalyzed reaction, despite the aspernidgulene PKS product also possessing a comparable polyene tail.^16^ The divergences in the relationship between *O*-methylation and epoxidation processes prompted us to investigate the importance of methylation on the hydroxyl group at C-3 of 3 for further metabolism.

We first proved the function of DovB as an *O*-methyltransferase for 3 by cloning *dovA* and *dovB* together into the vector pZL132 ^17^ and subsequent detection of their expression in *A. nidulans* HLW60. As shown in Fig. 3Bii, accumulation of 3 was drastically reduced and a peak 4 was detected as the predominant product, which was identified as the *O*-methylated derivative of 3 (Table S9 and Fig. S12–S16†). This unequivocally proved the DovB function in the deoxyverrucosidin biosynthesis. Afterward, we deleted *dovB* in *P. polonicum*. The resulting mutant HLW102 does not produce compounds 1 and 2 anymore. Instead, 3 was accumulated as a major product, accompanied by two additional peaks 6 and 7 (Fig. 2iii and S1B†). Isolation and structure elucidation confirmed 3 to be the same product after *dovA* expression in *A. nidulans* (Fig. 3Aiii). Compound 7 was proven to be a derivative of 1 without the *O*-methylation of the hydroxyl group at C-3 of the α-pyrone ring. In compound 6, the epoxytetrahydrofuran ring in 7 is replaced by a 2,5-dihydrofuran ring (Tables S11, S12 and Fig. S24–S35†) and could be considered a precursor of 7. This proved, differing from those listed in Fig. 1B, that the DovA product can be successfully metabolized by other enzymes in the pathway, although with slightly reduced efficiency. However, 3 was still the predominant product in *A. nidulans* HLW59 after co-expression of *dovA* and *dovC* (Fig. 3Biii and S2†), indicating that metabolism of 3 by DovC needs the presence of additional enzymes.

### The epoxytetrahydrofuran ring is formed *via* P450-catalyzed epoxidation

**Fig. 4:**
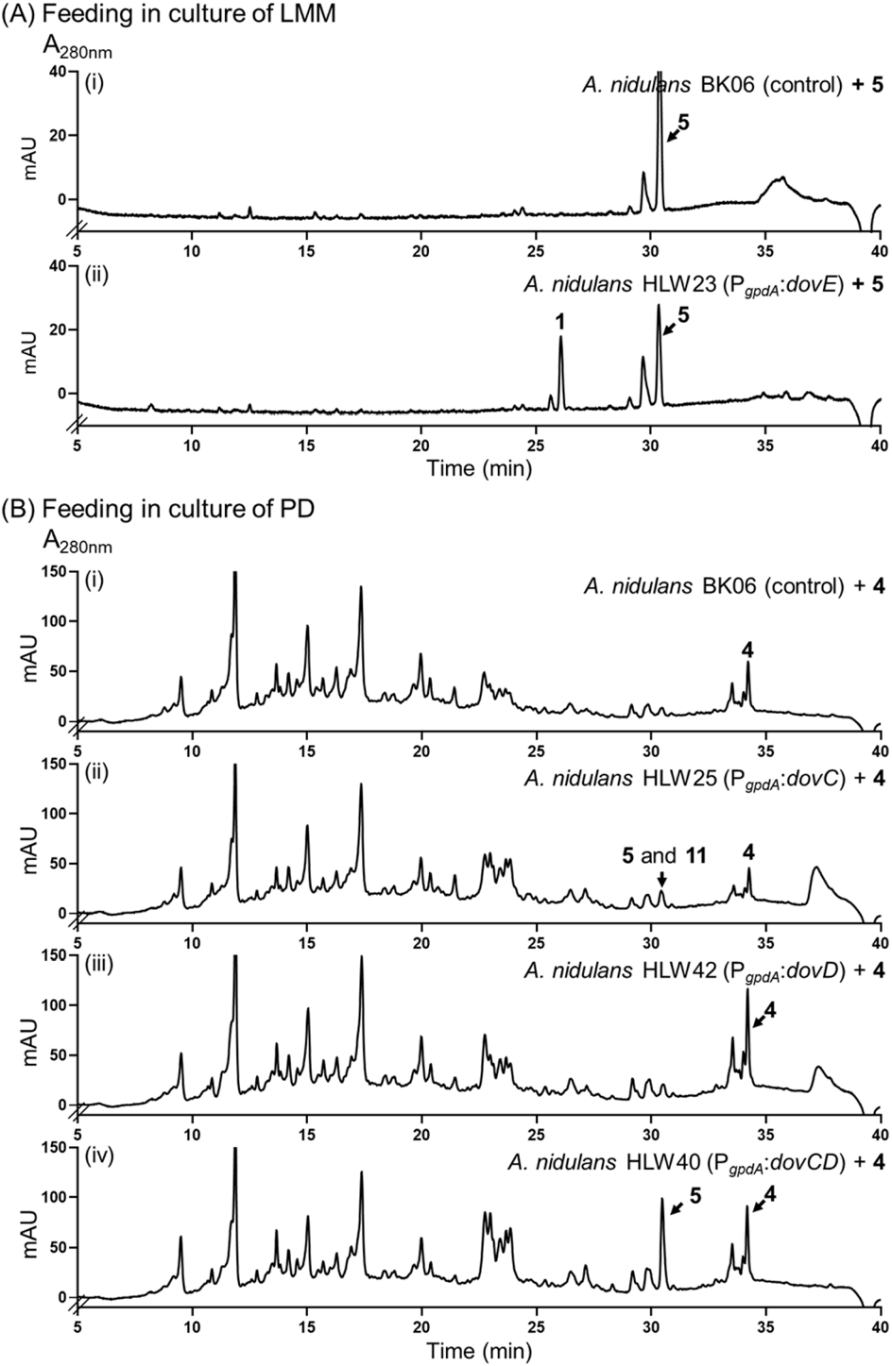
(A) HPLC analysis of *A. nidulans* cultures after feeding with 5 (i and ii); (B) HPLC analysis of *A. nidulans* cultures after feeding with 4 (i–iv).

The presence of a gene for P450 enzyme in the *dov* cluster has driven our interest in exploring its role in deoxyverrucosidin biosynthesis. Therefore, we knocked out *dovE* in the native producer, which led to the complete abolishment of 1 and 2 in the obtained strain HLW105 (Fig. S1C†) as confirmed by LC-MS analysis. Instead, 5 was accumulated as a predominant peak, along with a new minor peak 8 (Fig. 2iv). Isolation and structure elucidation identified 5 as an analogue of 1, by replacement of the epoxytetrahydrofuran ring with a 2,5-dihydrofuran ring (Table S10 and Fig. S17–S23†). Compound 8 was identified as a 5 congener without the methyl group at C-2 of the α-pyrone ring (Table S13 and Fig. S36†). Comparing their structures, it can be expected that 1 and 2 are DovE products of 5 and 8, respectively. To prove this hypothesis, we constructed a *dovE*-expression strain *A. nidulans* HLW23 (Fig. S2†) and cultivated it in the presence of 5. HPLC analysis confirmed the successful conversion of 5 to 1, with a product yield of 40% after cultivation of 0.1 mM substrate for 7 days. In comparison, no product was detected in the control strain (Fig. 4A). These findings confirm 5 as the direct biosynthetic precursor of 1 and imply that DovE serves as an epoxidase with high regio selectivity for the double bond of the 2,5-dihydrofuran ring. Moreover, identification of DovE for the last step in the biosynthesis of 1 also suggested that the conversion of 4 to 5 should be catalyzed by the remaining three enzymes DovC, DovD, and DovG or their combinations.

**Fig. 5:**
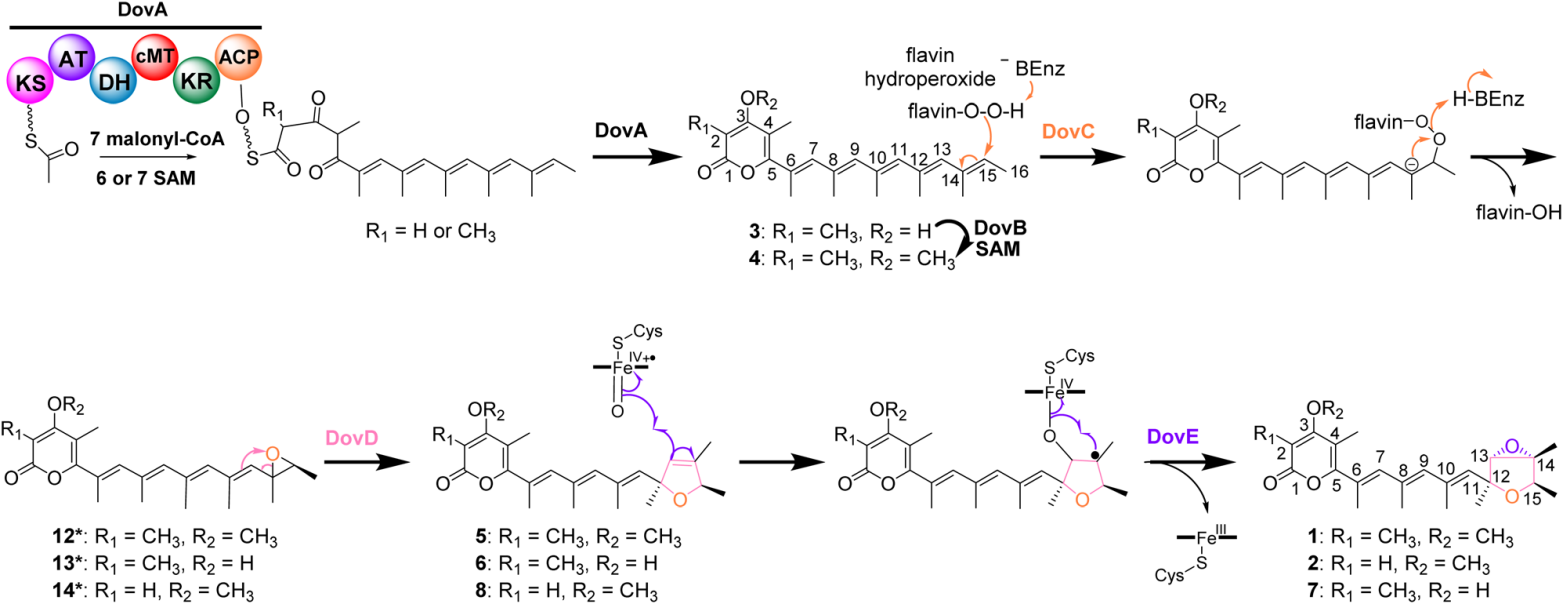
Proposed biosynthetic pathway of deoxyverrucosidin (1) and congeners.

### DovC and DovD are together responsible for the formation of the 2,5-dihydrofuran ring

Unlike those of aurovertins, citreoviridin, and asteltoxin, the *ver* and *dov* clusters from *P. polonicum* contain two putative flavin-dependent monooxygenases, which were proposed to be involved in the epoxidation steps.^4^ To evaluate their roles in the biosynthesis of 1, we individually deleted *dovC* and *dovG*. Surprisingly, the Δ*dovG* strain HLW106 exhibited almost the same metabolic profile as the WT strain (Fig. 2v and S1D†), while deletion of *dovC* in the strain HLW103 (Fig. S1E†) completely abolished the production of 1, 2, and 5, with accumulation of 3 as a minor and 4 as a major product (Fig. 2vi). Furthermore, in the *dovABCDEF* expression strain *A. nidulans* HLW26 (Fig. S2†), the production of 1–5 can also be detected (Fig. 3Aiv). Therefore, DovG can be excluded from the biosynthesis of 1, while DovC is involved in the conversion of 4. Based on the homology of DovC with other epoxidases such as AurC/CtvC/AstC, it can be speculated that DovC also catalyzes epoxidation on the polyene tail.

In the biosynthesis of structurally related compounds (Fig. 1B), the conversion of the bisepoxide to the dihydroxylated tetrahydrofuran ring is catalyzed by a membrane-bound epoxide hydrolase. As mentioned above, such a hydrolase is absent in the *dov* cluster. However, DovD is predicted to be a transmembrane cyclase. BLASTP search revealed the presence of nine orthologues in *P*. *polonicum* and other *Penicillium* species (Table S2†) with an identity of more than 80% on the amino acid level. Other remaining hits showed only a sequence identity of lower than 41% without known functions. These findings suggest that DovD is a rare and previously uncharacterized enzyme likely restricted to Dov pathways. Deletion of *dovD* in *P. polonicum* led to the accumulation of three new peaks, 9, 10, and 11 (Fig. 2vii and S1F†), along with a small amount of 1 and 5. Moreover, co-expression of *dovABC* in *A. nidulans* (HLW61, Fig. S2†) also resulted in a similar secondary metabolite profile, with the exception of the absence of 1 (Fig. 3Biv). The structure of 9 was elucidated to be C-15 monohydroxylated and 10 to be C-14 and C-15 dihydroxylated derivatives of 4 (Tables S14, S15 and Fig. S37–S46†), which can be considered as products after a reductive and hydrolytic epoxide ring opening, respectively. Compound 11 was isolated as a mixture with 5, also sharing the same molecular formula and UV absorption. Attempts to separate them from each other by normal-phase chromatography, reverse-phase chromatography or with a chiral column failed (data not shown). Interpretation and comparison of NMR data confirmed their diastereomeric feature (Table S16 and Fig. S47–S52†). The configurations of the 2,5-dihydrofuran ring moieties in 5 (12*S*,15*R*) and 11 (12*R*,15*R*) were determined by analysis of CD and NOESY spectra. It should be mentioned that 5 has the same configuration as 1 (also isolated from Δ*dovD*-mutant HLW104) at this position, indicating that 11 cannot be or is very poorly accepted by the P450 DovE.

LC-MS analysis revealed low conversion of 4 to 5 and its stereoisomer 11 in the *dovC* overexpression strain *A. nidulans* HLW25, but not in the strain *A. nidulans* HLW42 with *dovD* expression (Fig. 4Bii, iii and S2†). It seems that DovC alone can convert 5, but with low efficiency and stereospecificity. Feeding 4 into the strain *A. nidulans* HLW40 with co-expression of *dovC* and *dovD* caused a significantly increased conversion to 5 (Fig. 4Biv and S2†). These results proved the necessary cooperation of DovC and DovD in the formation of the DovE substrate 5. Feeding 9 and 10 to HLW42 did not yield any detectable transformation products (data not shown), indicating that these compounds are not direct substrates of DovD, but rather shunt products.

Although DovD was predicted as a cyclase, our results provide clear evidence for its role as an epoxide expandase, catalyzing the regio- and stereo-selective conversion of a three-membered ring into a five-membered ring. It should also be mentioned that DovD plays a dual role in the identified biosynthetic pathway. In addition to the ring expansion, DovD also significantly contributes to directing the pathway flow and enhancing the efficiency of the product formation. The total product yields in both Δ*dovD* mutant *P. polonicum* HLW104 and *dovABC* overexpression strain *A. nidulans* HLW61 are significantly lower than those in strains with *dovD* (Fig. 2i, vii and 3Aii, iv, Biv).

### Postulated biosynthetic pathway for deoxyverrucosidin in *Penicillium polonicum*

Based on our results, we propose a biosynthetic pathway for deoxyverrucosidin in *Penicillium polonicum* NRRL995 (Fig. 5). The PKS DovA initiates the polyketide elongation by using acetyl-CoA as the starter unit, followed by chain extensions with 7 malonyl-CoA molecules as substrates, regioselective methylation, reduction, and intramolecular lactonization to form the carbon skeleton 3. Next, DovB installs a methyl group on the hydroxyl group of the α-pyrone ring in 3 to generate 4. As speculated above, DovC catalyzes the epoxidation on the ultimate double bond between C-14 and C-15 *via* a mechanism as proposed for other FMO-mediated double bond epoxidations (Fig. 5 and S58†),^18^ resulting in the formation of the postulated products 12*–14*. However, these compounds were detected neither in the Δ*dovD* mutant *P. polonicum* HLW104 nor in *A. nidulans* HLW61 with *dovABC* overexpression (Fig. 2vii and 3Biv). The reason could be that the lipophilic polyene tail of 4 and congeners is associated with the cell membrane for better metabolism by the membrane-bound enzymes DovD and DovE and therefore their products are almost not released into the medium. A similar phenomenon has been reported for the biosynthetic pathways listed in Fig. 1B. In those cases, the proposed epoxides are converted by the membrane-bound epoxide hydrolases.^8–10^ Despite multiple attempts, no soluble or active DovC could be obtained. Expression of *dovC* alone or together with *dovD* in *A. nidulans*, followed by *in vitro* assays of 4 with lysates of the transformants in the presence of NADPH and FAD, did not lead to product detection on LC-MS, by both UV and ion detection. Similar results were also obtained with lysates of *dovC* and *dovD* transformants in *Saccharomyces cerevisiae* (Fig. S59†). These results could suggest that DovC and/or DovD may require specific cofactors or/and membrane environments for their activities that are not fully recapitulated in the systems used. Nevertheless, the results of gene disruption and feeding experiments confirmed the critical role of DovC in the conversion of 4 to 5 with the help of the epoxide expandase DovD, catalyzing the rearrangement of the epoxide ring to form a 2,5-dihydrofuran ring. Finally, the P450 DovE installs regioselectively and stereospecifically the epoxide ring on the double bond of the 2,5-dihydrofuran ring *via* a mechanism suggested for the styrene epoxidation,^19^ to assemble the epoxytetrahydrofuran ring moiety of compound 1 as well as its demethylated analogue 2 (Fig. 5 and S60†).

Differing from the formation of tetrahydrofuran rings by hydrolysis with a hydrolase of bisepoxide rings in aurovertin, citreoviridin, and asteltoxin, the proposed epoxide in this pathway is converted to a dihydrofuran ring by an epoxide expandase. In the biosynthesis of aurovertin and asteltoxin, the construction of two different intermediates with an epoxide structure is catalyzed by the same enzyme, AurC or AstC. In our cases, the two epoxide derivatives, 12* and 1, are products of two different enzymes, DovC (FMO) and DovE (P450), respectively. The formation of the epoxytetrahydrofuran moiety in deoxyverrucosidin is unique among structurally related compounds shown in Fig. 1B. Differing from the hydrolytic products of bisepoxides of the mentioned pathways, the double bond of the 2,5-dihydrofuran ring in the products 5, 6, and 8 of DovABCD enables subsequent epoxidation to form the unique epoxytetrahydrofuran moiety.

As mentioned above, no epoxides were accumulated in the absence of the epoxide expandase DovD (Fig. 2vii and 3Biv). Instead, a number of minor peaks, including pathway or non-pathway products were detected. A hypothesis for the formation of 1 (approximately 7.6% of the WT) and shunt products 9, 10, and 11 without involvement of DovD is illustrated in Fig. S61.†

## Conclusions

In this study, we identified the biosynthetic gene cluster of deoxyverrucosidin in *P. polonicum* NRRL995 and elucidated its biosynthetic pathway through gene disruption and heterologous expression with or without substrate feeding. We demonstrated the coordinated action of a novel FMO-epoxide expandase-P450 enzymatic triad in regioselective and stereospecific construction of the epoxytetrahydrofuran moiety during deoxyverrucosidin biosynthesis. Collaboration of the FMO DovC and the epoxide expandase DovD is critical for the formation of derivatives with a 2,5-dihydrofuran ring.

## Author contributions

S. M. L., H. L. W., and X. L. C. conceived this project. All authors contributed to the design of the study methods and analysis of the results. H. L. W., X. L. C., and L. Y. generated the experimental data. The manuscript was written with contributions of all authors.

## Conflicts of interest

The authors declare no competing interests.

## Supplementary Material

SC-016-D5SC03423J-s001

## Data Availability

The supporting data are available within the article and its ESI file (general information, LC-MS data, NMR spectra, and UV spectra).†
