# Supplementary material for: A unique three-enzyme cascade mediates efficient regioselective and stereospecific epoxytetrahydrofuran ring formation in deoxyverrucosidin biosynthesis
Source: Chem Sci. 2025 Jul 21;16(34):15564–70. doi: 10.1039/d5sc03423j (PMC12312803; doi:10.1039/d5sc03423j)
Supplement: SC-016-D5SC03423J-s001 [file SC-016-D5SC03423J-s001.pdf]

Supplementary Information (SI) for Chemical Science.
This journal is © The Royal Society of Chemistry 2025

Supporting Information

A unique three-enzyme cascade mediates efficient regioselective and stereospecific epoxytetrahydrofuran ring formation in the deoxyverrucosidin biosynthesis

Hui-Ling Wei,[†a] Xiao-Ling Chen,[†a] Yu Dai,[a] Li Yang,[b] and Shu-Ming Li[*a]

[a] Philipps-Universität Marburg, Fachbereich Pharmazie

Institut für Pharmazeutische Biologie und Biotechnologie

Robert-Koch-Straße 4, 35037 Marburg, Germany

[b] Haikou Key Laboratory for Research and Utilization of Tropical Natural Products

Institute of Tropical Bioscience and Biotechnology

Chinese Academy of Tropical Agricultural Sciences, Haikou 571101, P. R. China

[†]These authors contributed equally

*Corresponding author. Email: shuming.li@staff.uni-marburg.de

Table of Contents

Experiment Procedures 6

1. Media, strains, and growth conditions 6

2. Genomic DNA isolation 6

3. PCR amplification, gene cloning, and plasmid construction 6

4. Genetic manipulation of *P. polonicum* 7

5. Heterologous expression in *A. nidulans* 8

6. Large-scale fermentation, extraction, and isolation of secondary metabolites 8

7. HPLC equipment for analysis and substance isolation 9

8. LC-HRMS analysis for secondary metabolites 9

9. NMR analysis 9

10. Structural elucidation 9

11. Feeding experiments 10

12. Preparation of DovC- and DovD-containing lysates from *A. nidulans* transformants 11

13. Expression and preparation of DovC- and DovD-containing lysates in *S. cerevisiae* 11

14. *In vitro* assays of 4 with DovC- and DovD-containing lysates from *A. nidulans* and *S. cerevisiae* 12

15. Measurement of optical rotations 12

16. The experimental and calculated electronic circular dichroism (ECD) spectra 12

17. Physiochemical properties of the compounds described in this study 12

Supplementary Tables 14

Table S1. Proteins encoded by the *dov* cluster in *Penicillium polonicum* NRRL995. 14

Table S2. Sequence identities of nucleotides of the *dov* cluster from *Penicillium polonicum* NRRL995 with those of sequenced *Penicillium polonicum* strains as well as other *Penicillium* species. 15

Table S3. Sequence identities of proteins encoded by the *dov* cluster from *Penicillium polonicum* NRRL995 with those of known clusters. 16

Table S4. Strains used in this study. 17

Table S5. Plasmids used and constructed in this study. 18

Table S6. Primers used in this study. 20

Table S7. NMR data of deoxyverrucosidin (**1**) and nordeoxyverrucosidin (**2**) in $CDCl_3$. 24

Table S8. NMR data of policosidin A (**3**) in DMSO-*d*$_6$. 25

Table S9. NMR data of policosidin B (**4**) in $CDCl_3$. 26

Table S10. NMR data of poloncosidin A (**5**) in DMSO-*d*$_6$ and $CDCl_3$. 27

Table S11. NMR data of policosidin C (**6**) in $CDCl_3$. 28

Table S12. NMR data of policosidin D (**7**) in DMSO-*d*$_6$. 29

Table S13. NMR data of poloncosidin B (**8**) in DMSO-*d*$_6$. 30

Table S14. NMR data of policosidin E (**9**) in $CDCl_3$. 31

Table S15. NMR data of a diastereomeric pair of policosidin F (**10**) in $CDCl_3$. 32

Table S16. NMR data of policosidin G (**11**) in $CDCl_3$. 33

Supplementary Figures........ 34

Figure S1. Target gene deletion and PCR verification for *P. polonicum* strains. 34

Figure S2. Heterologous expression and PCR verification for *A. nidulans* strains. 35

Figure S3. MS analysis of ^{13}C-labeled precursors supplied for *P. polonicum* NRRL 995. 36

Figure S4. ^1H NMR spectrum of deoxyverrucosidin (**1**) in $CDCl_3$ (500 MHz). 37

Figure S5. ^{13}C NMR spectrum of deoxyverrucosidin (**1**) in $CDCl_3$ (125 MHz). 38

Figure S6. ^1H NMR spectrum of nordeoxyverrucosidin (**2**) in $CDCl_3$ (500 MHz). 39

Figure S7. ^1H NMR spectrum of policosidin A (**3**) in DMSO-*d*$_6$ (500 MHz). 40

Figure S8. ^{13}C NMR spectrum of policosidin A (**3**) in DMSO-*d*$_6$ (125 MHz). 41

Figure S9. HSQC spectrum of policosidin A (**3**) in DMSO-*d*$_6$. 42

Figure S10. DQF-COSY spectrum of policosidin A (**3**) in DMSO-*d*$_6$. 43

Figure S11. HMBC spectrum of policosidin A (**3**) in DMSO-*d*$_6$. 44

Figure S12. ^1H NMR spectrum of policosidin B (**4**) in $CDCl_3$ (500 MHz). 45

Figure S13. ^{13}C NMR spectrum of policosidin B (**4**) in $CDCl_3$ (125 MHz). 46

Figure S14. HSQC spectrum of policosidin B (**4**) in $CDCl_3$. 47

Figure S15. DQF-COSY spectrum of policosidin B (**4**) in $CDCl_3$. 48

Figure S16. HMBC spectrum of policosidin B (**4**) in $CDCl_3$. 49

Figure S17. ^1H NMR spectrum of poloncosidin A (**5**) in DMSO-*d*$_6$ (500 MHz). 50

Figure S18. ^1H NMR spectrum of poloncosidin A (**5**) in $CDCl_3$ (500 MHz). 51

Figure S19. ^{13}C NMR spectrum of poloncosidin A (**5**) in $CDCl_3$ (125 MHz). 52

Figure S20. HSQC spectrum of poloncosidin A (**5**) in $CDCl_3$. 53
Figure S21. DQF-COSY spectrum of poloncosidin A (**5**) in $CDCl_3$. 54
Figure S22. HMBC spectrum of poloncosidin A (**5**) in $CDCl_3$. 55
Figure S23. NOESY spectrum of poloncosidin A (**5**) in $CDCl_3$. 56
Figure S24. ^{1}H NMR spectrum of policosidin C (**6**) in $CDCl_3$ (500 MHz). 57
Figure S25. ^{13}C NMR spectrum of policosidin C (**6**) in $CDCl_3$ (125 MHz). 58
Figure S26. HSQC spectrum of policosidin C (**6**) in $CDCl_3$. 59
Figure S27. DQF-COSY spectrum of policosidin C (**6**) in $CDCl_3$. 60
Figure S28. HMBC spectrum of policosidin C (**6**) in $CDCl_3$. 61
Figure S29. NOESY spectrum of policosidin C (**6**) in $CDCl_3$. 62
Figure S30. ^{1}H NMR spectrum of policosidin D (**7**) in DMSO-d_6 (500 MHz). 63
Figure S31. ^{13}C NMR spectrum of policosidin D (**7**) in DMSO-d_6 (125 MHz). 64
Figure S32. HSQC spectrum of policosidin D (**7**) in DMSO-d_6. 65
Figure S33. DQF-COSY spectrum of policosidin D (**7**) in DMSO-d_6. 66
Figure S34. HMBC spectrum of policosidin D (**7**) in DMSO-d_6. 67
Figure S35. NOESY spectrum of policosidin D (**7**) in DMSO-d_6. 68
Figure S36. ^{1}H NMR spectrum of poloncosidin B (**8**) in DMSO-d_6 (500 MHz). 69
Figure S37. ^{1}H NMR spectrum of policosidin E (**9**) in $CDCl_3$ (500 MHz). 70
Figure S38. ^{13}C NMR spectrum of policosidin E (**9**) in $CDCl_3$ (125 MHz). 71
Figure S39. HSQC spectrum of policosidin E (**9**) in $CDCl_3$. 72
Figure S40. DQF-COSY spectrum of policosidin E (**9**) in $CDCl_3$. 73
Figure S41. HMBC spectrum of policosidin E (**9**) in $CDCl_3$. 74
Figure S42. ^{1}H NMR spectrum of policosidin F (**10**) as a diastereomeric pair in $CDCl_3$ (500 MHz). 75
Figure S43. ^{13}C NMR spectrum of policosidin F (**10**) as a diastereomeric pair in $CDCl_3$ (125 MHz). 76
Figure S44. HSQC spectrum of policosidin F (**10**) as a diastereomeric pair in $CDCl_3$. 77
Figure S45. DQF-COSY spectrum of policosidin F (**10**) as a diastereomeric pair in $CDCl_3$. 78
Figure S46. HMBC spectrum of policosidin F (**10**) as a diastereomeric pair in $CDCl_3$**.** 79
Figure S47. ^{1}H NMR spectrum of a diastereomeric pair containing poloncosidin A (**5**) and policosidin G (**11**) in $CDCl_3$ (500 MHz). 80

Figure S48. ^{13}C NMR spectrum of a diastereomeric pair containing poloncosidin A (**5**) and policosidin G (**11**) in $CDCl_3$ (125 MHz). 81

Figure S49. HSQC spectrum of a diastereomeric pair containing poloncosidin A (**5**) and policosidin G (**11**) in $CDCl_3$. 82

Figure S50. DQF-COSY spectrum of a diastereomeric pair containing poloncosidin A (**5**) and policosidin G (**11**) in $CDCl_3$. 83

Figure S51. HMBC spectrum of a diastereomeric pair containing poloncosidin A (**5**) and policosidin G (**11**) in $CDCl_3$. 84

Figure S52. NOESY spectrum of a diastereomeric pair containing poloncosidin A (**5**) and policosidin G (**11**) in $CDCl_3$. 85

Figure S53. The experimental and calculated ECD spectra of **5** in MeOH. 86

Figure S54. The experimental and calculated ECD spectra of **9** in MeOH. 87

Figure S55. The experimental ECD spectra of compounds **1**, **2**, **5**–**8** in MeOH. 88

Figure S56. Mass spectra of compounds **1**–**11**. 89

Figure S57. UV spectra of compounds **1**–**11**. 90

Figure S58. Proposed mechanism of the DovC epoxidation. 91

Figure S59. LC-MS analysis of *in vitro* assays with DovC- and DovD-containing lysates. 92

Figure S60. Proposed mechanism of the DovE epoxidation. 93

Figure S61. Proposed biosynthetic pathway of shunt products in the absence of DovD. 94

Supplementary References 95

Experiment Procedures

1. Media, strains, and growth conditions

Strains used in this study are listed in Table S4. *Penicillium polonicum* NRRL 995 was grown at 25 °C on potato dextrose agar (PDA) plates for sporulation and cultivated on rice medium (20 g of rice with 30 mL of H_2O in a 250 mL flask) for secondary metabolite (SM) production. *P. polonicum* mutants obtained were grown on PDA supplemented with 300 µg/mL of hygromycin B under the same conditions.

Aspergillus nidulans strains were grown at 37 °C on glucose minimal medium (GMM) plates for sporulation, and on rice medium for SM production. Appropriate supplements, *i.e.* 1.2 g/L uridine, 1.0 g/L uracil, 0.5 mg/L pyridoxine, and 5.0 mg/L riboflavin were added as required.

Escherichia coli DH5α was cultivated in liquid lysogeny broth (LB) medium or on agar plates at 37 °C.[1] Ampicillin (100 µg/mL) was supplemented to cultivate recombinant *E. coli* strains.

Saccharomyces cerevisiae BJ5464-npgA and INVSc1 were used for cloning by homologous recombination and recombinant protein overproduction, respectively.[2] Cultivation was performed at 30 °C in yeast extract peptone dextrose (YPD) medium. Synthetic complete (SC) medium without uracil (SC-Ura) was used for selection.

2. Genomic DNA isolation

For genomic DNA isolation, *P. polonicum* and *A. nidulans* strains were cultured in PD medium with necessary nutritional additives at 25 °C or 37 °C for 2–3 days. The mycelia were collected and crushed with 3 glass beads in 400 µL LETS buffer (10 mM Tris-HCl, pH 8.0, 20 mM EDTA, 0.5% SDS, 0.1 M LiCl) in a MiniLys homogeniser (Bertin Technologies, Montigny-le-Bretonneux, France) for 4 min at full speed. After vigorous mixing for 4 min, 300 µL LETS buffer was added. The cell lysate was extracted with 700 µL phenol/chloroform/isoamyl alcohol (25:24:1). The gDNA was then precipitated by addition of double volume of absolute ethanol to the aqueous phase obtained from the previous step and washed with 1 mL 70% ethanol, dried, and dissolved in distillated H_2O.

3. PCR amplification, gene cloning, and plasmid construction

Plasmids generated and used in this study are listed in Table S5. The primers used for PCR amplification were synthesized by Seqlab GmbH (Göttingen, Germany) and are listed in Table S6. PCR amplification was carried out using the Phusion High-Fidelity DNA polymerase from New England Biolabs (NEB) on a T100 Thermal Cycler (Bio-Rad).

Plasmids for gene deletion and heterologous expression were constructed by homologous recombination in *E. coli* DH5α or *S. cerevisiae* BJ5464-npgA.[2,3]

For gene deletion experiments, two split marker strategy was used.[4] To construct the plasmids for gene deletion, an approximately 1.5 kbp fragment of the upstream of the target gene was amplified from the genomic DNA of *P. polonicum* and then ligated to the vector p5HY,[5] which was linearized by NotI and BglII, through homologous recombination in *E. coli* DH5α. In analogy, the downstream of the target gene was ligated to vector p3YG linearized by enzymes SalI and SlaI.[5]

The plasmids for heterologous expression in *A. nidulans* LO8030 were constructed by PCR amplification from genomic DNA of *P. polonicum* and subsequent cloning into SfoI-linearized vector pJN017 containing the flanking regions of the *wA* gene, the *gpdA* promoter, and the *afribo* selection marker, or vector pZL132 containing three different constitutive promoters linearized by SfoI or NotI.[6] The amplified genomic sequence was cloned between the *gpdA* promoter and the *afribo* marker using 25–30 bp overlaps to the SfoI restriction site.

4. Genetic manipulation of *P. polonicum*

Creation of mutated strains in *P. polonicum* was achieved through a homologous recombination strategy, performed using polyethylene glycol (PEG)–mediated protoplast transformation. For protoplastation, fresh spores of *P. polonicum* were inoculated in a 250 mL flask containing 50 mL YGG medium (8.0 g/L KCl, 16.0 g/L glucose, 6.6 g/L yeast nitrogen base, 1.5 g/L citric acid, 6.0 g/L KH_2PO_4, and 2.0 g/L yeast extract). The flask was shaken on a rotary shaker at 180 rpm and 28 °C for 12 h. The germlings were harvested by filtration and washed with an osmotic solution (1.2 M $MgSO_4$, 10 mM sodium phosphate buffer). The harvested mycelia were transferred to a sterile 100 mL flask. A lysing solution (20 mg yatalase from *Corynebacterium* sp. OZ-21 (OZEKI Co., Ltd.), 50 mg lysing enzyme from *Aspergillus*, and 200 μL of cellulase TXL dissolved in 10 mL of osmotic solution) was added to the flask. The mixture was kept at 30 °C, 100 rpm for 2–3 h. The obtained protoplasts were then collected using trapping buffer (0.6 M sorbitol, 0.1 M Tris–HCl, pH 7) and centrifuged at 5000 rpm and 4 °C for 15 min. The protoplast layer was separated by carefully transferring into a 15 mL Falcon tube and then washing twice with cold STC Buffer (1.2 M sorbitol, 10 mM $CaCl_2$, and 10 mM Tris–HCl, pH 7.5). The obtained protoplasts were resuspended in 200 μL cold STC buffer. For transformation, 100 μL of the protoplast suspension was incubated with 5–10 μg of purified DNA fragment on ice for 50 min. 1.25 mL of 60% PEG solution (60% PEG 4000, 50 mM $CaCl_2$, 50 mM Tris-HCl, pH 7.5) was added, gently swirled, and then incubated at room temperature for 20 min. 5 mL of STC buffer was then added to the mixture, followed by gentle mixing and spreading onto Petri dishes with SMM bottom medium (1.0% glucose, 50 mL/L salt solution, 1 mL/L trace element solution, 1.2 M sorbitol, and 1.6% agar). The plates were overlaid with SMM top medium (1.0% glucose, 50 mL/L salt solution, 1 mL/L trace element solution, 1.2 M sorbitol, and 0.8% agar) and incubated at 25 °C for 3–5 days. Both bottom and top agar media were supplemented with 300 μg/mL hygromycin B for selection. Hygromycin B-resistant fungal colonies were transferred onto fresh PDA

plates containing 300 μg/mL hygromycin B for further selection. Diagnostic PCR amplification was used to verify the resulted deletion mutants *P. polonicum* HLW101 (Δ*dovA*), HLW102 (Δ*dovB*), HLW103 (Δ*dovC*), HLW104 (Δ*dovD*), HLW105 (Δ*dovE*), and HLW106 (Δ*dovG*).

5. Heterologous expression in *A. nidulans*

A. nidulans LO8030 was used as the recipient host. Fungal protoplast preparation and transformation were performed according to the method described previously.[5] The expression plasmids pHLW01 (*dovA*), pHLW16 (*dovABCDEFG*), pHLW23 (*dovE*), pHLW25 (*dovC*), pHLW26 (*dovABCDEF*), pHLW40 (*dovCD*), pHLW42 (*dovD*), pHLW59 (*dovAC*), pHLW60 (*dovAB*), and pHLW61 (*dovABC*) were introduced into LO8030, resulting in the *A. nidulans* strains HLW01, HLW16, HLW23, HLW25, HLW26, HLW40, HLW42, HLW59, HLW60, and HLW61, respectively.

6. Large-scale fermentation, extraction, and isolation of secondary metabolites

For metabolites isolation, the fungal strains were cultivated in rice medium at 25 °C for 14 days. The fungal cultures were extracted twice with ethyl acetate (EtOAc) or petroether (PE) and concentrated under reduced pressure to obtain crude extracts.

To isolate compounds **1** and **2**, the EtOAc extract of *P. polonicum* NRRL 995 was fractionated by silica gel 60 (0.04–0.063 mm and 0.2–0.5 mm) column chromatography, eluted with a gradient of PE and EtOAc from 100:10 to 0:100 (v/v), yielding 21 fractions (1–21). Fraction 6 was purified via semi-preparative HPLC using an acetonitrile (ACN)/water (H_2O) (95:5, v/v) elution system, affording deoxyverrucosidin (**1**, 9.9 mg). Nordeoxyverrucosidin (**2**, 2.0 mg) was subsequently isolated from fraction 8 under modified chromatographic conditions with ACN/H_2O (70:30, v/v).

To isolate compounds **3**, **4**, **5**, and **8**, the PE extracts of mutant strains HLW102 (Δ*dovB*), HLW103 (Δ*dovC*), and HLW105 (Δ*dovE*) were obtained under light protection and first fractionated on a preparative HPLC with a linear gradient from 85 to 100% ACN in H_2O in 10 min. Further purification on a semi-preparative HPLC by using ACN/H_2O (78:22, v/v), ACN/H_2O (90:10, v/v) and ACN/H_2O (75:25, v/v) yielded policosidin A (**3**, 4.0 mg) from HLW102, policosidin B (**4**, 10.0 mg) from HLW103, poloncosidin A (**5**, 17.0 mg) and poloncosidin B (**8**, 2.0 mg) from HLW105, respectively.

To isolate compounds **6** and **7**, the preparative HPLC fractions from HLW102 (Δ*dovB*) extracts were further separated on a silica gel 60 column with a gradient of CH_2Cl_2 and MeOH from 100:1 to 0:100 (v/v), followed by Semi-preparative HPLC purification using ACN/H_2O (55:45, v/v) as solvent, which yielded policosidin C (**6**, 3.0 mg) and policosidin D (**7**, 4.0 mg).

To isolate the compounds **9**, **10**, and **11**, the EtOAc extract of strain HLW104 (Δ*dovD*) was first fractionated on a preparative HPLC with a linear gradient from 15 to 100% ACN in H_2O in 20 min. Further purification on a semi-preparative HPLC by using

ACN/H_2O (70:30, v/v), ACN/H_2O (60:40, v/v) and ACN/H_2O (75:25, v/v) as elution solvents yielded policosidin E (**9,** 6.0 mg), policosidin F (**10,** 4.0 mg) and policosidin G (**11,** 7.0 mg), respectively.

7. HPLC equipment for analysis and substance isolation

Analysis of EtOAc extracts of the fungal cultures was carried out on an Agilent HPLC series 1200 (Agilent Technologies) equipped with an Agilent Eclipse XDB-C18 column (150 × 4.6 mm, 5 μm) and a flow rate of 0.5 mL/min. A linear gradient from 5% to 100% ACN in H_2O in 30 min was used. The column was then washed with 100% ACN for 5 min and equilibrated with 5% ACN in H_2O for an additional 5 min.

Semi-preparative HPLC was performed on the same system with a VDSpher PUR 100 C18-M-SE column (250 × 10 mm, 5 μm) (VDS optilab Chromatographie Technik) and gradients of ACN in H_2O, at a flow rate of 2.0 mL/min.

Preparative Agilent 1260 Infinity II HPLC was performed with an Agilent Eclipse VDSpher PUR100C18-M-SE (150 × 20 mm, 5 μm) column (Agilent Technologies, Böblingen, Germany) at a flow rate of 15.0 mL/min by using ACN in H_2O as solvents.

8. LC-HRMS analysis for secondary metabolites

LC-MS analysis was carried out on an Agilent HPLC 1260 series system equipped with a Bruker microTOF QIII mass spectrometer by using a VDSpher PUR 100 C18-M-SE column (150 × 2 mm, 3 μm) (VDS optilab Chromatographie Technik). A linear gradient of 5 to 100% ACN in H_2O, both containing 0.1% (v/v) HCOOH, in 10 min and 30 min were used. The column was then washed with 100% ACN for 5 min and equilibrated with 5% ACN in H_2O for 5 min, at a flow rate of 0.3 mL/min.

9. NMR analysis

NMR spectra of the isolated products were recorded on a JEOL ECA-500 MHz spectrometer (JEOL, Akishima, Tokyo, Japan). The samples were dissolved in $CDCl_3$ or DMSO-d_6. All spectra were processed with MestReNov.14.2.1 (Mestrelab Research, Santiago de Compostella, Spain).

10. Structural elucidation

The structures of the known compounds **1**, **2**, **5,** and **8** were determined by comparison of their MS and NMR data with those published in the literature.[7–9] The structures of the new compounds, i.e., **3**, **4**, **6**, **7**, **9**, **10**, and **11**, were elucidated through extensive interpretation of their NMR spectra, particularly their HMBC data and NOESY. If necessary, ECD spectra were also taken and compared with the calculated ones. All the structures are confirmed by their molecular formula deduced from $[M + H]^+$ ions obtained from high-resolution mass spectrometry (Figure S56).
Inspection of the ^{13}C NMR spectra revealed that all the isolated compounds contain an α-pyrone ring with five characteristic signals at δ_C 164–166 for C-1, δ_C 97–110 for C-2,

δ_C 164–169 for C-3, δ_C 106–109 for C-4, and δ_C 158–160 ppm for C-5 (Tables S7–S16). Compound **7** with a $[M + H]^+$ of 387.2038 and a deduced molecular formula of $C_{23}H_{31}O_5$, shows a 14 dalton smaller mass than **1**, indicating the absence of only one methyl group in its structure. Indeed, the signals for the *O*-methyl group in the spectra of **1** at δ_H 3.83 and δ_C 60.4 ppm are absent in those of compound **7**. All these data prove **7** as a demethylated derivative of **1**, termed policosidin D.

In both ^1H and ^{13}C NMR spectra of **3** (policosidin A) and **4** (policosidin B), signals of the epoxytetrahydrofuran ring in **1**, **2**, and **7** disappeared. Instead, signals of a polyene chain with five methylated and conjugated double bonds are detected. In the ^1H NMR spectrum, the olefinic protons are present as four characteristic singlets (δ_H 5.8–6.1 ppm) and one quartet (δ_H 5.47, 7.0 Hz) and methyl group as five singlets (δ_H 1.7–2.1 ppm) and one doublet (δ_H 1.7, 7.0 Hz). The ten olefinic carbon signals are detected in the range of δ_C 125–140 ppm. Interpretation of their HMBC spectra confirmed this conclusion and proved its attachment to the α-pyrone ring at C-5. Compound **4** differs from **3** by a methoxy group instead of a hydroxyl group, which is concluded by detection of signals at δ_H 3.82 and δ_C 60.3 ppm. This difference was also observed for **5** (poloncosidin A) and **6** (policosidin C). The signals at δ_H 3.84 and δ_C 60.4 ppm (in $CDCl_3$) in the spectra of **5** are absent in those of **6**, proving that the hydroxyl group at C-3 in **6** is not methylated.

Comparison of the NMR data of **9** (policosidin E) with those of **4** revealed the up-field shifted signals at δ_H 2.54 and δ_C 40.5 ppm for CH-14 and δ_H 3.67 and δ_C 72.3 ppm for CH-15 (Table S14), proving the disappearance of the double bond at the end of the polyene chain and hydroxylation at C-15. The stereochemistry at C-14 and C-15 was determined by comparison of the experimental and calculated ECD spectra (Figure S54).

Inspection of the NMR data of **10** (policosidin F) indicated the presence of two compounds with very similar structures. Detailed interpretation of the spectra and comparison with those of **9** proved **10** is a mixture of two diastereomers with an additional hydroxyl group at C-14. The signals of the two hydroxylated carbons are detected at δ_C 76.1/76.3 (C-14) and δ_C 73.7/75.0 (C-15) ppm.

Policosidin G (**11**) was isolated as a mixture with **5**. Nevertheless, its signals can be distinguished from those of **5** and its structure can be elucidated, due to the availability of the NMR spectra of **5** as pure compound. The two sets of signals in the mixture have very high similarity and many signals overlap even with each other, suggesting the existence of an isomer pair. NOESY spectrum (Figure S52) of the mixture shows that 12-CH_3 (δ_H 1.46 ppm) in **11** is correlated to 15-CH_3 (δ_H 1.30 ppm), proving **11** is a diastereomer of **5** at C-12.

11. Feeding experiments

Feeding with ^{13}C-labeled precursors

For labeling experiments, the precursor [methyl-^{13}C]-L-methionine and sodium [1-^{13}C] acetate were dissolved in H_2O to a concentration of 10 mg/mL. The wild-type strain *P. polonicum* NRRL 995 was cultivated in rice medium (5 g rice and 3 mL H_2O) at room

temperature. After cultivation for 2 days, 0.8 mL [methyl-^{13}C]-L-methionine or 1.6 mL sodium [1-^{13}C] acetate stock solutions were added to the cultures, resulting in a final concentration of approximately 1 mg/g or 2 mg/g rice, respectively. The blank control was prepared without feeding. After further cultivation for 12 days, the secondary metabolites were extracted with EtOAc twice and then dissolved in methanol for LC-MS analysis.

Precursor supply for *A. nidulans* HLW23 (*dovE*), HLW25 (*dovC*), HLW40 (*dovCD*), and HLW42 (*dovD*) as well as the control strain BK06

For feeding experiments, the precursors **4**, **5**, **9**, and **10** were dissolved in DMSO to give 50 mM stock solutions. The fungal strains were first cultivated in different media for 2 days. 10 µL or 20 µL stock solutions were afterward added to the cultures, to give a final concentration of about 0.1 mM (**5, 9**, and **10**) or 0.2 mM (**4**). After cultivation at room temperature for further 5 days, the secondary metabolites were extracted with EtOAc twice and dissolved in methanol for LC-MS analysis.
For biotransformation of **5** to **1**, the mutant strain HLW23 and the control strain BK06 were cultivated in LMM medium.
For biotransformation of **4** to **9**, **10**, **5** and **11**, the mutant strains HLW25, HLW40, and HLW42 and the control strain BK06 were cultivated in PD medium.
In order to prove whether **9** and **10** can be transformed into **5**. the mutant strain HLW42 and the control strain BK06 were cultivated in PD medium for biotransformation.

12. Preparation of DovC- and DovD-containing lysates from *A. nidulans* transformants

DovC- and DovD-containing lysates were prepared according to previous protocols with slight modifications.[10] Spores of *A. nidulans* strains HLW25 and HLW40, expressing DovC and DovCD, respectively, were inoculated into PD medium with essential supplements and cultured at room temperature for 7 days. Mycelia were harvested, thoroughly washed with pre-chilled sterile water, and ground into a fine powder in liquid nitrogen using a pre-chilled mortar and pestle. The obtained powder was suspended in a buffer containing 50 mM Tris-HCl (pH 7.5), 1 mM EDTA, 10% glycerol, 1 mM DTT, and 1 mM PMSF, at a ratio of 1 g wet cells to 2 mL buffer. The crude lysate was used for subsequent *in vitro* assays.

13. Expression and preparation of DovC- and DovD-containing lysates in *S. cerevisiae*

For expression in *S. cerevisiae*, plasmids carrying DovC (pHLW80) and DovD (pHLW81) under the control of the GAL1 promoter were transformed into *S. cerevisiae* INVSc1. Transformants were selected on SC-Ura. Positive clones were cultured in 250 mL of SC-Ura medium containing 2% glucose at 30 °C and 250 rpm for 36 h. Cells were then washed and transferred to 500 mL of induction medium containing 1% raffinose and 2% galactose, and incubated for an additional 16 h. Harvested cells were collected by centrifugation (5,000 × g, 4 °C, 15 min), flash-frozen in liquid nitrogen, and ground into powder using a mortar and pestle. The resulting materials were

resuspended in buffer (50 mM Tris-HCl, pH 7.5; 15% glycerol; 1 mM DTT; 1 mM PMSF) and directly used for *in vitro* assays.

14. *In vitro* assays of 4 with DovC- and DovD-containing lysates from *A. nidulans* and *S. cerevisiae*

Due to membrane-bound feature and unknown cofactor of DovD, we used total cell lysates for *in vitro* assays. 20 μL of different DovC- or DovCD-containing lysates, 2 mM substrate **4**, 5 mM NADPH and 0.5 mM flavin adenine dinucleotide (FAD) in 50 mM Tris-HCl, pH 7.5, were incubated in a total 50 μL reaction. The reactions were incubated at 30 °C for 2 h and terminated by addition of 50 μL of MeOH. After centrifugation, the supernatants were analyzed on LC-MS.

15. Measurement of optical rotations

The optical rotation was measured on the polarimeter Jasco DIP-370 at 20 °C using the D-line of the sodium lamp at λ=589.3 nm. Prior to the measurement, the polarimeter was calibrated with 10% aqueous glucose solution and methanol as controls.

16. The experimental and calculated electronic circular dichroism (ECD) spectra

The experimental ECD spectra were taken on a J-1500 CD spectrometer (Jasco Deutschland GmbH, Pfungstadt, Germany). The samples were dissolved in MeOH and measured in the range of 200-400 nm by using a 1 mm path length quartz cuvette (Hellma Analytics, Müllheim, Germany).

For ECD calculation, Initial conformational search was performed with the Crest program.[11] Geometry optimizations, frequency analysis, as well as ECD calculation were performed using the Gaussian 16 package (https://gaussian.com/gaussian16/). The obtained conformers were optimized at B3LYP/6-31G(d) and the conformers with populations over 1% were kept. Frequency analysis was also conducted on the same level to exclude the conformers with imaginary frequency. ECD spectra were calculated by the TDDFT methodology at the CAM-B3LYP/TZVP utilizing IEFPCM solvent model in methanol. ECD spectra were simulated using SpecDis software[12] according to Boltzmann distributions of the lowest energy conformers. The experimental and calculated ECD spectra are given in Figures S53–S55.

17. Physiochemical properties of the compounds described in this study

deoxyverrucosidin (**1**): yellow oil, $[\alpha]_D^{20}$= +58 (*c* 0.50, MeOH); ^{1}H NMR and ^{13}C NMR data are given in Table S7 and NMR spectra in Figures S4 and S5, respectively; HRMS(ESI) m/z: [M + H] $^+$ calcd. for $C_{24}H_{33}O_5$ 401.2323; found 401.2339.

nordeoxyverrucosidin (**2**): yellow oil, $[\alpha]_D^{20}$= +83 (*c* 0.30, MeOH); ^{1}H NMR data are given in Table S7 and ^{1}H NMR spectrum in Figure S6, HRMS(ESI) m/z: [M + H] $^+$ calcd. for $C_{23}H_{31}O_5$ 387.2166; found 387.1940.

policosidin A (**3**): pale yellow solid, $[\alpha]_D^{20}$= 0 (*c* 0.10, MeOH); NMR data are given in Table S8 and NMR spectra in Figures S7−S11, respectively, HRMS(ESI) m/z: [M + H] $^+$ calcd. for $C_{23}H_{31}O_3$ 355.2268; found 355.2440.

policosidin B (**4**): yellow oil, $[\alpha]_D^{20}$= 0 (*c* 0.30, MeOH); NMR data are given in Table S9 and NMR spectra in Figures S12−S16, respectively, HRMS(ESI) m/z: $[M + H]^+$ calcd. for $C_{24}H_{33}O_3$ 369.2424; found 369.2445.

poloncosidin A (**5**): pale yellow oil, $[\alpha]_D^{20}$= +108 (*c* 0.43, MeOH); NMR data are given in Table S10 and NMR spectra in Figures S17−S23, respectively, HRMS(ESI) m/z: $[M + H]^+$ calcd. for $C_{24}H_{33}O_4$ 385.2373; found 385.2325.

policosidin C (**6**): yellow oil, $[\alpha]_D^{20}$= +120 (*c* 0.30, MeOH); NMR data are given in Table S11 and NMR spectra in Figures S24−S29, respectively, HRMS(ESI) m/z: $[M + H]^+$ calcd. for $C_{23}H_{31}O_4$ 371.2217; found 371.2216.

policosidin D (**7**): yellow oil, $[\alpha]_D^{20}$= +120 (*c* 0.40, MeOH); NMR data are given in Table S12 and NMR spectra in Figures S30−S35 respectively, HRMS(ESI) m/z: $[M + H]^+$ calcd. for $C_{23}H_{31}O_5$ 387.2166; found 387.2038.

poloncosidin B (**8**): yellow oil, $[\alpha]_D^{20}$= +16 (*c* 0.20, MeOH); ^{1}H NMR data are given in Table S13 and ^{1}H NMR spectrum in Figure S36, HRMS(ESI) m/z: $[M + H]^+$ calcd. for $C_{23}H_{31}O_4$ 371.2217; found 371.2232.

policosidin E (**9**): yellow oil, $[\alpha]_D^{20}$= +17 (*c* 0.60, MeOH); NMR data are given in Table S14 and NMR spectra in Figures S37−S41, HRMS(ESI) m/z: $[M + H]^+$ calcd. for $C_{24}H_{35}O_4$ 387.2530; found 387.2575.

policosidin F (**10**): yellow oil, ^{1}H NMR, ^{13}C NMR and key HMBC correlations are given in Table S15 and NMR spectra in Figures S42−S46, respectively, HRMS(ESI) m/z: $[M + H]^+$ calcd. for $C_{24}H_{35}O_5$ 403.2479; found 403.2523.

policosidin G (**11**): pale yellow oil, ^{1}H NMR, ^{13}C NMR and key HMBC correlations data are given in Table S16 and NMR spectra in Figures S47−S52, HRMS(ESI) m/z: $[M + H]^+$ calcd. for $C_{24}H_{33}O_4$ 385.2373; found 385.2342.

Supplementary Tables

Table S1. Proteins encoded by the *dov* cluster in *Penicillium polonicum* NRRL995.

P. polonicum NRRL995	Length	Location at JAPDKX010000002.1	Putative function
DovA	2566 aa	(789192-790344,790401-796948)	Polyketide synthase
DovB	228 aa	(803942,804628)	Methyltransferase
DovC	467 aa	(801573-802039,802101-802469,802529-802802,802891-803181)	Flavin-containing monooxygenase
DovD	368 aa	(800247-800602,800667-801414)	Cyclase (epoxide expandase)
DovE	520 aa	(797742-799191,79253-799316,799378-799405,799460-799480)	Cytochrome P450
DovF	576 aa	(785533-786211,786261-786514,786566-786672,786727-787022,787079-787473)	Transcription Factor
DovG	805 aa	(805226-805291,805345-805437,805492-805536,805593-805678,805738-806006,806061-807012,807072-807230,807300-808047)	Flavin-containing monooxygenase

Table S2. Sequence identities of nucleotides of the *dov* cluster from *Penicillium polonicum* NRRL995 with those of sequenced *Penicillium polonicum* strains as well as other *Penicillium* species.

Strain	*dov* cluster	Annotation	Location	Length	Identities
P. polonicum NRRL995	√	-	JAPDKX010000002.1 785533-808047	22515	
P. polonicum IBT4502	√	√	MDYM01000002.1 1255533-1233019	22515	99%
P. polonicum IIF1SW-F3	-	-	-	-	-
P. polonicum PB2502	√	-	JAMFOP010000007.1 305517-282992	22526	99%
P. polonicum PM3203	√	-	JAMFON010000022.1 288665-311190	22526	99%
P. polonicum R2416	√	-	JAMFOO010000008.1 635066-657591	22526	99%
P. polonicum CGMCC3.15272	√	-	JANFQU010000017.1 91768-69243	22526	99%
P. polonicum CGMCC3.15264	-	-	-	-	-
P. polonicum hy4	-	-	-	-	-
P. polonicum F7	√	-	CM024081.1 2316167-2338692	22526	99%
P. polonicum KACC93368	√	-	CP139884.1 2380636-2403161	22526	99%
Penicillium sp. CMV-2018d	√	√	JAPZBW010000001.1 2366939-2392168	25230	97%
P. glandicola 3C*	√	√	CACTIM020000215.1 7139-26114	18976	85%

*: This *dov* cluster does not contain a transcription factor.

Table S3. Sequence identities of proteins encoded by the *dov* cluster from *Penicillium polonicum* NRRL995 with those of known clusters.

Penicillium polonicum NRRL995	*Penicillium polonicum* X6	*Aspergillus terreus* NIH2624	*Aspergillus terreus var. aureus* strain CBS 503.65	*Calcarisporium arbuscula* NRRL3705	*Emericella variecolor* NHL 2881	Putative Function
DovA	VerA PENPOL_C002G03804 (100/99.72)	XP_001218239.1 (95/44.87)	CtvA (95/45.65)	AurA (97/49.08)	AstA (97/48.42)	Polyketide synthase
DovB	VerB PENPOL_C002G01780 (100/100.00)	XP_001218240.1 (96/49.32)	CtvB (96/49.55)	AurB (96/43.24)	AstB (96/38.77)	Methyltransferase
DovC	VerC2 PENPOL_C002g07872 (73/97.47)	XP_001218242.1 (89/47.47)	CtvC (90/47.39)	AurC (96/41.61)	AstC (90/42.48)	Flavin-containing monooxygenase
DovD	VerU1 PENPOL_c002G01858 (97/95.80)	-	-	-	-	Cyclase (epoxide expandase)
DovE	VerH PENPOL_c002G07307 (93/100.00)	-	-	-	-	Cytochrome P450
DovF	VerF PENPOL_c002G07024 (100/99.83)	-	-	AurF -	-	Transcription Factor
DovG	VerC1 PENPOL_C002g07872 (100/99.88)	XP_001218242.1 (49/34.06)	CtvC (49/34.39)	AurC (51/31.52)	AstC (51/33.98)	Flavin-containing monooxygenase

The identity of each homolog to *Penicillium polonicum* NRRL995 is shown as (Coverage/identity, %).

Table S4. Strains used in this study.

Strain	Genotype	Source
E.coli		
DH5α	F^-*endA1 glnV44 thi-1 recA1 relA1 gyrA96 deoR nupG purB20* φ80dl*acZ*ΔM15 Δ(*lacZYA-argF*)U169, hsdR17($r_K^- m_K^+$), λ^-	[1]
S. cerevisiae		
BJ5464-npgA	*MATα ura3-52 leu2-Δ1 trp1 his3-Δ200 pep4::HIS3 prb1-Δ1.6R can1 GAL npgA*	[13]
INVSc1	*MATa his3Δ1 leu2 trp1-289 ura3-52/MATα his3Δ1 leu2 trp1-289 ura3-52*	[14]
Penicillium polonicum		
NRRL995	wild type	NRRL*
HLW101	Δ*dovA* in NRRL995	This study
HLW102	Δ*dovB* in NRRL995	This study
HLW103	Δ*dovC* in NRRL995	This study
HLW104	Δ*dovD* in NRRL995	This study
HLW105	Δ*dovE* in NRRL995	This study
HLW106	Δ*dovG* in NRRL995	This study
Aspergillus nidulans		
LO8030	*pyroA4, riboB2, pyrG89, nkuA::argB*, sterigmatocystin cluster (*AN7804 − AN7825*)Δ, emericellamide cluster (*AN2545 − AN2549*)Δ, asperfuranone cluster (*AN1039 − AN1029*)Δ, monodictyphenone cluster (*AN10023 − AN10021*)Δ, terrequinone cluster (*AN8512 − AN8520*)Δ, austinol cluster part 1 (*AN8379 − AN8384*)Δ, austinol cluster part 2 (*AN9246 − AN9259*)Δ, F9775 cluster (*AN7906 − AN7915*)Δ, asperthecin cluster (*AN6000 − AN6002*)Δ	[15]
BK06	Δ*wA*-PKS::*gpdA*(p)-*Afribo* in LO8030	[16]
ZL132	Δ*wA*-PKS::*gpdA*(p)-*Afribo-hlyA*(p)-*gpdA*$^{\#}$(p) in LO8030	[6]
HLW01	Δ*wA*-PKS::*gpdA*(p):*dovA-Afribo* in LO8030	This study
HLW16	Δ*wA*-PKS::*gpdA*(p):*dovABCDEFG-Afribo* in LO8030	This study
HLW23	Δ*wA*-PKS::*gpdA*(p):*dovE-Afribo* in LO8030	This study
HLW25	Δ*wA*-PKS::*gpdA*(p):*dovC-Afribo* in LO8030	This study
HLW26	Δ*wA*-PKS::*gpdA*(p):*dovABCDEF-Afribo* in LO8030	This study
HLW40	Δ*wA*-PKS::*gpdA*(p):*dovCD-Afribo* in LO8030	This study
HLW42	Δ*wA*-PKS::*gpdA*(p):*dovD-Afribo* in LO8030	This study
HLW59	Δ*wA*-PKS::*gpdA*(p):*dovA-Afribo-hlyA*(p)-*gpdA*$^{\#}$(p):*dovC* in LO8030	This study
HLW60	Δ*wA*-PKS::*gpdA*(p):*dovA-Afribo-hlyA*(p):*dovB-gpdA*$^{\#}$(p) in LO8030	This study
HLW61	Δ*wA*-PKS::*gpdA*(p):*dovA-Afribo-hlyA*(p):*dovB-gpdA*$^{\#}$(p):*dovC* in LO8030	This study

*NRRL: ARS Culture Collection

Table S5. Plasmids used and constructed in this study.

Plasmid	Description	Source
pJN017	*URA3, wA flanking, gpdA*(*p*), *afriboB, ampR*	16
pZL132	*URA3, wA flanking, gpdA*(*p*), *hlyA*(p), *gpdA*(p) from pYTR, *afriboB, ampR*	6
p5HY	Two-third of the hph resistance gene at the 5´-end, originated from the pUChph and inserted into pESC-URA. For gene replacement using hph as selection marker	5
p3YG	Two-third of the hph resistance gene at the 3´-end, originated from the pUChph and inserted into pESC-URA. For gene replacement using hph as selection marker.	5
pHLW01 (pJN017-*dovA*)	an 8361 bp fragment of *dovA* with its terminator from genomic DNA of *P. polonicum* NRRL 995 inserted in pJN017	This study
pHLW02 (p5HY-*dovA*)	a 1551 bp upstream PCR fragment of *dovA* from genomic DNA of *P. polonicum* NRRL995 inserted in p5HY	This study
pHLW03 (p3YG-*dovA*)	a 1508 bp downstream PCR fragment of *dovA* from genomic DNA of *P. polonicum* NRRL995 inserted in p3YG	This study
pHLW06 (p5HY-*dovB*)	a 1463 bp upstream PCR fragment of *dovB* from genomic DNA of *P. polonicum* NRRL995 inserted in p5HY	This study
pHLW07 (p3YG-*dovB*)	a 1494 bp downstream PCR fragment of *dovB* from genomic DNA of *P. polonicum* NRRL995 inserted in p3YG	This study
pHLW10 (p5HY-*dovG*)	a 1458 bp upstream PCR fragment of *dovG* from genomic DNA of *P. polonicum* NRRL995 inserted in p5HY	This study
pHLW11 (p3YG-*dovG*)	a 1225 bp downstream PCR fragment of *dovG* from genomic DNA of *P. polonicum* NRRL995 inserted in p3YG	This study
pHLW12 (p5HY-*dovE*)	a 1528 bp upstream PCR fragment of *dovE* from genomic DNA of *P. polonicum* NRRL995 inserted in p5HY	This study
pHLW13 (p3YG-*dovE*)	a 1445 bp downstream PCR fragment of *dovE* from genomic DNA of *P. polonicum* NRRL995 inserted in p3YG	This study
pHLW16 (pJN017-*dovABCDEFG*)	A 24137 bp fragment of *dovABCDEFG* with its terminator from genomic DNA of *P. polonicum* NRRL 995 inserted in pJN017	This study
pHLW21 (p5HY-*dovC*)	a 1260 bp upstream PCR fragment of *dovC* from genomic DNA of *P. polonicum* NRRL995 inserted in p5HY	This study
pHLW22 (p3YG-*dovC*)	a 1187 bp downstream PCR fragment of *dovC* from genomic DNA of *P. polonicum* NRRL995 inserted in p3YG	This study
pHLW23 (pJN017-*dovE*)	a 2684 bp fragment of *dovE* with its terminator from genomic DNA of *P. polonicum* NRRL 995 inserted in pJN017	This study

Table S5. Plasmids used and constructed in this study (continued).

Plasmid	Description	Source
pHLW25 (pJN017-*dovC*)	a 2113 bp fragment of *dovC* with its terminator from genomic DNA of *P. polonicum* NRRL 995 inserted in pJN017	This study
pHLW26 (pJN017-*dovABCDEF*)	a 20652 bp fragment of *dovABCDEF* with its terminator from genomic DNA of *P. polonicum* NRRL 995 inserted in pJN017	This study
pHLW38 (p5HY-*dovD*)	a 1220 bp upstream PCR fragment of *dovD* from genomic DNA of *P. polonicum* NRRL995 inserted in p5HY	This study
pHLW39 (p3YG-*dovD*)	a 1159 bp downstream PCR fragment of *dovD* from genomic DNA of *P. polonicum* NRRL995 inserted in p3YG	This study
pHLW40 (pJN017-*dovCD*)	a 3401 bp fragment of *dovCD* from genomic DNA of *P. polonicum* NRRL 995 inserted in pJN017	This study
pHLW42 (pJN017-*dovD*)	a 1690 bp fragment of *dovD* with its terminator from genomic DNA of *P. polonicum* NRRL 995 inserted in pJN017	This study
pHLW56 (pZL132-*dovB*)	a 1073 bp fragment of *dovB* with its terminator from genomic DNA of *P. polonicum* NRRL 995 inserted in pZL132	This study
pHLW57 (pZL132-*dovC*)	a 2118 bp fragment of *dovC* with its terminator from genomic DNA of *P. polonicum* NRRL 995 inserted in pZL132	This study
pHLW58 (pZL132-*dovBC*)	a 1073 bp fragment of *dovB* with its terminator and a 2118 bp fragment of *dovC* with its terminator from genomic DNA of *P. polonicum* NRRL 995 inserted in pZL132	This study
pHLW59 (pZL132-*dovAC*)	an 8343 bp fragment of *dovA* with its terminator and a 2118 bp fragment of *dovC* with its terminator from genomic DNA of *P. polonicum* NRRL 995 inserted in pZL132	This study
pHLW60 (pZL132-*dovAB*)	an 8343 bp fragment of *dovA* with its terminator and a 1073 bp fragment of *dovB* with its terminator from genomic DNA of *P. polonicum* NRRL 995 inserted in pZL132	This study
pHLW61 (pZL132-*dovABC*)	an 8343 bp fragment of *dovA* with its terminator, a 1073 bp fragment of *dovB* with its terminator, and a 2118 bp fragment of *dovC* with its terminator from genomic DNA of *P. polonicum* NRRL 995 inserted in pZL132	This study
pHLW80 (pYES2-NT-B-DovC)	a 1401 bp fragment of *dovC* from complementary DNA of *P. polonicum* NRRL 995 inserted in pYES2-NT-B	This study
pHLW81 (pYES2-NT-B-DovD)	a 1104 bp fragment of *dovD* from complementary DNA of *P. polonicum* NRRL 995 inserted in pYES2-NT-B	This study

Table S6. Primers used in this study.

Primer	Oligonucleotide sequence 5'-3'	Function
HLW_pHLW01-1-F	GCTTGACTAACAGCTACCCCGCTTGAGCAGACATCACCatgacaccagaacctattgct	Amplification of the two fragments of *dovA* gene and its terminator from *P. polonicum* NRRL995 to construct pHLW01, pHLW59, pHLW60, and pHLW61; Screening of *gpdA*::*dovA* transformants
HLW_pHLW01-1-R	cgcagcatattggctcctac	
HLW_pHLW01-2-F	gcaagtggtggatcaacttac	
HLW_pHLW01-2-R	CCAGCCTCAACACCATATTTTAATCCCATGTGGGCGCCcatacgatgcaagttgctgac	
HLW_pHLW02-F	AAGAATTGTTAATTAAGAGCTCAGATCcgtcagatcgaggttcttgctg	Amplification of the upstream region of *dovA* from *P. polonicum* NRRL995 to construct pHLW02
HLW_pHLW02-R	CCCTCACTAAAGGGCGGCCGCACTAGgcgatttcaatgatttgagttcgc	
HLW_pHLW03-F	ACTCACTATAGGGCCCGGGCGgcgactagtggattcctgtatg	Amplification of the downstream region of *dovA* from *P. polonicum* NRRL995 to construct pHLW03
HLW_pHLW03-R	TAGCCGCGGTACCAAGCTTACTCGActgatcgcaactggatgcaac	
HLW_5-Hygsplit-R	TCGAAGTAGCGCGTCTGC	Amplification of 2/3 from the *hph* gene (5'end) for split marker cloning
HLW_3-Hygsplit-F	TTGGGGAATTCAGCGAGAGC	Amplification of 2/3 from the *hph* gene (3'end) for split marker cloning
HLW_verA-UP-V-F	gttcaatgatcgatgcagtcctc	Screening of Δ*dovA* transformants
HLW_verA-Down-V-R	ctagtatgcgccaggttaatg	Screening of Δ*dovA* transformants
HLW_pHLW06-F	AAGAATTGTTAATTAAGAGCTCAGATCcgtggtcatagatgaagtcgg	Amplification of the upstream region of *dovB* from *P. polonicum* NRRL995 to construct pHLW06
HLW_pHLW06-R	ACCCTCACTAAAGGGCGGCCGCACTAGggagagttgatacggttgctg	
HLW_pHLW07-F	ACTCACTATAGGGCCCGGGCGgcattgtacctgtaacttgggt	Amplification of the downstream region of *dovB* from *P. polonicum* NRRL995 to construct pHLW07
HLW_pHLW07-R	TAGCCGCGGTACCAAGCTTACTCGAcagctgtattcggcagacag	
HLW_verB-inside-R	catggtctatggatggcactg	Screening of Δ*dovB* transformants
HLW_verB-Down-V-R2	cgtcaacctgtcatagctgc	Screening of Δ*dovB* transformants
HLW_verB-UP-V-F2	cggtccatcttctgcaagatg	Screening of Δ*dovB* transformants
HLW_pHLW10-F	AAGAATTGTTAATTAAGAGCTCAGATCcacgcgaagtgttcagtatgag	Amplification of the upstream region of *dovG* from *P. polonicum* NRRL995 to construct pHLW10
HLW_pHLW10-R	ACCCTCACTAAAGGGCGGCCgactctggagagtacgaacagtc	
HLW_pHLW11-F	ACTCACTATAGGGCCCGGGCGgcttggtgtgtactgcctag	Amplification of the downstream region of *dovG* from *P. polonicum* NRRL995 to construct pHLW11
HLW_pHLW11-R	TAGCCGCGGTACCAAGCTTACTCGAgcgtagttagttgcactagtcg	

Table S6. Primers used in this study (continued).

HLW_verC1-V-F	ctggccttactctagccagtg	Screening of Δ*dovG* transformants
HLW_verC1-V-R	gcgaggttctcttccgtaacag	Screening of Δ*dovG* transformants
HLW_verC1-V-F2	cgagcctagatgctgtcattg	Screening of Δ*dovG* transformants
HLW_verC1-V-R2	ctctgccacttcttggctattc	Screening of Δ*dovG* transformants
HLW_pHLW12-F	AAGAATTGTTAATTAAGAGCTCAGATCcaacagccagcctattaaccg	Amplification of the upstream region of *dovE* from *P. polonicum* NRRL995 to construct pHLW12
HLW_pHLW12-R	ACCCTCACTAAAGGGCGGCCgcctagaagcaagttccgg	
HLW_pHLW13-F	ACTCACTATAGGGCCCGGGCGcactcgatatgagctacagcg	Amplification of the downstream region of *dovE* from *P. polonicum* NRRL995 to construct pHLW13
HLW_pHLW13-R	TAGCCGCGGTACCAAGCTTACTCGActgcaggttggatgttgagatg	
HLW_verH-V-F	gtcacgtggaatgaccgc	Screening of Δ*dovE* transformants
HLW_verH-V-R	catctagcgtacatgtcaaccg	Screening of Δ*dovE* transformants
HLW_verH-V-F2	gttgctgtgaagagaatgcg	Screening of Δ*dovE* transformants
HLW_verH-V-R2	gttcgtccatcttggttcacac	Screening of Δ*dovE* transformants
HLW_pHLW16-1-F	GACTAACAGCTACCCCGCTTGAGCAGACATCACCcgagcttgctaatgacgtcaatg	Amplification of the DNA region of *dovABCDEFG* and its terminator from *P. polonicum* NRRL995 to construct pHLW16
HLW_pHLW04-3-R	gtagcttacctgatgttaatgcg	
HLW_pHLW04-4-F	cgagacatctgaactacagcg	
HLW_pHLW04-4-R	gtcctacatgcgactaagtcaatg	
HLW_pHLW04-5-F	caacgtggtaatagtgtcaatgc	
HLW_pHLW04-5-R	gatcgtagtatgttcttggcgatag	
HLW_pHLW16-4-F	cttcagacacagctactacgc	
HLW_pHLW16-4-R	ggtatgaggatgccgagttg	
HLW_pHLW16-5-F	caactgcgctaggattcgag	
HLW_pHLW16-5-R2	CCTCAACACCATATTTTAATCCCATGTGGGCGCCgctgatcgtcgacgttatcaag	
HLW_verC2-p5HY-F	AAGAATTGTTAATTAAGAGCTCAGATCcgttcgattcactctcgcttatg	Amplification of the upstream region of *dovC* from *P. polonicum* NRRL995 to construct pHLW21
HLW_verC2-p5HY-R	ACCCTCACTAAAGGGCGGCCcaactgcgctaggattcgagac	
HLW_verC2-p3YG-F	ACTCACTATAGGGCCCGGGCGccagcctattaaccggatcctg	Amplification of the downstream region of *dovC* from *P. polonicum* NRRL995 to construct pHLW22
HLW_verC2-p3YG-R	TAGCCGCGGTACCAAGCTTACTCGAgaatcatcagaatgtccacagcag	
HLW_verC2-F	caattctcacacggcaacaac	Screening of Δ*dovC* transformants
HLW_verC2-R	ggtcgtcttccaaggcatatc	Screening of Δ*dovC* transformants

Table S6. Primers used in this study (continued).

HLW_verC2-V-R	gcatctggtctgtcattgatgg	Screening of Δ*dovC* transformants
HLW_pHLW23-F	AGCTTGACTAACAGCTACCCCGCTTGAGCAGACATCACCatggcttcgttctcccggag	Amplification of the *dovE* gene and its terminator from *P. polonicum* NRRL995 to construct pHLW23
HLW_pHLW23-R	CCAGCCTCAACACCATATTTTAATCCCATGTGGGCGCCcatgtactcaaccagtggacg	
HLW_pHLW25-F	GCTACCCCGCTTGAGCAGACATCACCatggagaaggctcatttcaagg	Amplification of the *dovC* gene and its terminator from *P. polonicum* NRRL995 to construct pHLW25
HLW_pHLW25-R	AACACCATATTTTAATCCCATGTGgcttatctcattgctcgctgattcg	
HLW_pHLW26-5-R	CCATATTTTAATCCCATGTGGGCGCCctggagagtacgaacagtcgtac	Amplification of the DNA region of *dovABCDEF* from *P. polonicum* NRRL995 to construct pHLW26
HLW_pHLW38-F	AAGAATTGTTAATTAAGAGCTCAGATCgcatgtggtcattgtcacacac	Amplification of the upstream region of *dovD* from *P. polonicum* NRRL995 to construct pHLW38
HLW_pHLW38-R	ACCCTCACTAAAGGGCGGCCccatcaatgacagaccagatgc	
HLW_pHLW39-F	ACTCACTATAGGGCCCGGGCGggctggctgttgtactatcactag	Amplification of the downstream region of *dovD* from *P. polonicum* NRRL995 to construct pHLW39
HLW_pHLW39-R	TAGCCGCGGTACCAAGCTTACTCGAcgcagacaatcaacaacctgg	
HLW_verU1-F	cattcgatgcagcctgctcttg	Screening of Δ*dovD* transformants
HLW_verU1-R	gcaccacaaggcgacatcattac	Screening of Δ*dovD* transformants
HLW_verU1-V-F	ctggtgatgatgctagtgctcg	Screening of Δ*dovD* transformants
HLW_verU1-V-R	gttcacagtgtgacacgctc	Screening of Δ*dovD* transformants
HLW_pHLW40-F	GCTACCCCGCTTGAGCAGACATCACCatggagaaggctcatttcaagg	Amplification of the genes *dovC* and *dovD* from *P. polonicum* NRRL995 to construct pHLW40
HLW_pHLW40-R	CACCATATTTTAATCCCATGTGGGCGCCcggttgacatgtacgctagatg	
HLW_pHLW42-F	CAGCTACCCCGCTTGAGCAGACATCACCatgtccacagcagtaaataccattc	Amplification of the *dovD* gene and its terminator from *P. polonicum* NRRL995 to construct pHLW42
HLW_pHLW42-R	CACCATATTTTAATCCCATGTGGGCGCCgccgtcatatgacgagatcc	
HLW_pHLW56-F	ACATCAATCACCCTTCACACCACAACACCatgcatcccagcgccagac	Amplification of the *dovB* gene and its terminator from *P. polonicum* NRRL995 to construct pHLW56
HLW_pHLW56-R	catagacacatctaaacaTAAAgcggccgccgagcctagatgctgtcattg	
HLW_pHLW57-F	TCACACCACAACACCgcggccgcTTTAccattgcttatctcattgctcgc	Amplification of the *dovC* gene and its terminator from *P. polonicum* NRRL995 to construct pHLW57, pHLW58, pHLW59, and pHLW61
HLW_pHLW57-R	taccccgccacatagacacatctaaacaatggagaaggctcatttcaagg	
HLW_pHLW58-B-R2	gcaatgagataagcaatggTAAAgcggccgccgagcctagatgctgtcattgattct	Amplification of the *dovB* gene and its terminator from *P. polonicum* NRRL995 to construct pHLW58, pHLW60, and pHLW61

Table S6. Primers used in this study (continued).

HLW_pHLW80-F	ATCTGTACGACGATGACGATAAGGTACCTAAGatggagaaggctcatttcaagg	Amplification of the *dovC* gene from complementary DNA of *P. polonicum* NRRL995 to construct pHLW80
HLW_pHLW80-R	TGCTGGATATCTGCAGAATTGATCtcaaataaaactatagattaagacaacg	
HLW_pHLW81-F	TGTACGACGATGACGATAAGGTACCTAAGatgtccacagcagtaaataccattc	Amplification of the *dovD* gene from complementary DNA of *P. polonicum* NRRL995 to construct pHLW81
HLW_pHLW81-R	ACTGTGCTGGATATCTGCAGAATTGATCttaaccggatcctgatttgtctcc	

Table S7. NMR data of deoxyverrucosidin (**1**) and nordeoxyverrucosidin (**2**) in $CDCl_3$.

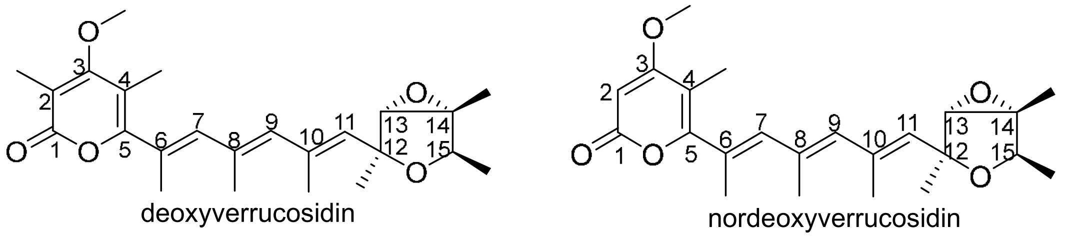

Position	δ_H, multi., *J* in Hz	δ_C	Position	δ_H, multi., *J* in Hz
1		165.7	1	
2		110.3	2	5.48, s
3		168.7	3	
4		109.2	4	
5		159.5	5	
6		127.6	6	
7	6.07, br s	139.2	7	6.07, br s
8		135.1	8	
9	5.86, br s	136.4	9	5.86, s
10		132.2	10	
11	5.51, br s	133.6	11	5.51, s
12		80.3	12	
13	3.45, s	67.6	13	3.45, s
14		67.5	14	
15	4.14, q, 6.8	76.8	15	4.14, q, 6.8
2-CH_3	2.05, s	10.4	3-OCH_3	3.84, s
3-OCH_3	3.83, s	60.4	4-CH_3	1.96 [a]
4-CH_3	2.01, s	12.1	6-CH_3	2.07, d, 1.5
6-CH_3	2.07, d, 1.5	16.8	8-CH_3	1.95, s
8-CH_3	1.97, d, 1.5	18.7	10-CH_3	1.96 [a]
10-CH_3	1.96, d, 1.5	18.6	12-CH_3	1.43, s
12-CH_3	1.43, s	22.0	14-CH_3	1.48, s
14-CH_3	1.48, s	13.9	15-CH_3	1.21, d, 6.8
15-CH_3	1.21, d, 6.8	19.0		

[a]: signals overlap with each other.

The ^1H NMR data of **1** and **2** correspond well to those of deoxyverrucosidin and nordeoxyverrucosidin, respectively.[7,8]

Table S8. NMR data of policosidin A (**3**) in DMSO-d_6.

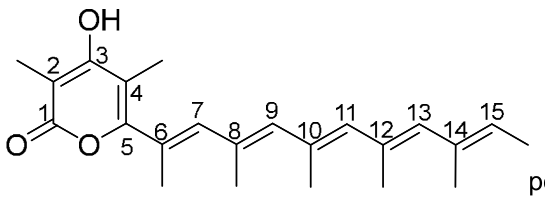

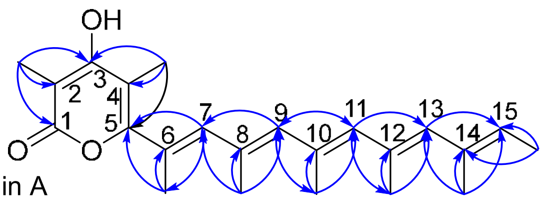

policosidin A

Position	δ_H, multi., *J* in Hz	δ_C	Key HMBC correlations
1		164.8[b]	
2		97.3[b]	
3		167.2[b]	
4		107.5[b]	
5		158.4[b]	
6		127.5[b]	
7	6.11, br s	139.1	C-5 (158.4), 6-CH_3 (17.1)
8		132.0	
9	6.03, br s	137.9	C-7 (139.1), C-11 (136.9), 10-CH_3 (19.5)
10		132.6	
11	5.94, br s	136.9	C-9 (137.9), C-13 (135.2), 12-CH_3 (19.4)
12		132.2	
13	5.86, br s	135.2	C-15 (125.7)
14		133.8	
15	5,47, q, 7.0	125.7	
2-CH_3	1.82, s	9.9	C-1 (164.8), C-2 (97.3), C-3 (167.2)
4-CH_3	1.91, s	12.4	C-3 (167.2), C-4 (107.5), C-5 (158.4)
6-CH_3	2.01[a]	17.1	C-5 (158.4), C-6 (127.5), C-7 (139.1)
8-CH_3	2.01[a]	19.1	C-7 (139.1), C-8 (132.0), C-9 (137.9)
10-CH_3	1.96, br s	19.5	C-9, C-10, C-11
12-CH_3	1.92, br s	19.4	C-11, C-12, C-13
14-CH_3	1.76, br s	17.1	C-13, C-14, C-15
15-CH_3	1.69, d, 7.0	14.2	C-14, C-15

[a]: signals overlap with each other
[b]: signals were deduced from HMBC

Table S9. NMR data of policosidin B (**4**) in $CDCl_3$.

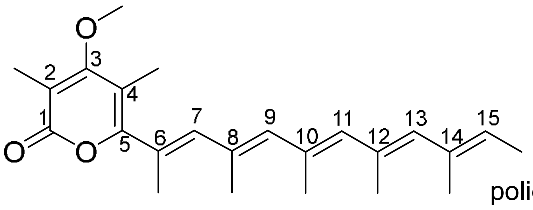

policosidin B

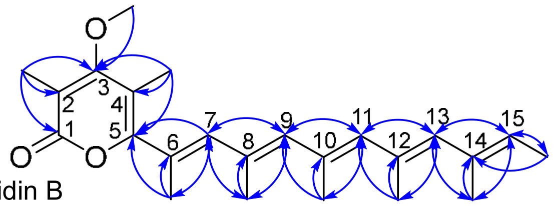

Position	δ_H, multi., *J* in Hz	δ_C	Key HMBC correlations
1		165.8	
2		110.2	
3		168.8	
4		109.2	
5		159.9	
6		126.9	
7	6.10, br s	140.1	C-5, C-9, 6-CH_3, 8-CH_3
8		131.8	
9	5.97, br s	138.1	C-7, C-11, 8-CH_3
10		132.4	
11	5.90, br s	136.8	C-9, C-13, 10-CH_3
12		132.0	
13	5.84, br s	135.0	C-11, C-15, 12-CH_3, 14-CH_3
14		133.7	
15	5,47, q, 7.0	125.4	C-13, 14-CH_3, 15-CH_3
2-CH_3	2.04, s	10.4	C-1, C-2, C-3
3-OCH_3	3.82, s	60.3	C-3
4-CH_3	2.01, s	12.2	C-3, C-4, C-5
6-CH_3	2.08, d, 1.5	16.8	C-5, C-6, C-7
8-CH_3	2.01, d, 1.5	18.9	C-7, C-8, C-9
10-CH_3	1.97, d, 1.5	19.3	C-9, C-10, C-11
12-CH_3	1.93, br s	19.2	C-11, C-12, C-13
14-CH_3	1.77, br s	16.9	C-13, C-14, C-15
15-CH_3	1.71, d, 7.0	14.0	C-14, C-15

Table S10. NMR data of poloncosidin A (**5**) in DMSO-d_6 and $CDCl_3$.

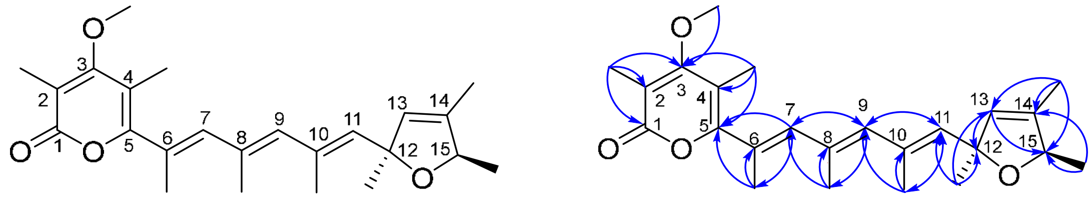

poloncosidin A

Position	δ_H, multi., *J* in Hz (DMSO-d_6)	δ_H, multi., *J* in Hz ($CDCl_3$)	δ_C ($CDCl_3$)	Key HMBC correlations ($CDCl_3$)
1			165.8	
2			110.2	
3			168.8	
4			109.2	
5			159.8	
6			127.1	
7	6.16, br s	6.08, br s	139.7	C-5, C-9, 6-CH_3
8			131.7	
9	5.93, br s	5.87, br s	137.2	C-7, C-11, 8-CH_3
10			131.7	
11	5.53, br s	5.57, br s	137.2	C-9, 10-CH_3
12			88.0	
13	5.61, s	5.53, s	127.4	C-12, C-15
14			138.8	
15	4.62, q, 6.4	4.72, q, 6.0	82.5	
2-CH_3	1.93, s	2.06, s	10.4	C-1, C-2, C-3
3-OCH_3	3.82, s	3.84, s	60.4	C-3
4-CH_3	1.96, s	2.02, s	12.2	C-3, C-4, C-5
6-CH_3	2.00, d, 1.5	2.07, d, 1.5	16.8	C-5, C-6, C-7
8-CH_3	1.95, d, 1.5	1.97, d, 1.5	18.6	C-7, C-8, C-9
10-CH_3	1.88, d, 1.5	1.91, d, 1.5	18.3	C-9, C-10, C-11
12-CH_3	1.33, s	1.43, s	27.9	C-11, C-12, C-13
14-CH_3	1.66, s	1.71, s	12.4	C-13, C-14, C-15
15-CH_3	1.16, d, 6.4	1.28, d, 6.0	20.8	C-14, C-15

The ^1H NMR data of **5** in DMSO-d_6 correspond well to those of poloncosidin A.[9]

Table S11. NMR data of policosidin C (**6**) in $CDCl_3$.

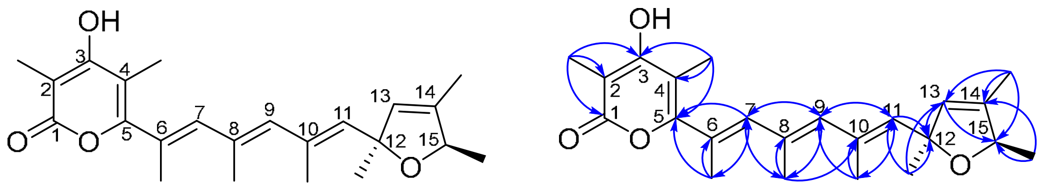

policosidin C

Position	δ_H, multi., *J* in Hz	δ_C	Key HMBC correlations
1		165.5	
2		98.9	
3		164.1[a]	
4		106.2	
5		159.9	
6		126.9	
7	6.03, br s	139.7	C-5, C-9, 6-CH_3, 8-CH_3
8		131.7	
9	5.84, br s	137.2	C-7, C-11, 8-CH_3
10		131.7	
11	5.54, br s	137.1	C-9, C-12, 10-CH_3
12		88.0	
13	5.50, br s	127.4	C-12, C-15
14		138.8	
15	4.72, q, 6.5	82.5	
2-CH_3	1.99, s	8.7	C-1, C-2, C-3
4-CH_3	2.02, s	11.6	C-3, C-4, C-5
6-CH_3	2.04, d, 1.5	16.7	C-5, C-6, C-7
8-CH_3	1.93, d, 1.5	18.5	C-7, C-8, C-9, C-10
10-CH_3	1.87, d, 1.5	18.3	C-9, C-10, C-11
12-CH_3	1.40, s	27.9	C-11, C-12, C-13
14-CH_3	1.68, dd, 1.2, 1.6	12.4	C-13, C-14, C-15
15-CH_3	1.25, d, 6.5	20.7	C-14, C-15

[a]: signal was deduced from HMBC.

Table S12. NMR data of policosidin D (**7**) in DMSO-d_6.

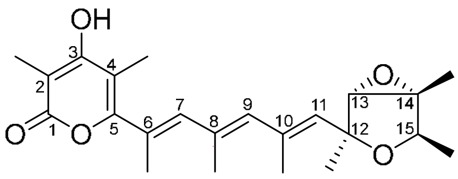

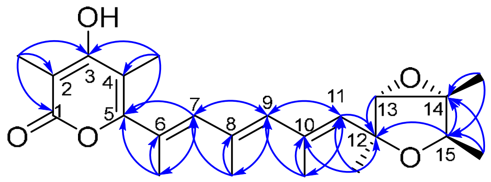

policosidin D

Position	δ_H, multi., J in Hz	δ_C	Key HMBC correlations
1		163.9	
2		97.9	
3		165.2	
4		106.7	
5		157.9	
6		126.9	
7	6.09, br s	138.3	C-5, C-9, 6-CH_3
8		131.8	
9	5.91, br s	136.2	C-7, C-11, 8-CH_3
10		133.4	
11	5.55, br s	134.3	C-9, C-12, 10-CH_3
12		79.4	
13	3.61, s	66.3	C-12
14		66.9	
15	3.99, q, 7.0	75.9	C-12, C-14
2-CH_3	1.84, s	9.2	C-1, C-2, C-3
4-CH_3	1.92, s	11.7	C-3, C-4, C-5
6-CH_3	1.98, d, 1.5	16.4	C-5, C-6, C-7
8-CH_3	1.95, d, 1.5	18.1	C-7, C-8, C-9
10-CH_3	1.91, d, 1.5	18.2	C-9, C-10, C-11
12-CH_3	1.27, s	21.8	C-10, C-11, C-12
14-CH_3	1.38, s	13.4	C-14, C-15
15-CH_3	1.10, d, 7.0	18.6	C-14, C-15

Table S13. NMR data of poloncosidin B (**8**) in DMSO-d_6.

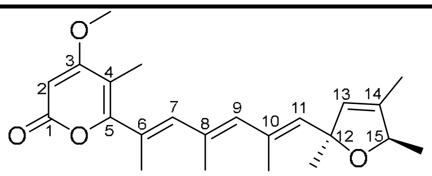

poloncosidin B

Position	δ_H, multi., *J* in Hz
1	
2	5.61, s
3	
4	
5	
6	
7	6.13, br s
8	
9	5.93, br s
10	
11	5.53, br s
12	
13	5.61, s
14	
15	4.61, q, 6.4
3-OCH_3	3.84, s
4-CH_3	1.89, s
6-CH_3	2.00, d, 1.5
8-CH_3	1.95, d, 1.5
10-CH_3	1.87, d, 1.5
12-CH_3	1.33, s
14-CH_3	1.66, s
15-CH_3	1.16, d, 6.4

The ^{1}H NMR data of **8** correspond well to those of poloncosidin B.[9]

Table S14. NMR data of policosidin E (**9**) in $CDCl_3$.

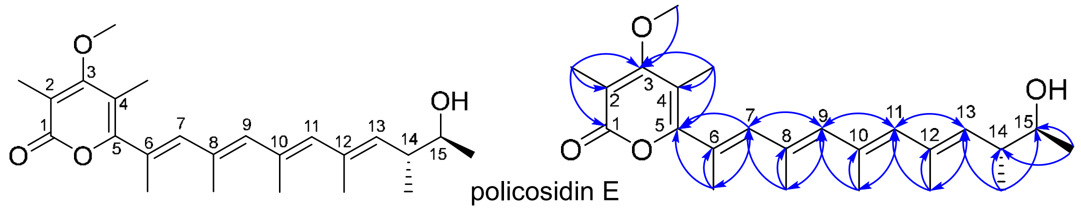

Position	δ_H, multi., J in Hz	δ_C	Key HMBC correlations
1		165.8	
2		110.2	
3		168.8	
4		109.2	
5		159.8	
6		127.1	
7	6.10, br s	139.9	C-5, C-9, 6-CH_3
8		131.9	
9	5.96, br s	137.6	C-7, C-11, 8-CH_3
10		132.4	
11	5.86, br s	135.4	C-9, C-13, 10-CH_3
12		133.5	
13	5.24, br s	133.1	C-11, 12-CH_3
14	2.54, m	40.5	
15	3.67, m	72.3	
2-CH_3	2.06, s	10.4	C-1, C-2, C-3
3-OCH_3	3.84, s	60.4	C-3
4-CH_3	2.02, s	12.2	C-3, C-4, C-5
6-CH_3	2.09, d, 1.5	16.8	C-5, C-6, C-7
8-CH_3	2.01, d, 1.5	18.9	C-7, C-8, C-9
10-CH_3	1.95, d, 1.5	19.1	C-9, C-10, C-11
12-CH_3	1.82, d, 1.5	17.6	C-11, C-12, C-13
14-CH_3	1.05, d, 6.8	16.9	C-13, C-14, C-15
15-CH_3	1.19, d, 6.5	20.9	C-14, C-15

Table S15. NMR data of a diastereomeric pair of policosidin F (**10**) in $CDCl_3$.

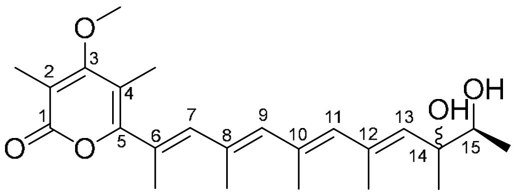

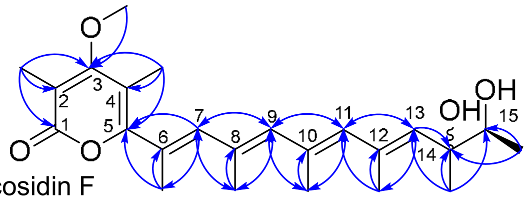

policosidin F

Position	δ_H, multi., *J* in Hz	δ_C	Key HMBC correlations
1		165.8	
2		110.2	
3		168.8	
4		109.2	
5		159.7[a]/159.7[b]	
6		127.2[a]/127.3[b]	
7	6.09, br s	139.8[a]/139.8[b]	C-5, C-9, 6-CH_3, 8-CH_3
8		132.2[a]/132.3[b]	
9	5.94, br s	137.1[a]/137.2[b]	C-7, C-11, 8-CH_3, 10-CH_3
10		132.8[a]/132.9[b]	
11	5.82[a]/5.83[b], br s	135.8[a]/136.9[b]	C-9, C-13, 10-CH_3
12		136.1[a]/136.1[b]	
13	5.38[a]/5.39[a], br s	131.7[a]/133.6[b]	C-11, C-14, 12-CH_3
14		76.1[a]/76.3[b]	
15	3.71[a]/3.85[b], q, 6.0	73.7[a]/75.0[b]	
2-CH_3	2.05, s	10.4	C-1, C-2, C-3
3-OCH_3	3.83, s	60.3	C-3
4-CH_3	2.01, s	12.2	C-3, C-4, C-5
6-CH_3	2.08, br s	16.8	C-5, C-6, C-7
8-CH_3	2.00, br s	18.9	C-7, C-8, C-9
10-CH_3	1.93[a]/1.93[b], br s	19.0[a]/19.1[b]	C-9, C-10, C-11
12-CH_3	2.01[a]/2.02[b], br s	18.4[a]/18.5[b]	C-11, C-12, C-13
14-CH_3	1.32[a]/1.40[b], s	22.8[a]/25.8[b]	C-13, C-14, C-15
15-CH_3	1.18[a]/1.23[b], d, 6.0	17.3[a]/18.1[b]	C-14, C-15

[a/b]: The signals correspond to two different diastereomers of policosidin F (**10**).

Table S16. NMR data of policosidin G (**11**) in $CDCl_3$.

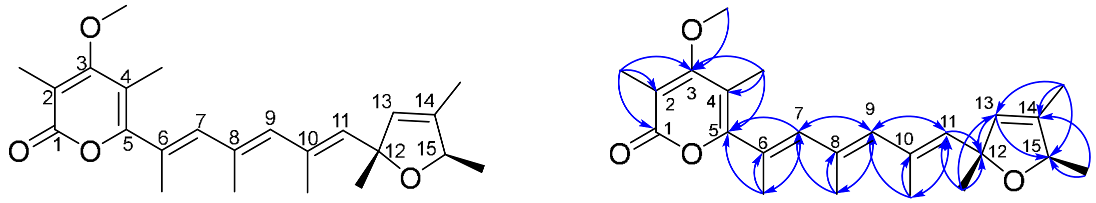

policosidin G

Position	δ_H, multi., *J* in Hz	δ_C	Key HMBC correlations
1		165.8	
2		110.2	
3		168.8	
4		109.1	
5		159.8	
6		127.4	
7	6.07, br s	139.7	C-5, C-9, 6-CH_3
8		131.8	
9	5.87, br s	137.2	C-7, C-11, 8-CH_3
10		132.8	
11	5.53, br s	136.2	C-9, C-12, 10-CH_3
12		88.0	
13	5.46, br s	127.2	C-12, C-15
14		138.6	
15	4.71, q, 6.0	82.9	
2-CH_3	2.06, s	10.4	C-1, C-2, C-3
3-OCH_3	3.83, s	60.4	C-3
4-CH_3	2.01, s	12.2	C-3, C-4, C-5
6-CH_3	2.07, d, 1.5	16.8	C-5, C-6, C-7
8-CH_3	1.96, d, 1.5	18.6	C-7, C-8, C-9
10-CH_3	1.87, d, 1.5	17.7	C-9, C-10, C-11
12-CH_3	1.46, s	30.4	C-11, C-12, C-13
14-CH_3	1.70, br s	12.3	C-13, C-14, C-15
15-CH_3	1.30, d, 6.0	21.6	C-14, C-15

The signals of policosidin G (**11**) were picked from the spectra of a diastereomeric mixture with poloncosidin A (**5**).

Supplementary Figures

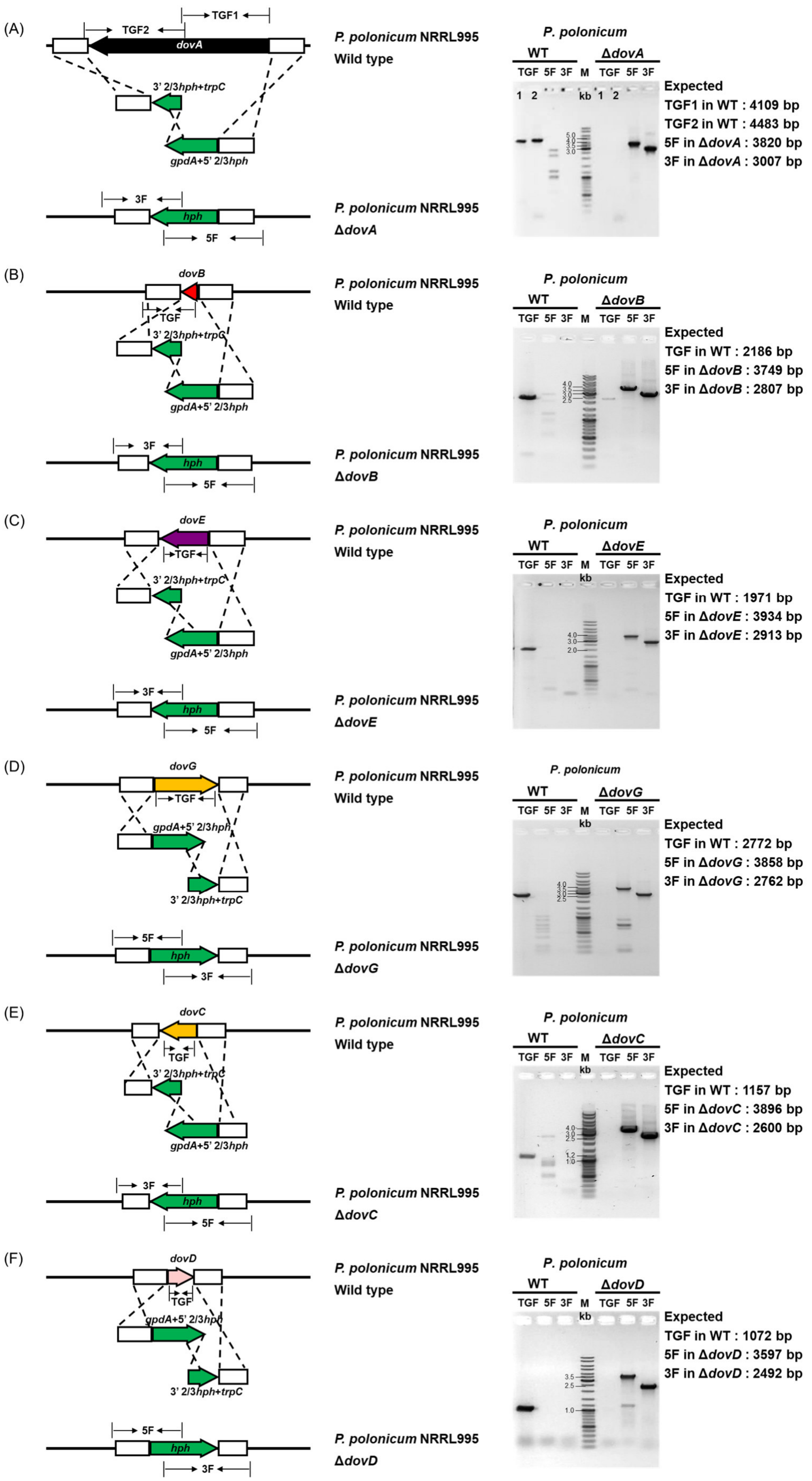

Figure S1. Target gene deletion and PCR verification for *P. polonicum* strains.

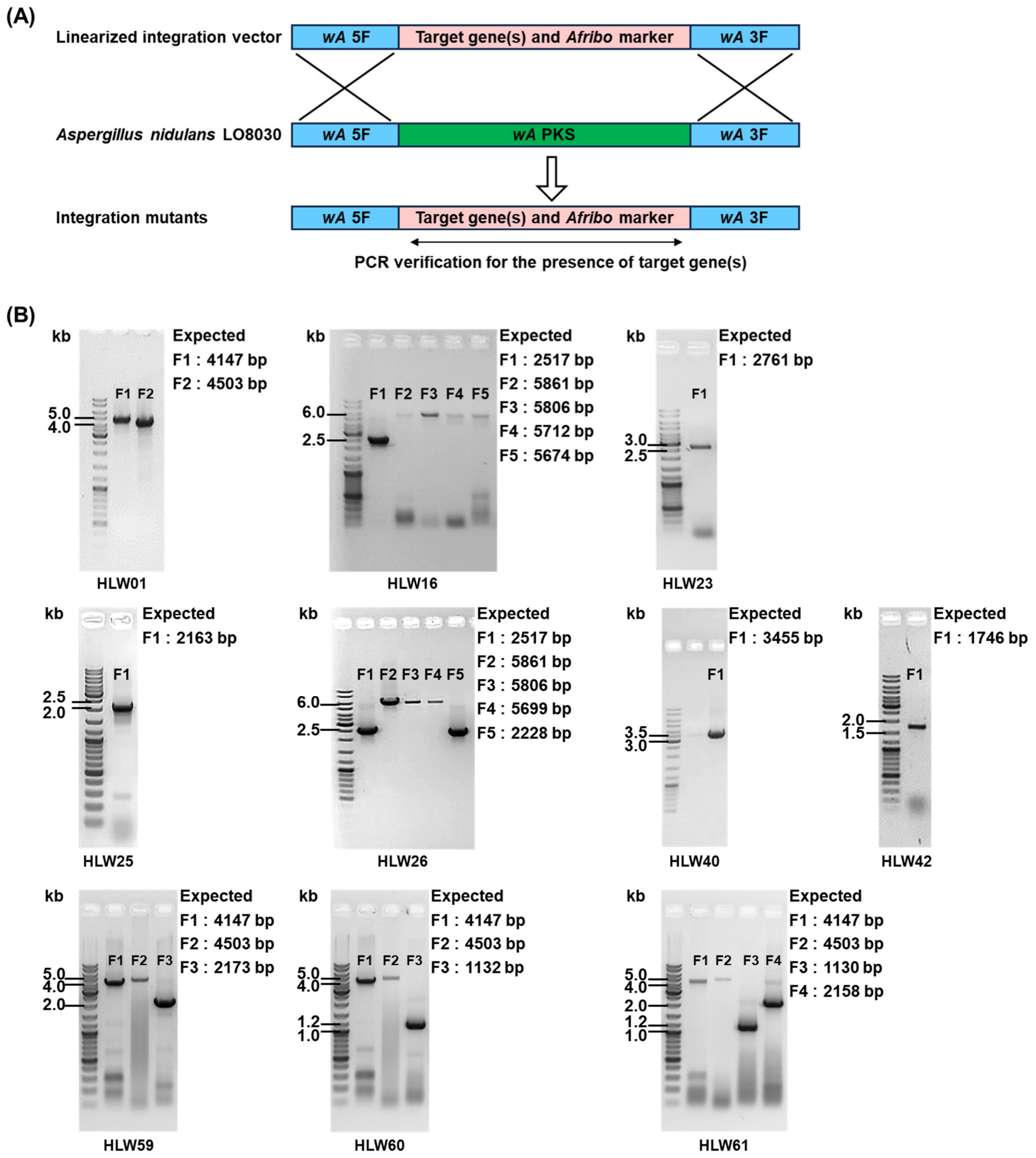

Figure S2. Heterologous expression and PCR verification for *A. nidulans* strains.
(A) Schematic representation of gene integration into the *wA*-PKS locus of *A. nidulans* LO8030. (B) PCR verifications for the presence of the target gene(s) were performed with genomic DNA of the *A. nidulans* transformants using primers listed in Table S6.

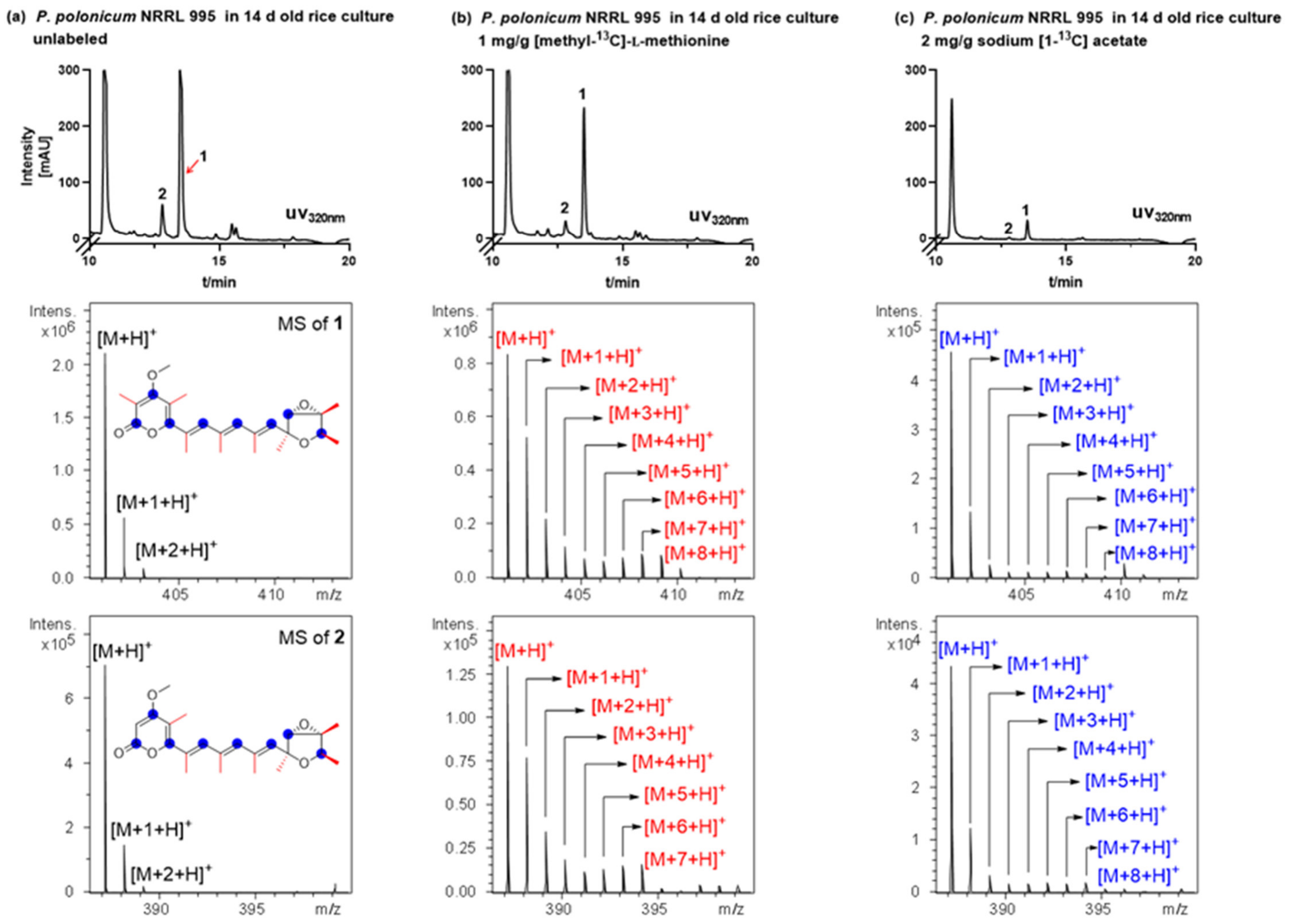

Figure S3. MS analysis of ^{13}C-labeled precursors supplied for *P. polonicum* NRRL 995.

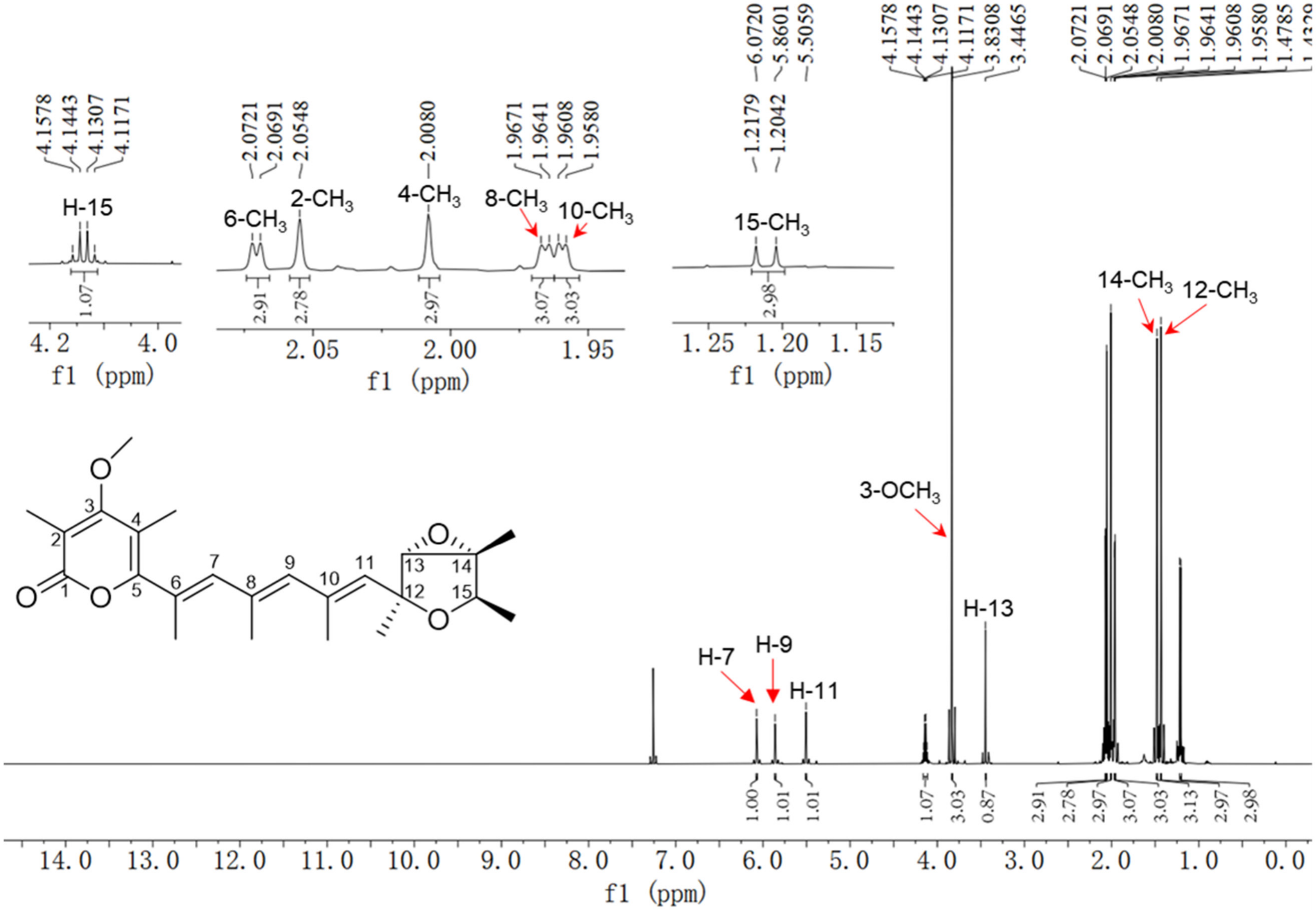

Figure S4. ^{1}H NMR spectrum of deoxyverrucosidin (**1**) in $CDCl_3$ (500 MHz).

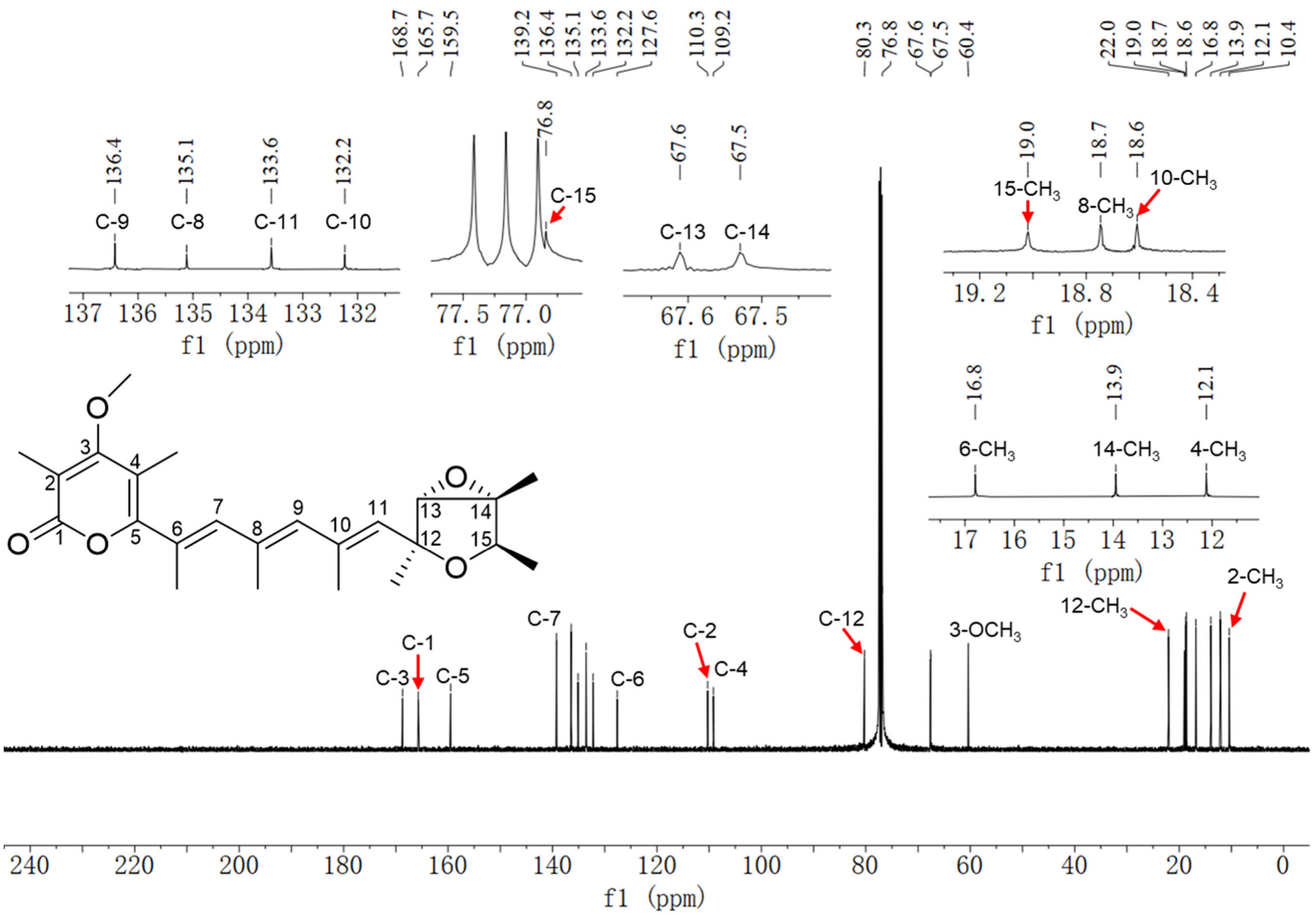

Figure S5. ^{13}C NMR spectrum of deoxyverrucosidin (**1**) in $CDCl_3$ (125 MHz).

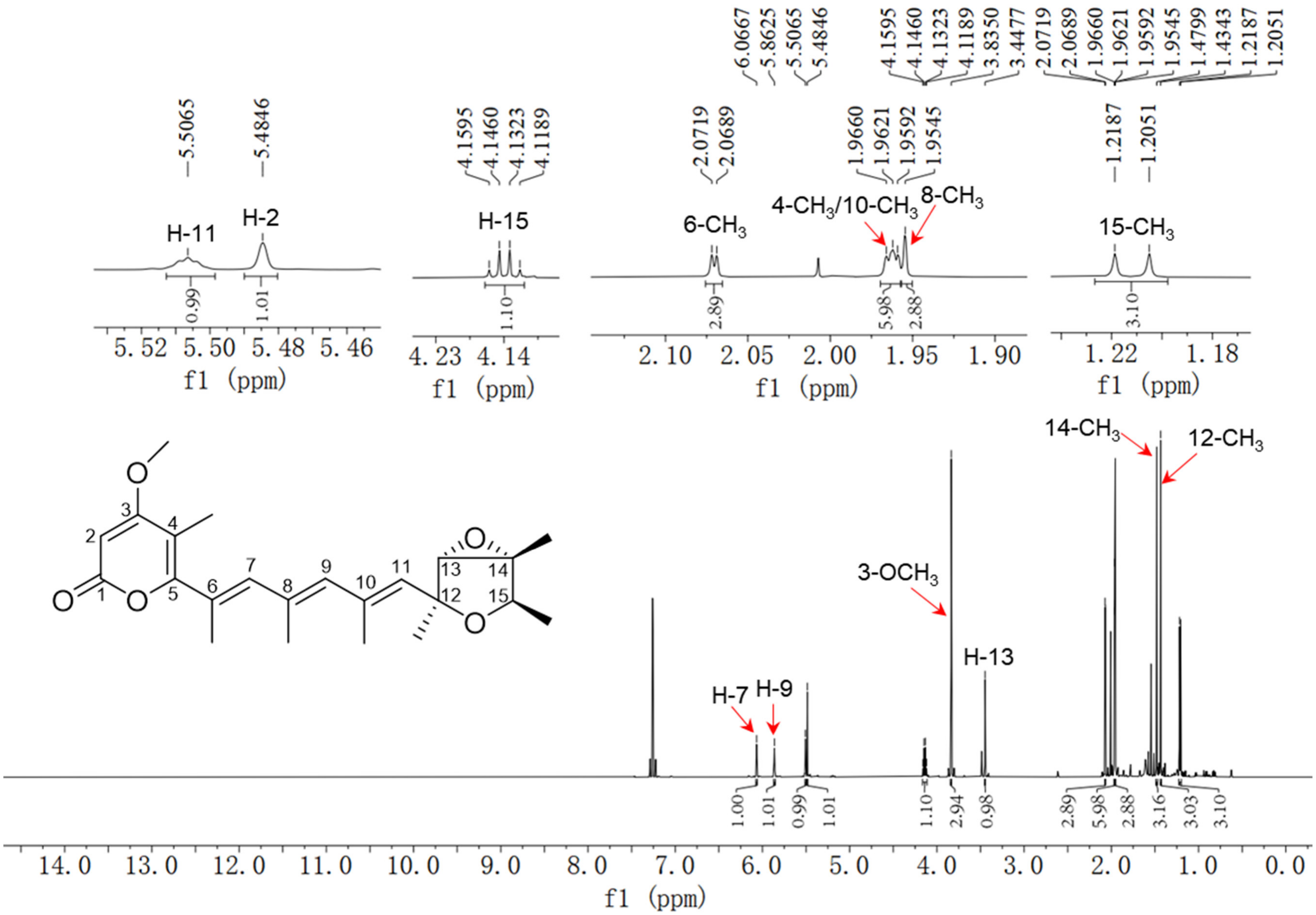

Figure S6. ^{1}H NMR spectrum of nordeoxyverrucosidin (**2**) in $CDCl_3$ (500 MHz).

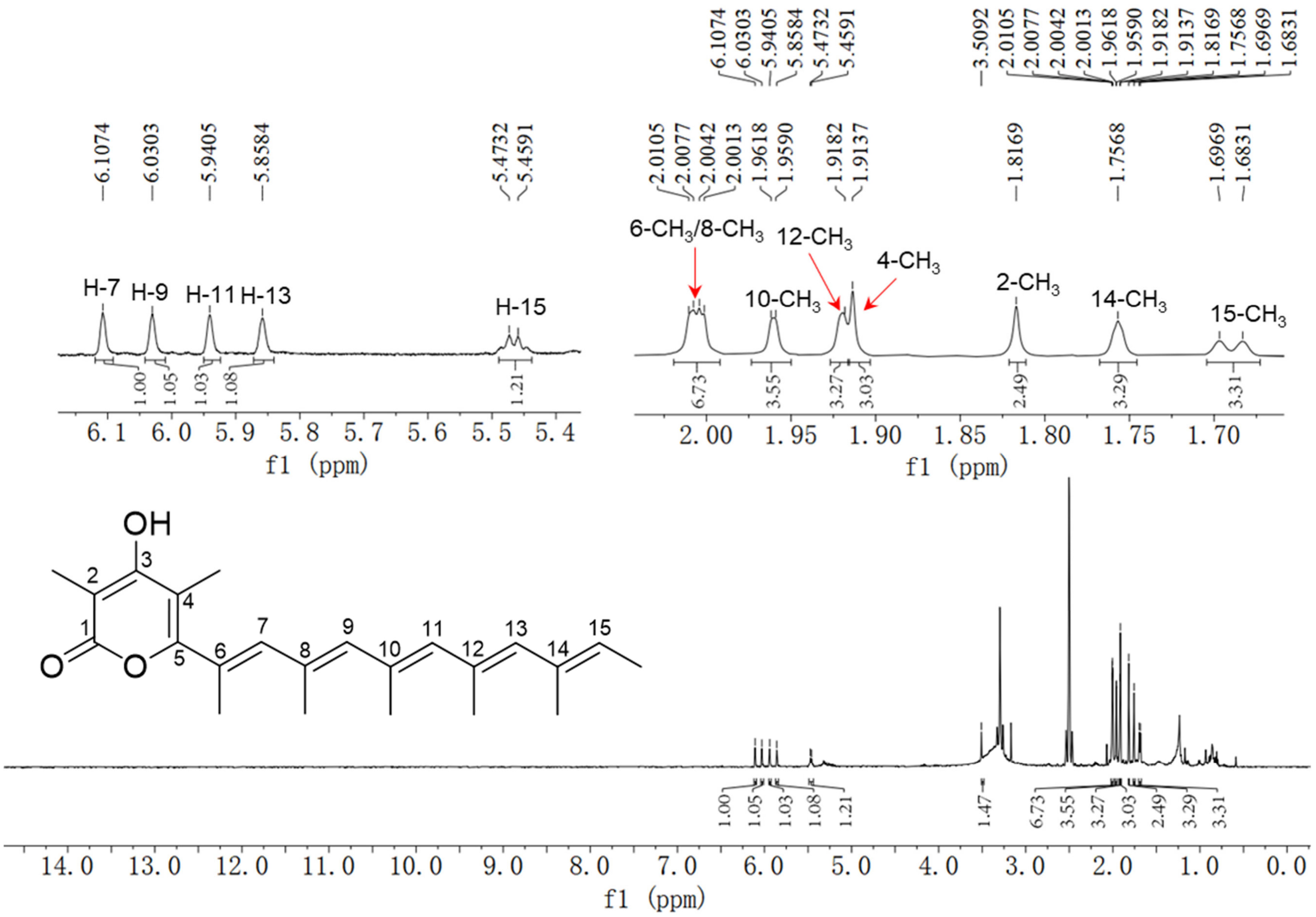

Figure S7. ^{1}H NMR spectrum of policosidin A (**3**) in DMSO-d_6 (500 MHz).

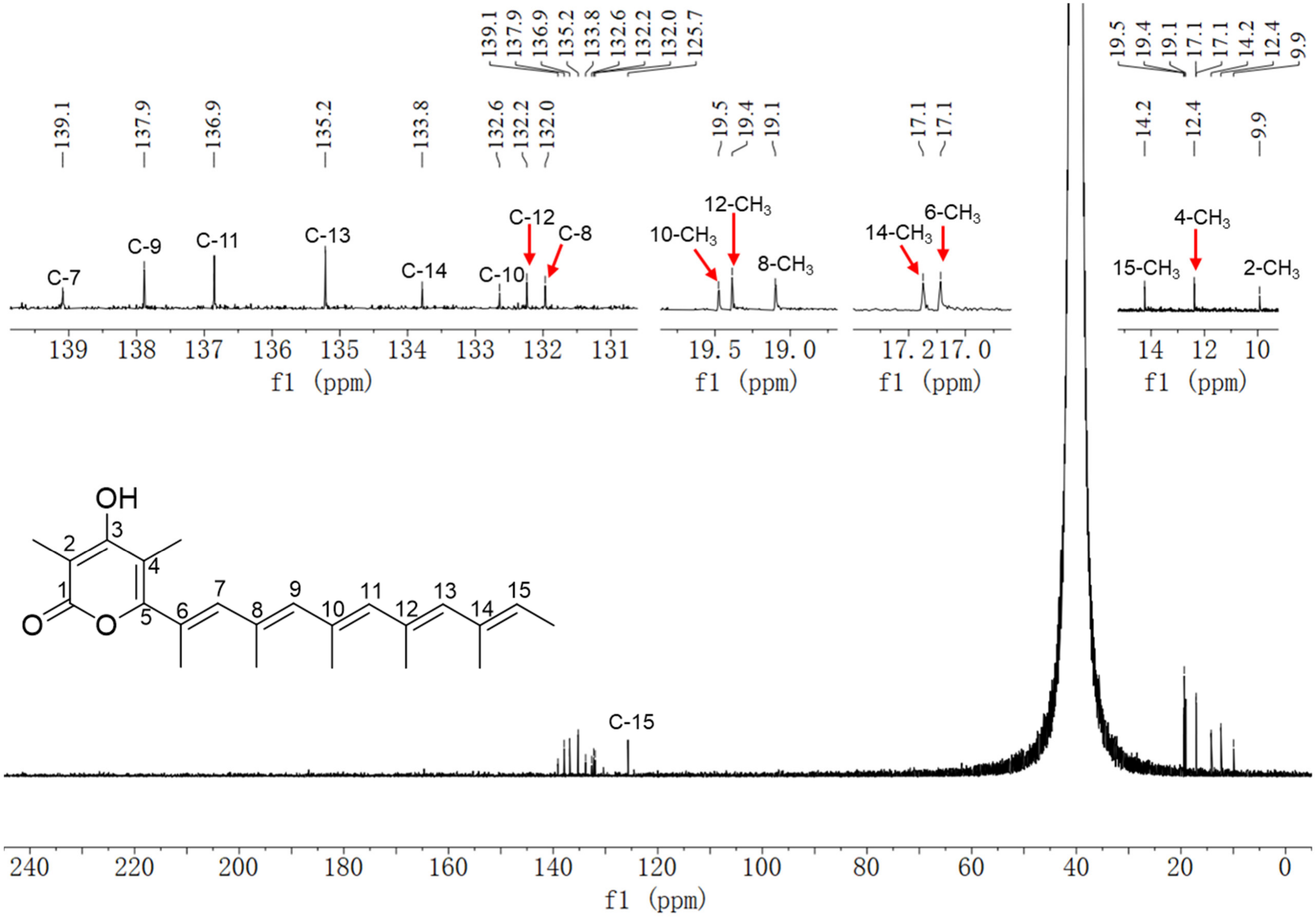

Figure S8. ^{13}C NMR spectrum of policosidin A (**3**) in DMSO-d_6 (125 MHz).

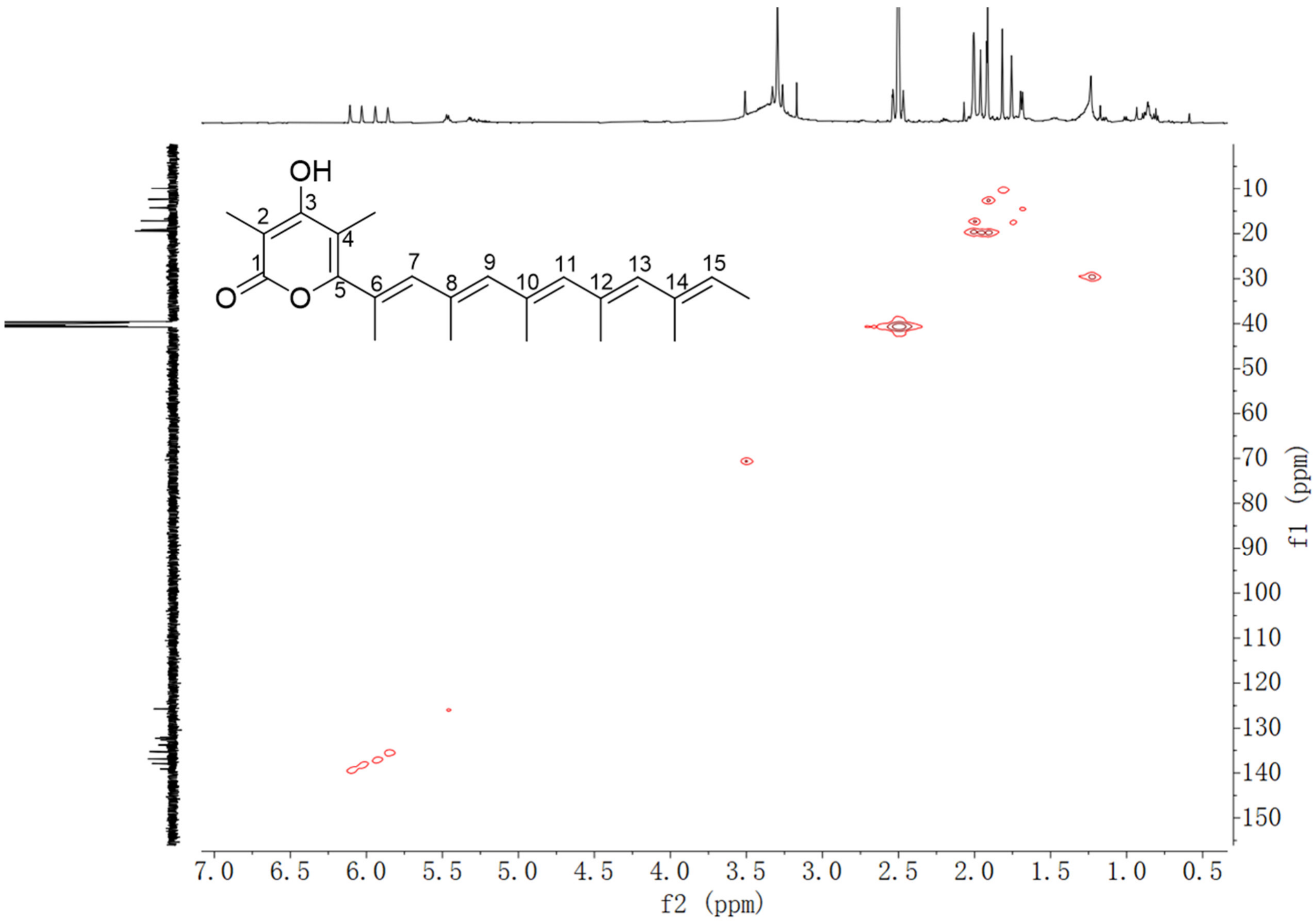

Figure S9. HSQC spectrum of policosidin A (**3**) in DMSO-d_6.

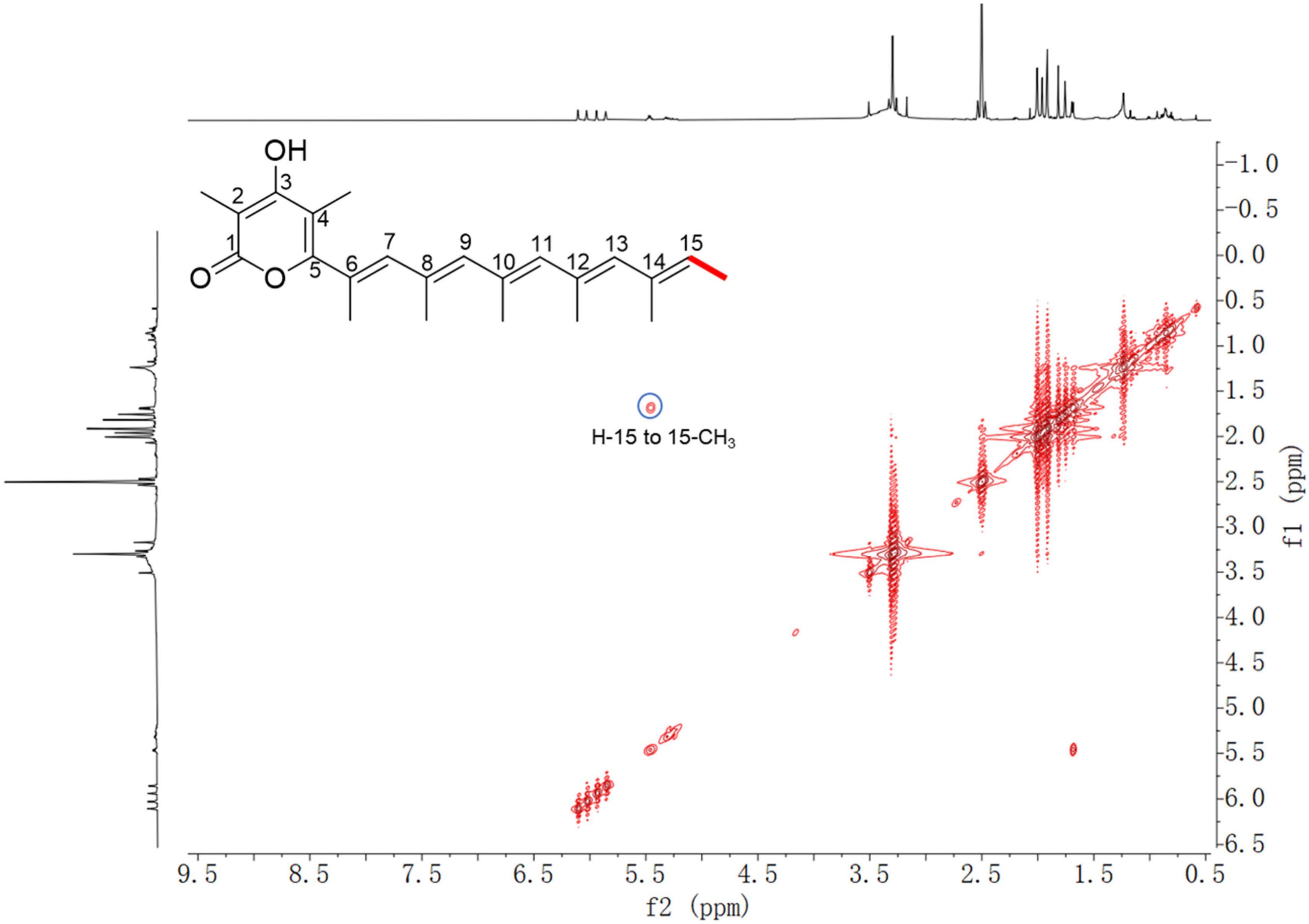

Figure S10. DQF-COSY spectrum of policosidin A (**3**) in DMSO-*d*6.

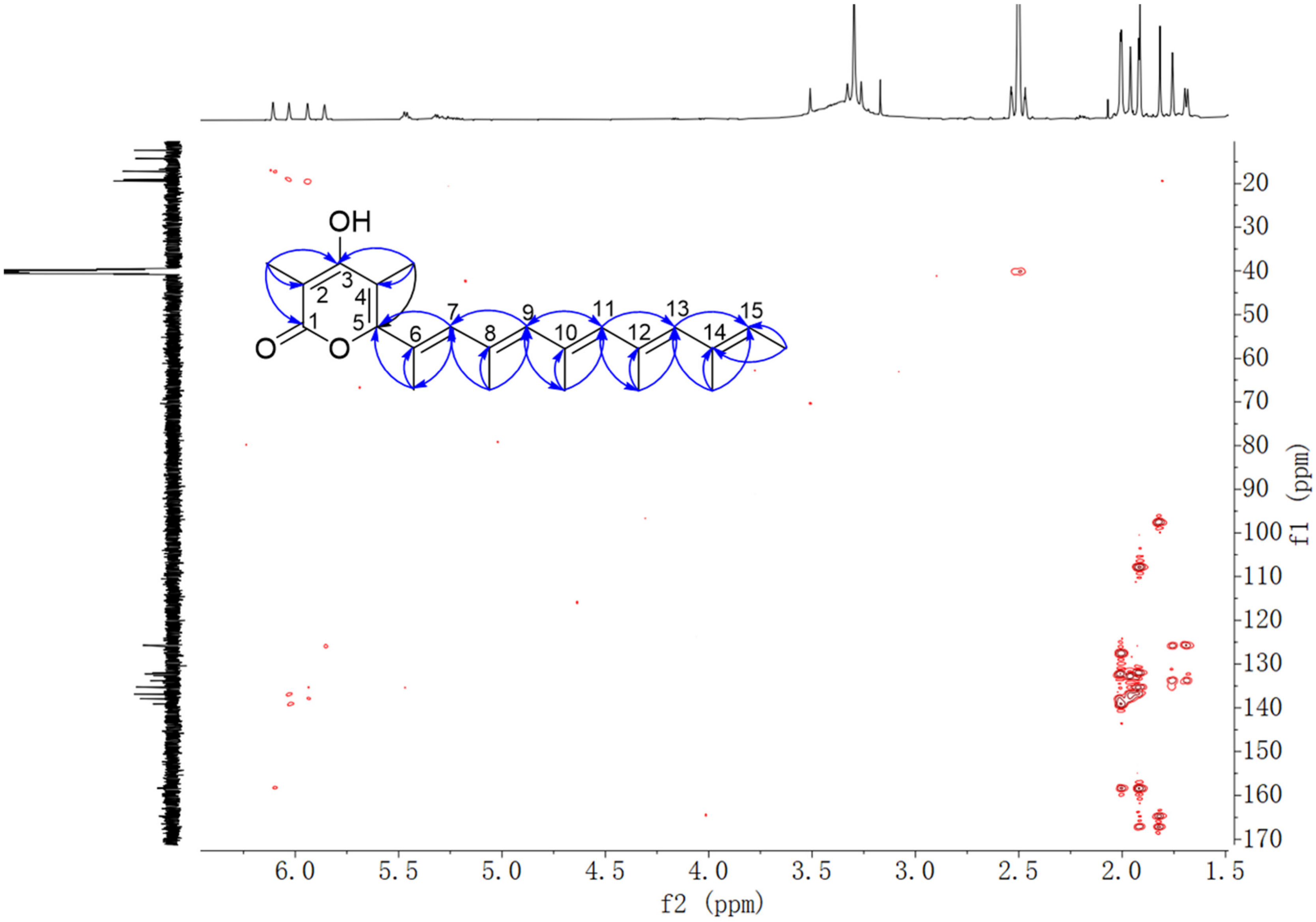

Figure S11. HMBC spectrum of policosidin A (**3**) in DMSO-d_6.

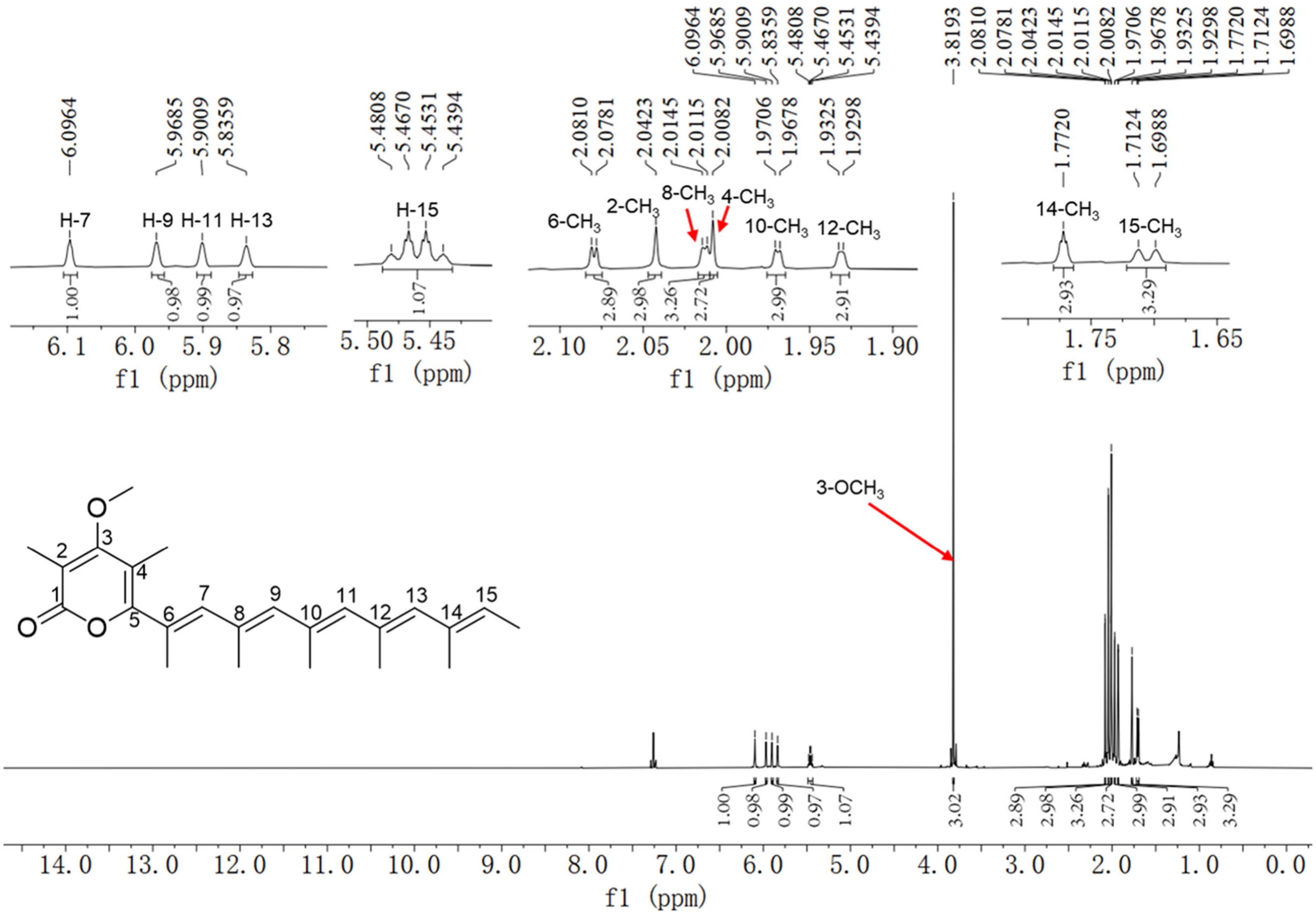

Figure S12. ^{1}H NMR spectrum of policosidin B (**4**) in $CDCl_3$ (500 MHz).

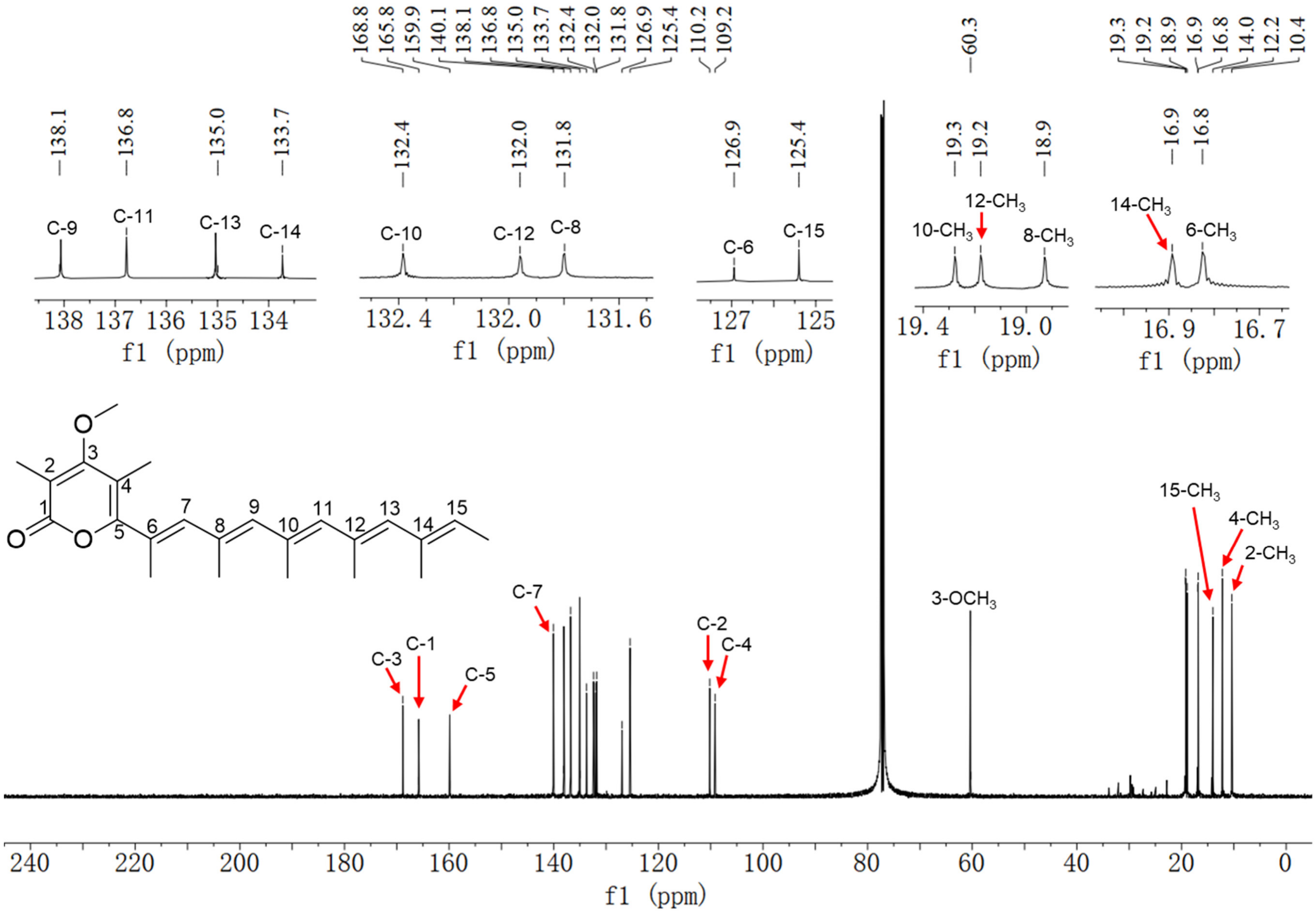

Figure S13. ^{13}C NMR spectrum of policosidin B (**4**) in $CDCl_3$ (125 MHz).

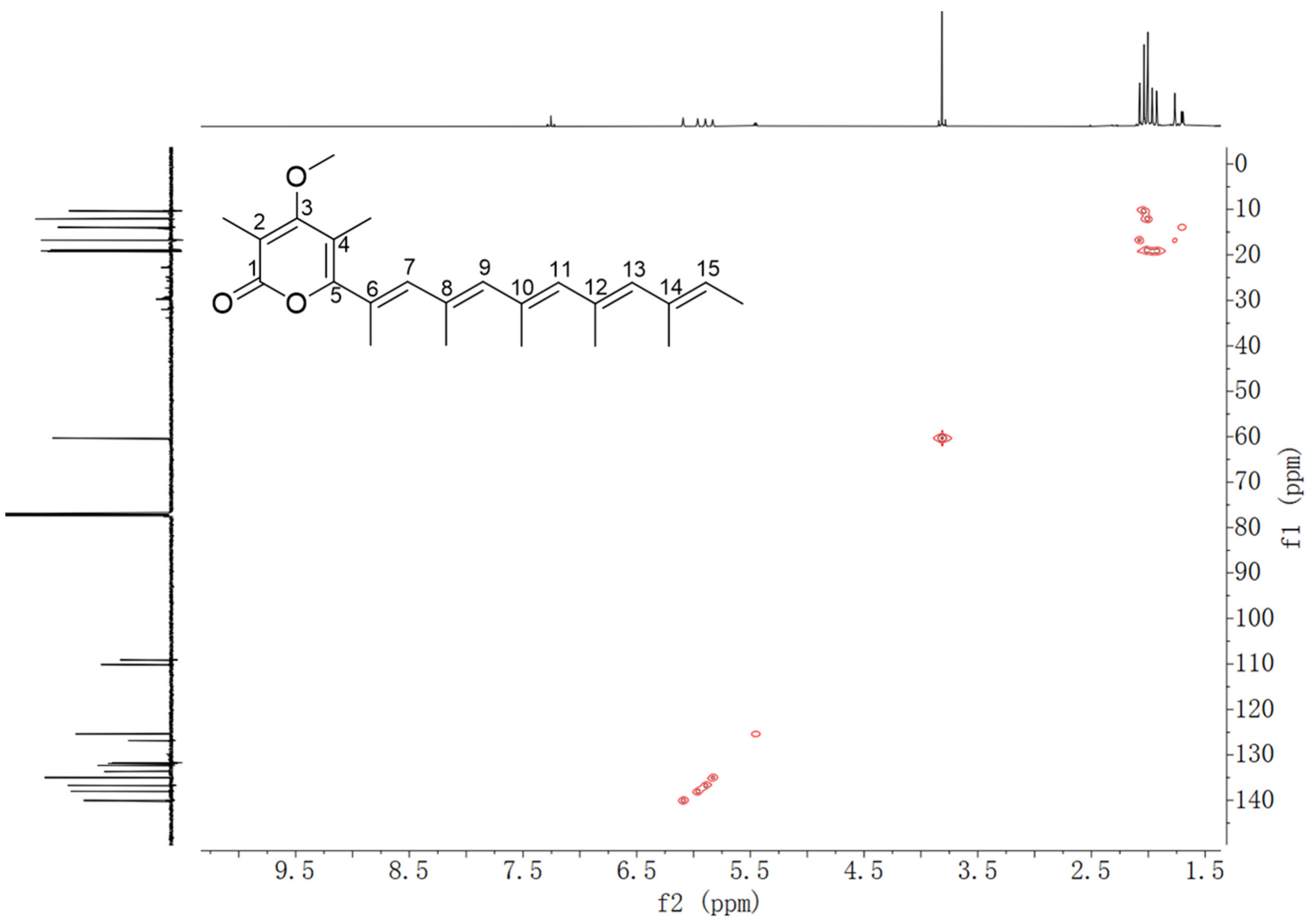

Figure S14. HSQC spectrum of policosidin B (**4**) in $CDCl_3$.

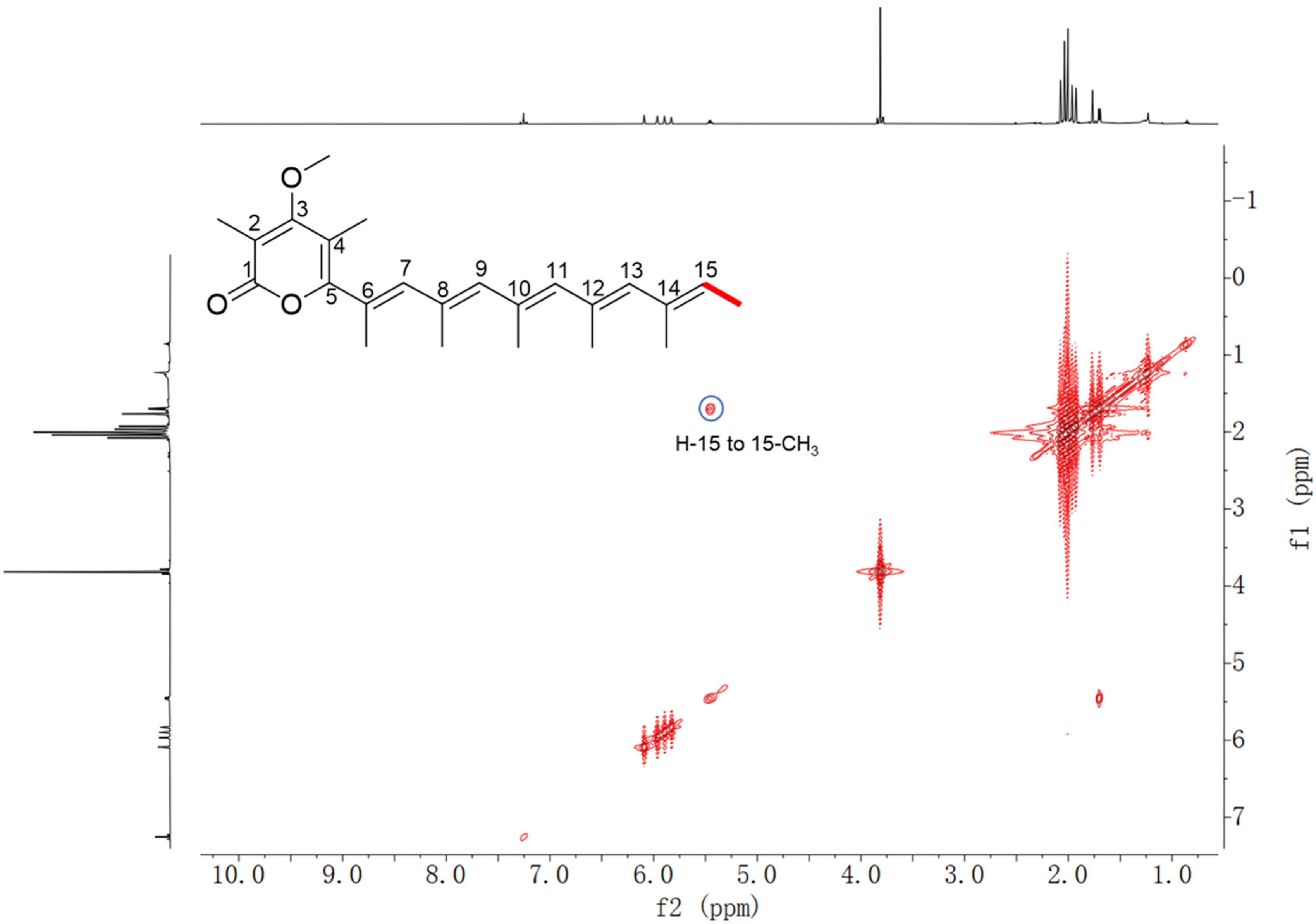

Figure S15. DQF-COSY spectrum of policosidin B (**4**) in $CDCl_3$.

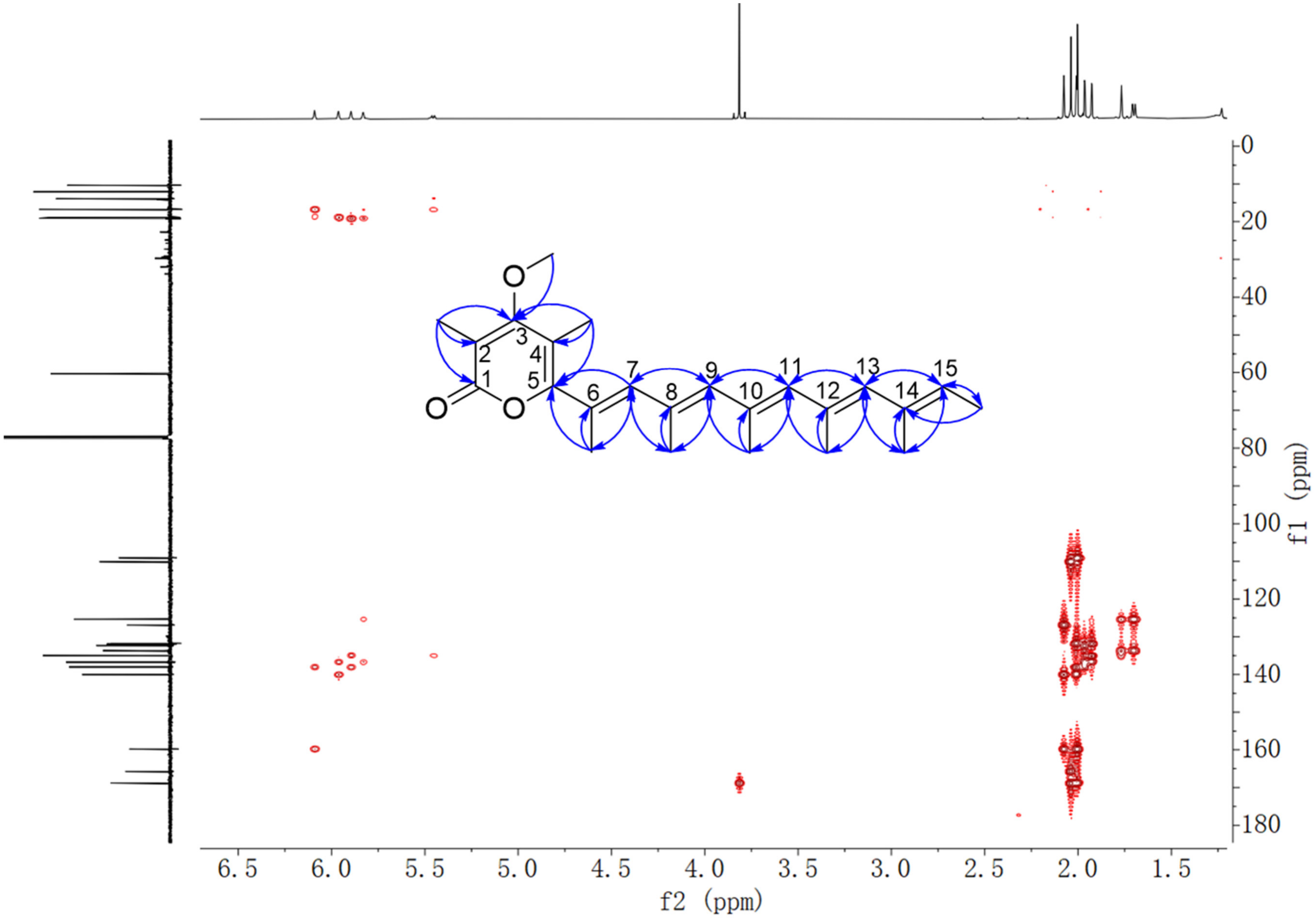

Figure S16. HMBC spectrum of policosidin B (**4**) in $CDCl_3$.

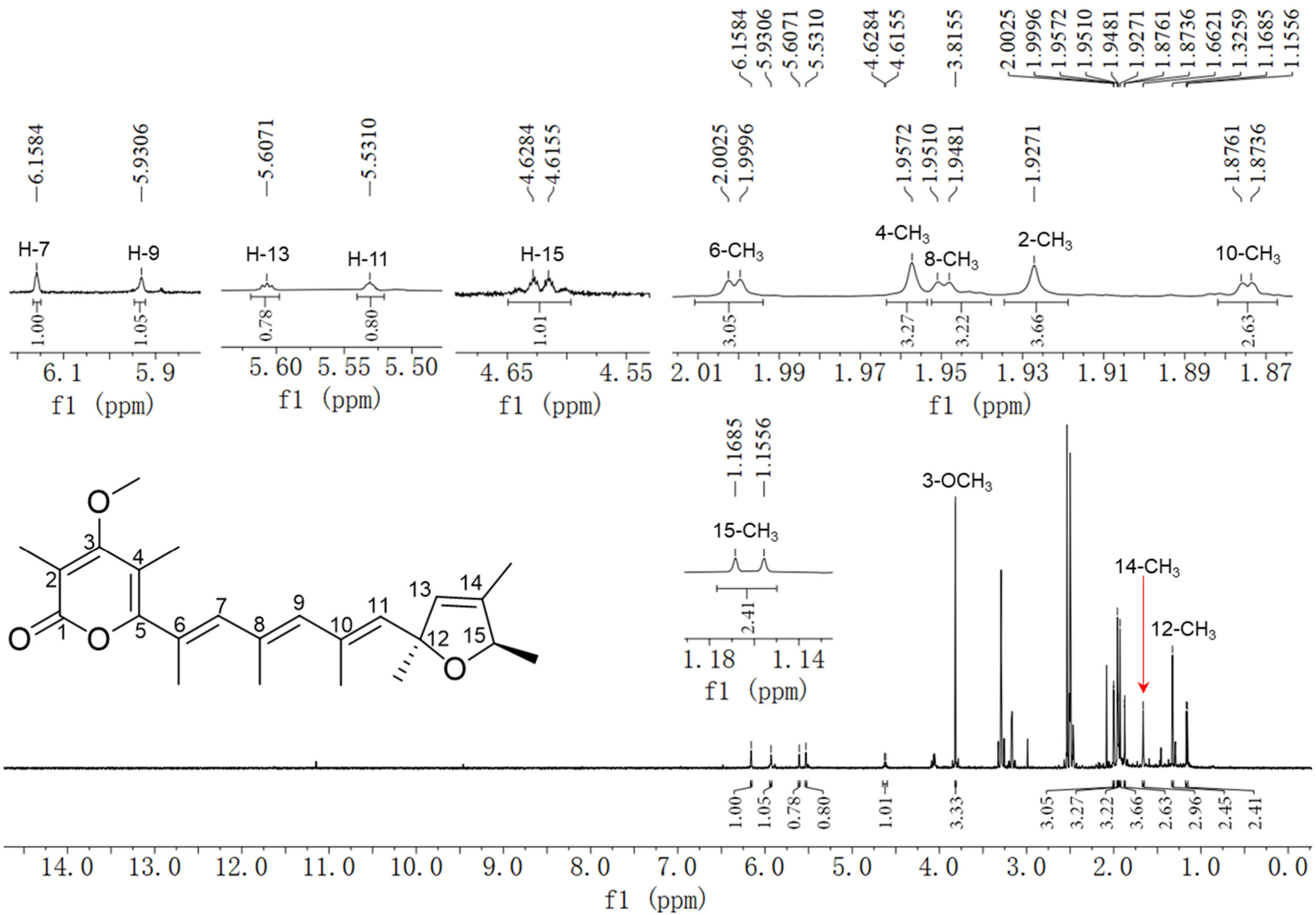

Figure S17. ^{1}H NMR spectrum of poloncosidin A (**5**) in DMSO-d_6 (500 MHz).

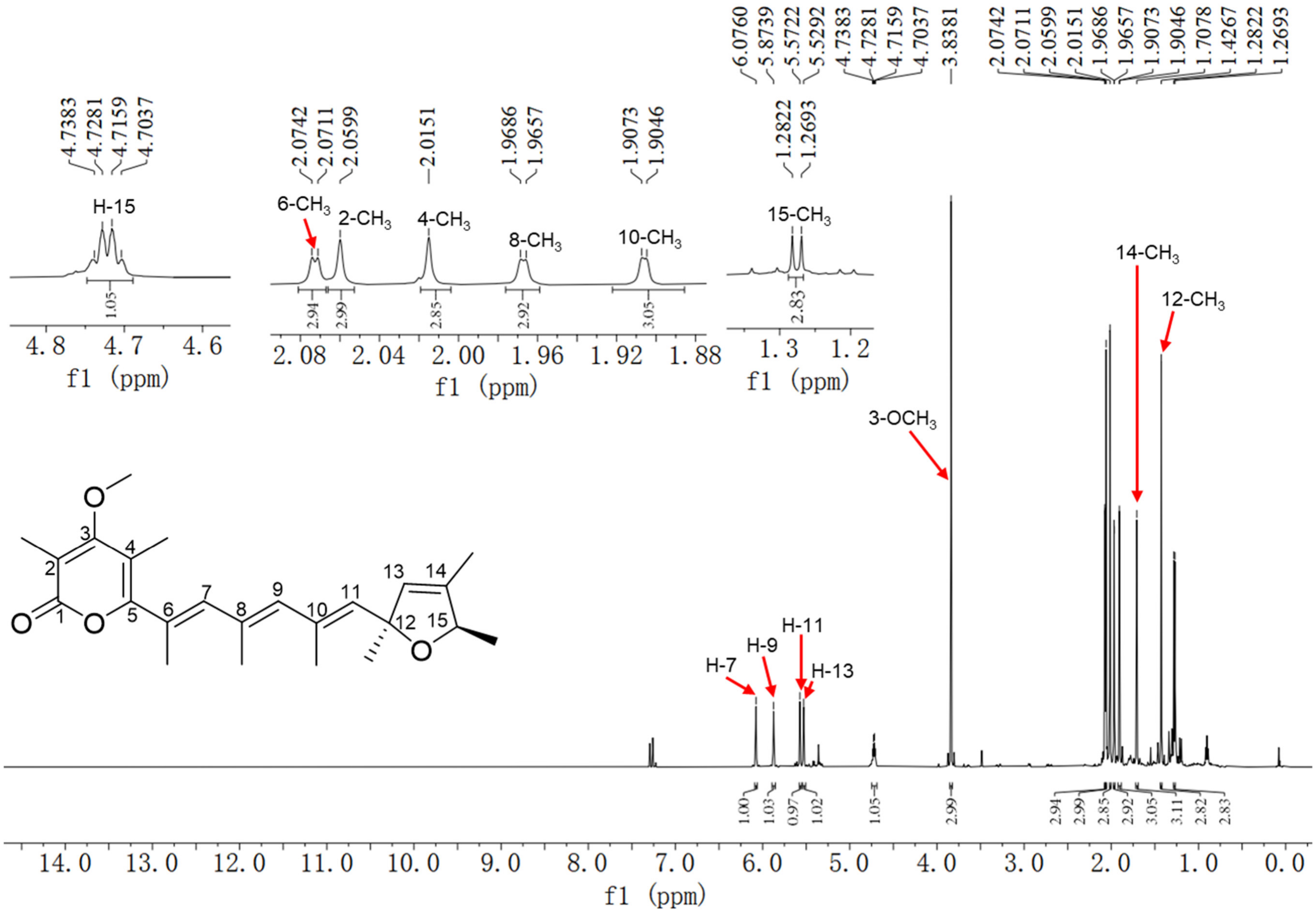

Figure S18. ^{1}H NMR spectrum of poloncosidin A (**5**) in $CDCl_3$ (500 MHz).

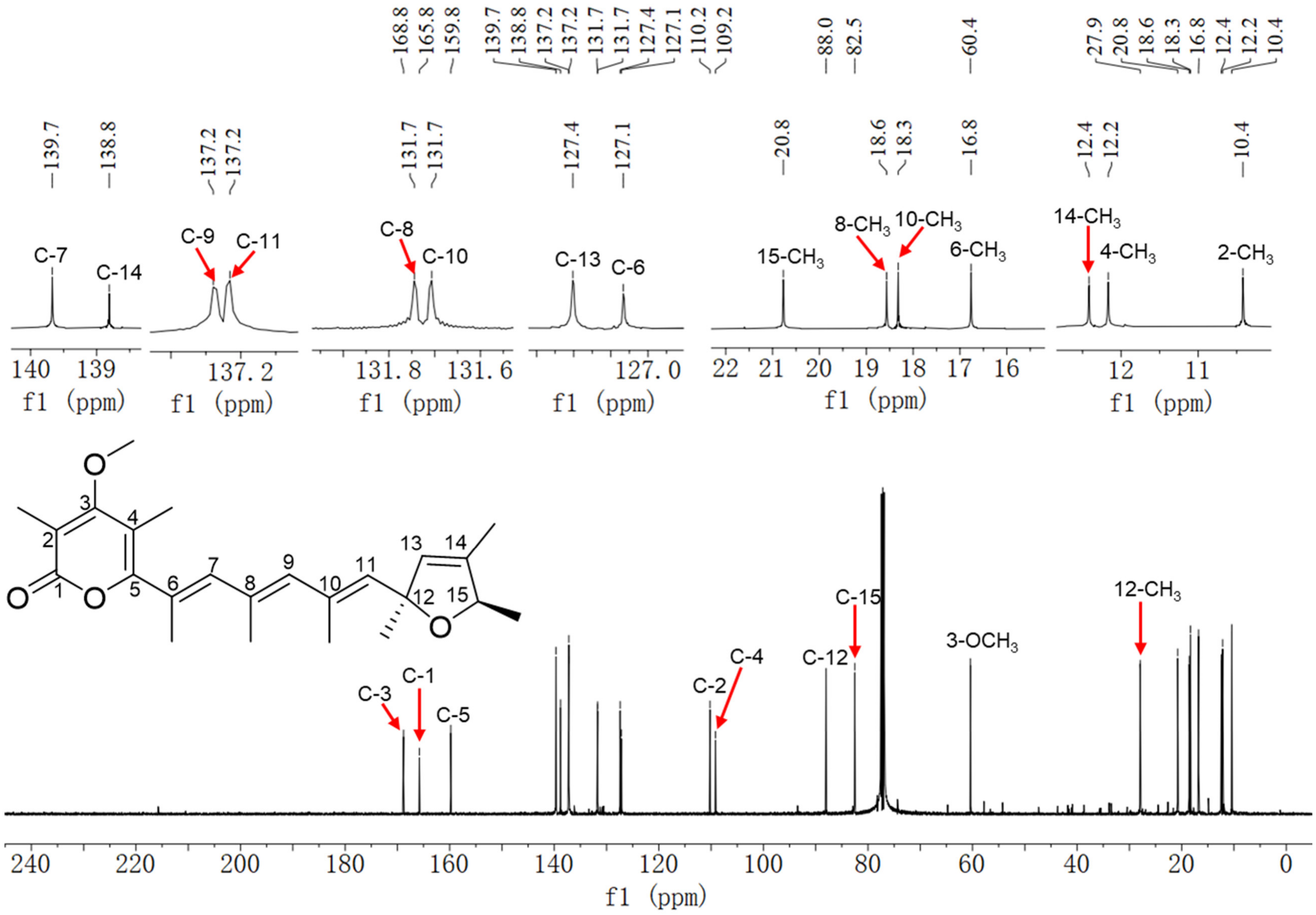

Figure S19. ^{13}C NMR spectrum of poloncosidin A (**5**) in $CDCl_3$ (125 MHz).

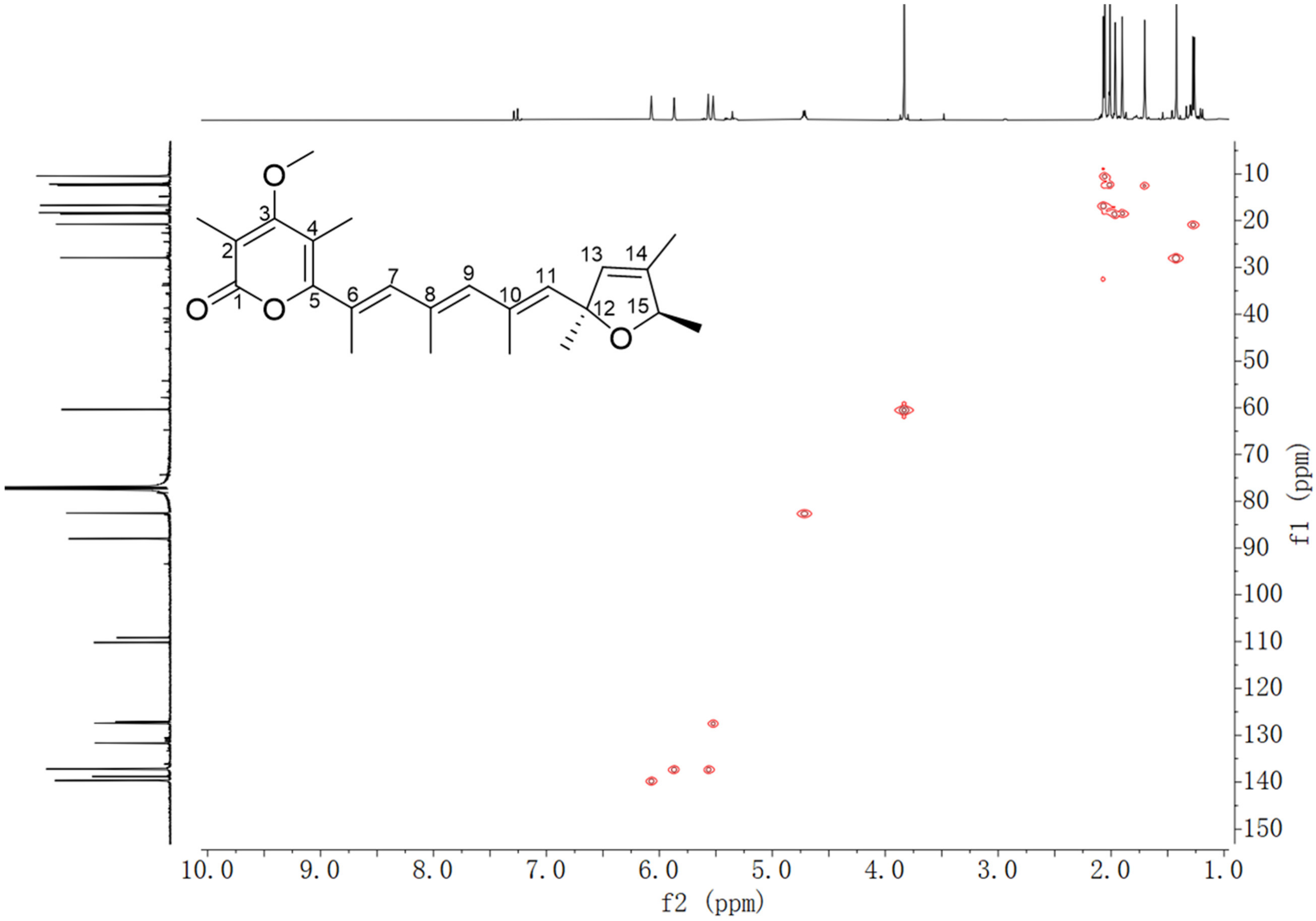

Figure S20. HSQC spectrum of poloncosidin A (**5**) in $CDCl_3$.

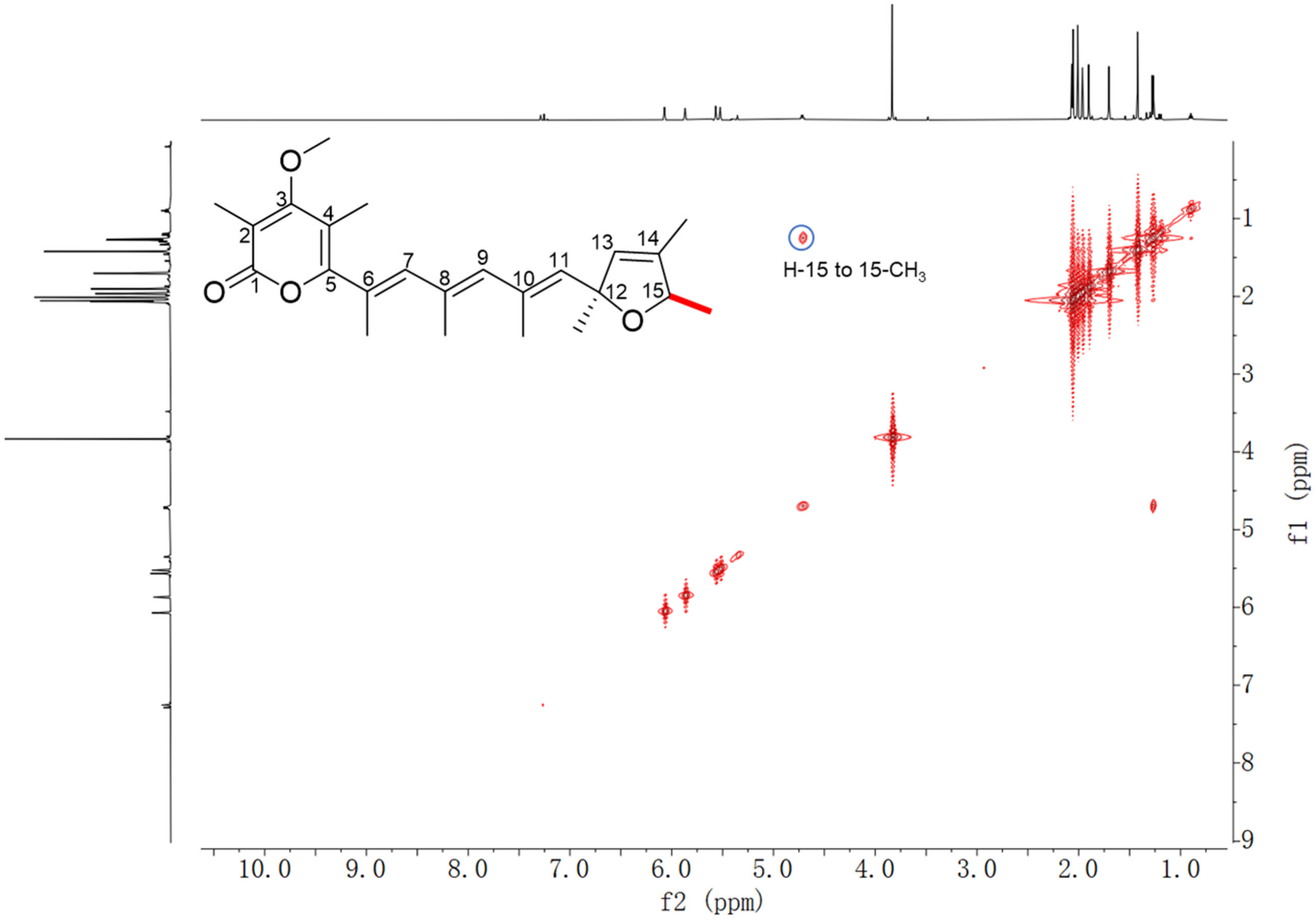

Figure S21. DQF-COSY spectrum of poloncosidin A (**5**) in $CDCl_3$.

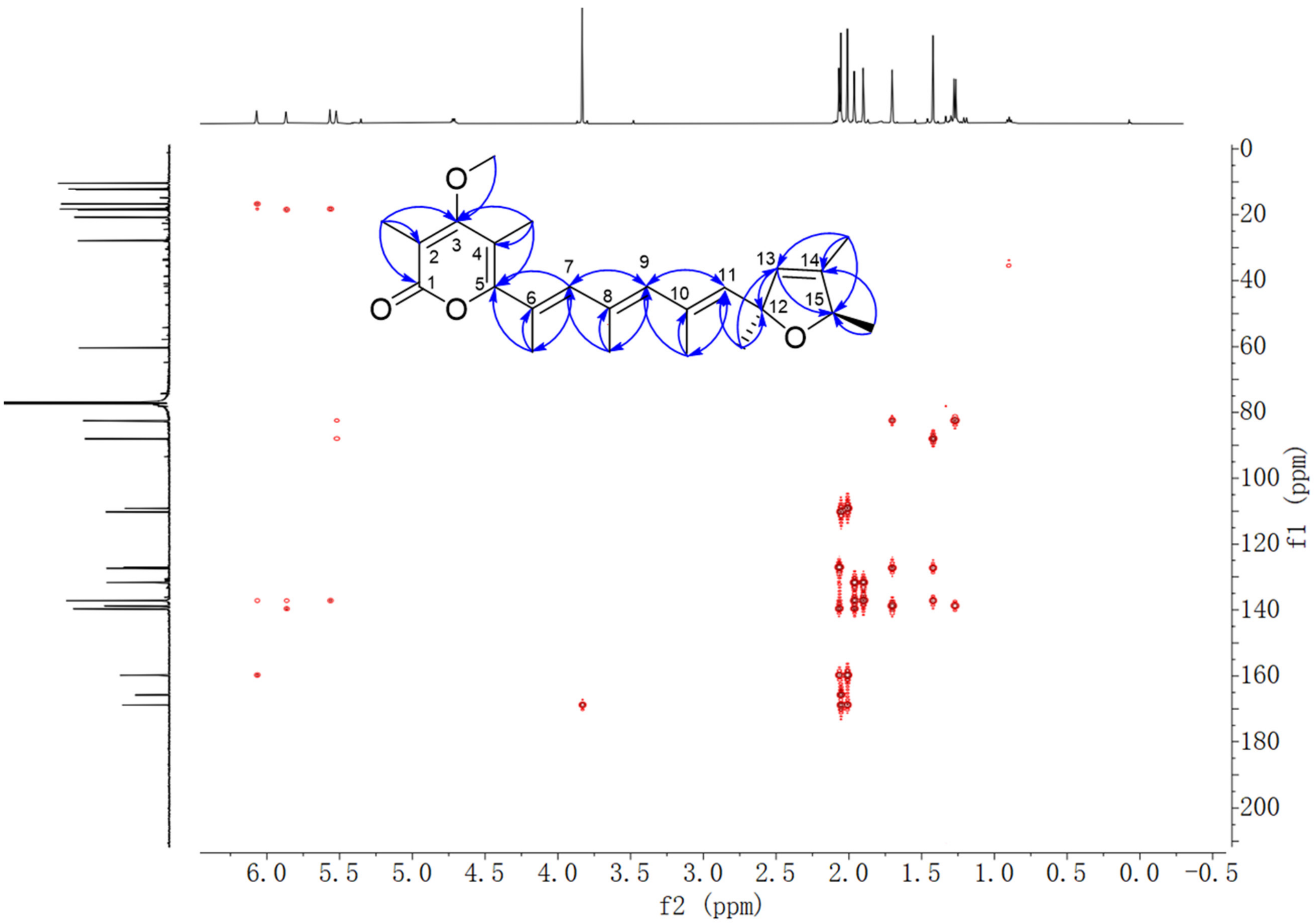

Figure S22. HMBC spectrum of poloncosidin A (**5**) in $CDCl_3$.

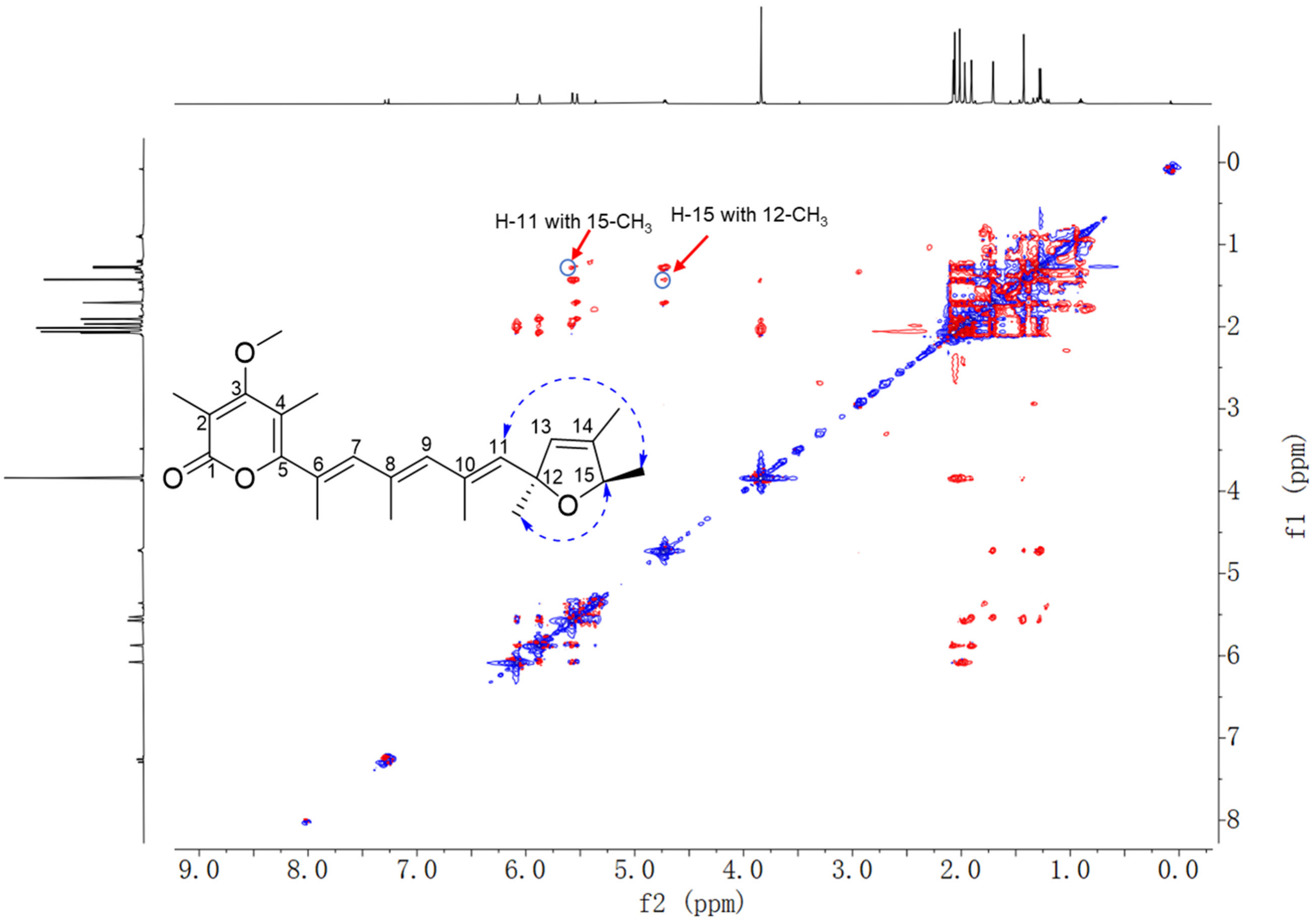

Figure S23. NOESY spectrum of poloncosidin A (**5**) in $CDCl_3$.

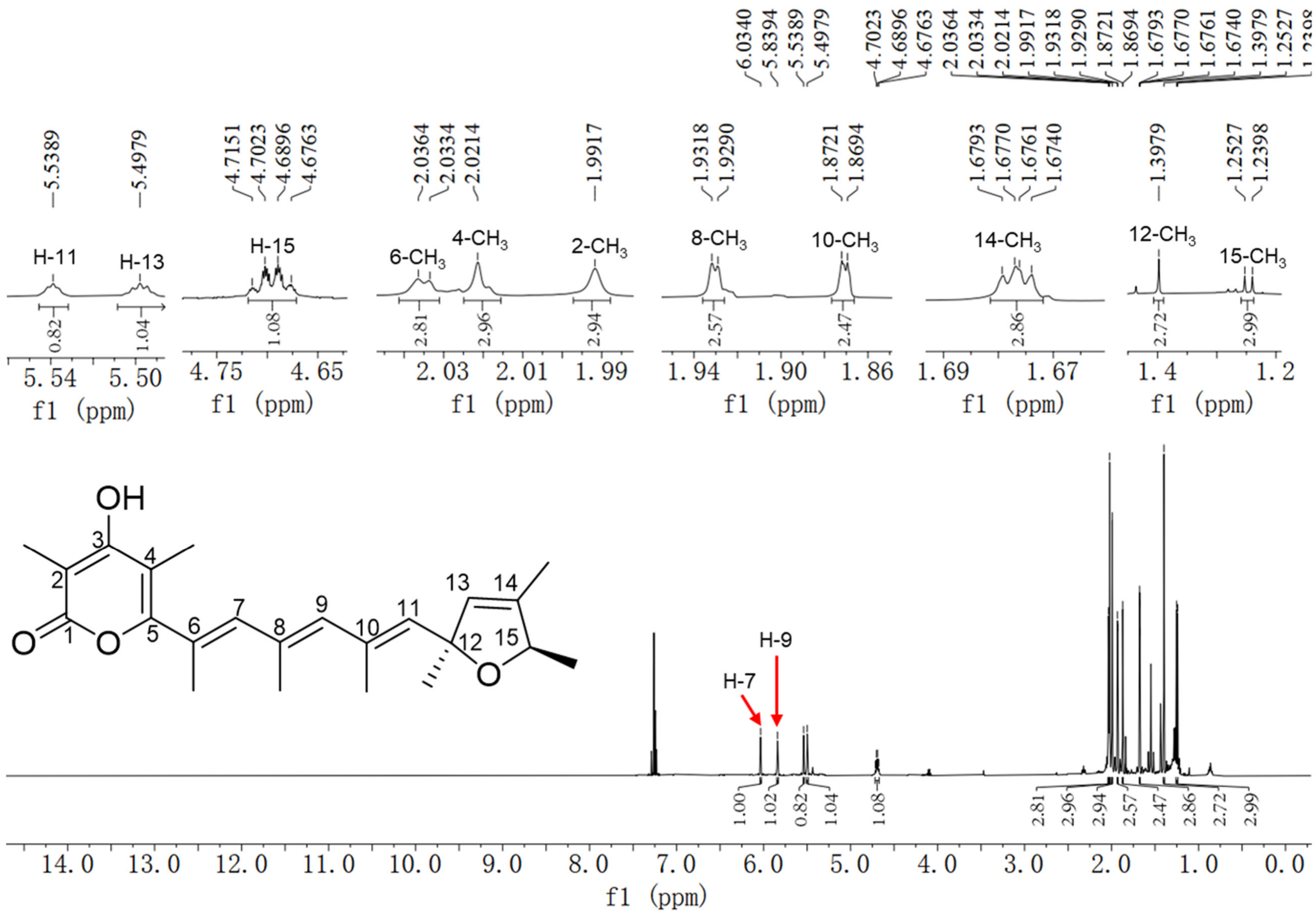

Figure S24. ^{1}H NMR spectrum of policosidin C (**6**) in $CDCl_3$ (500 MHz).

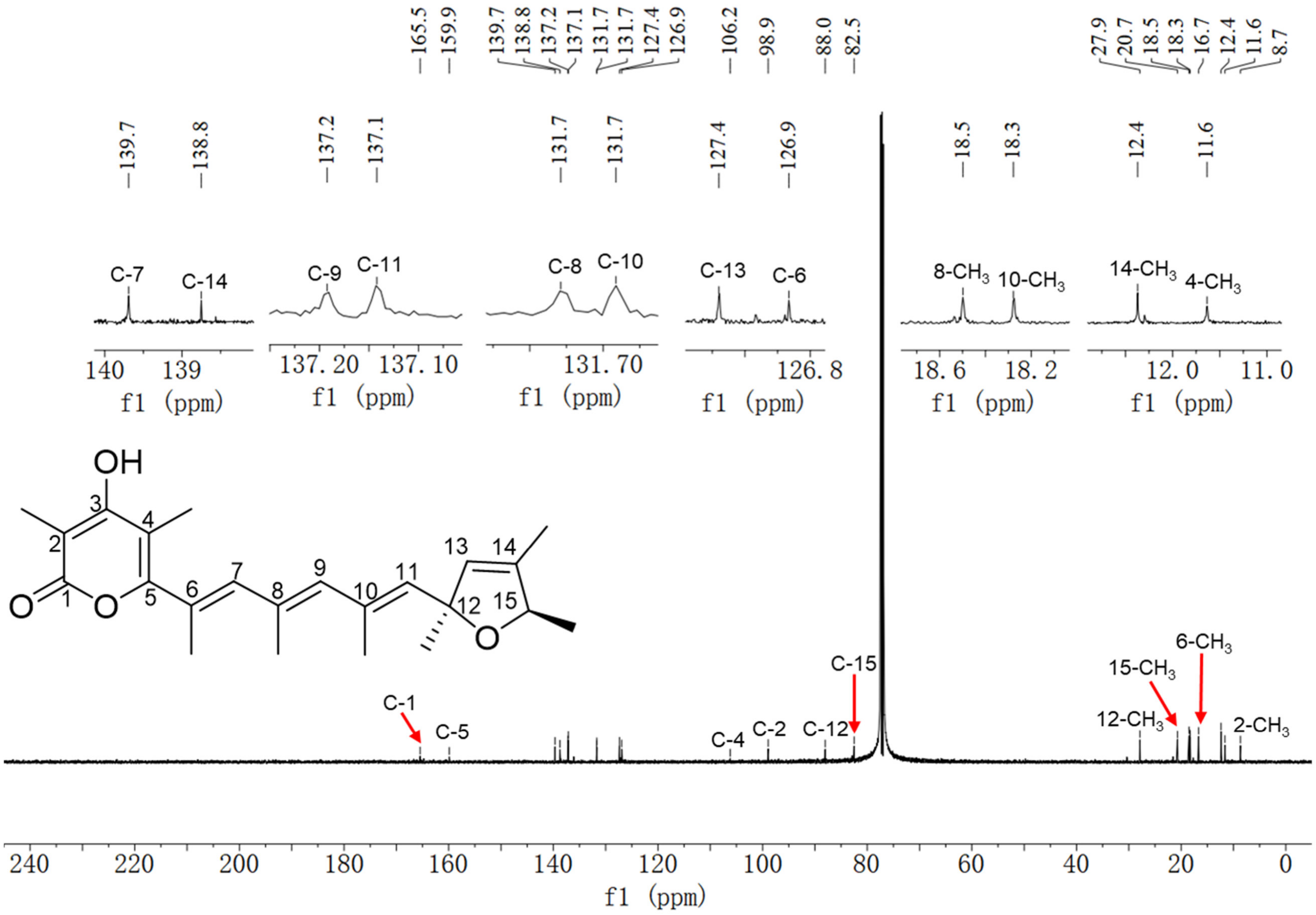

Figure S25. ^{13}C NMR spectrum of policosidin C (**6**) in $CDCl_3$ (125 MHz).

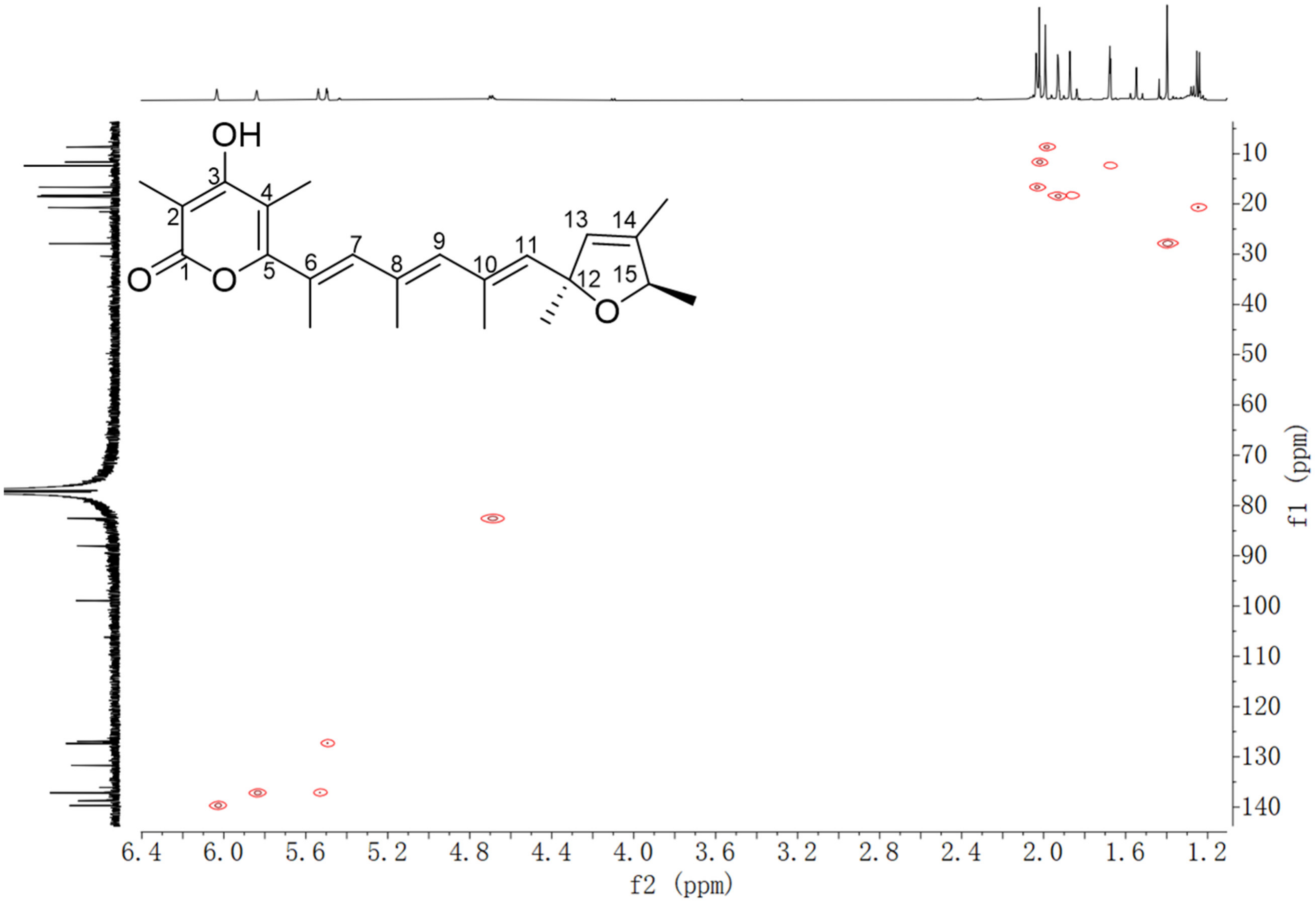

Figure S26. HSQC spectrum of policosidin C (**6**) in $CDCl_3$.

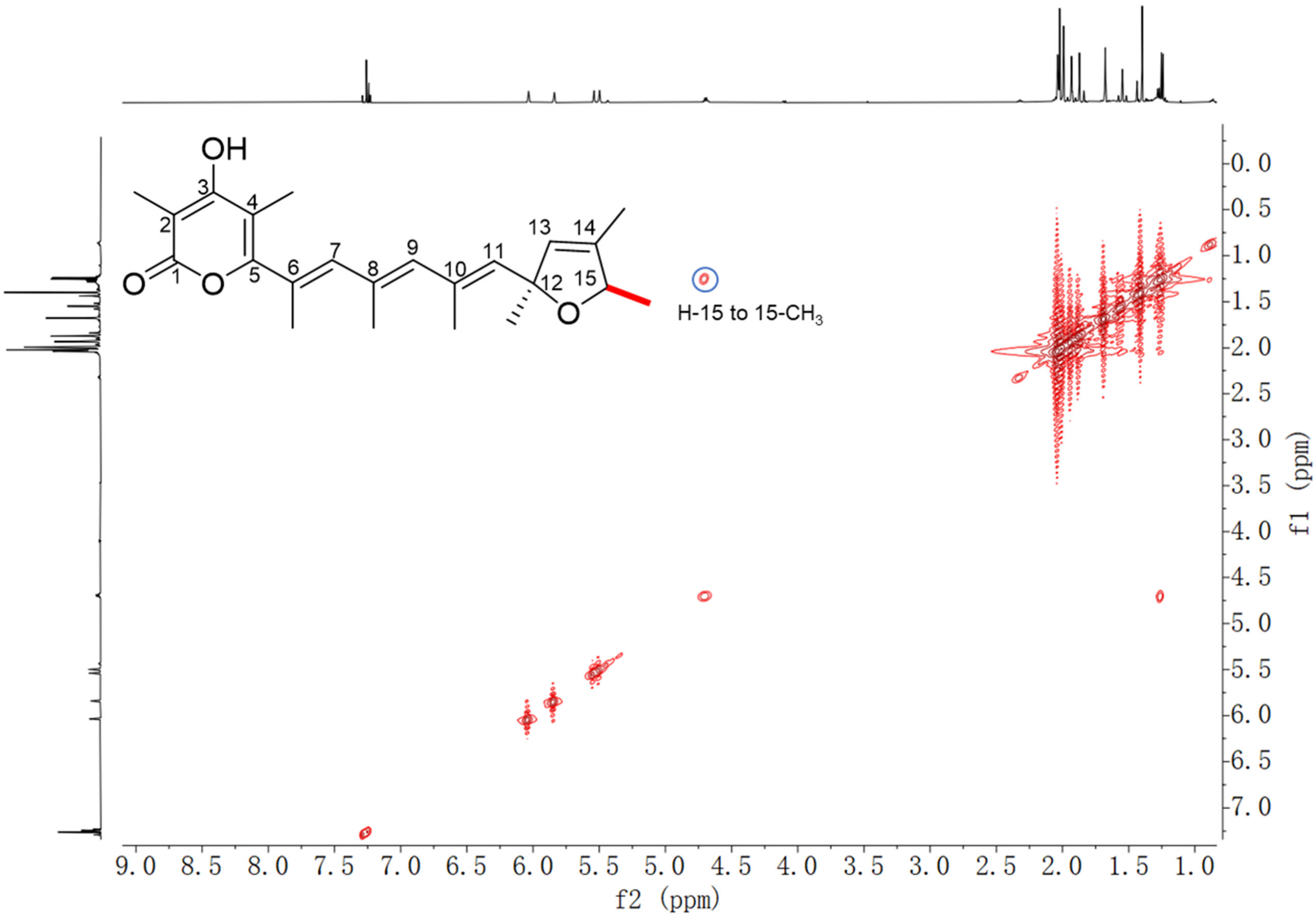

Figure S27. DQF-COSY spectrum of policosidin C (**6**) in $CDCl_3$.

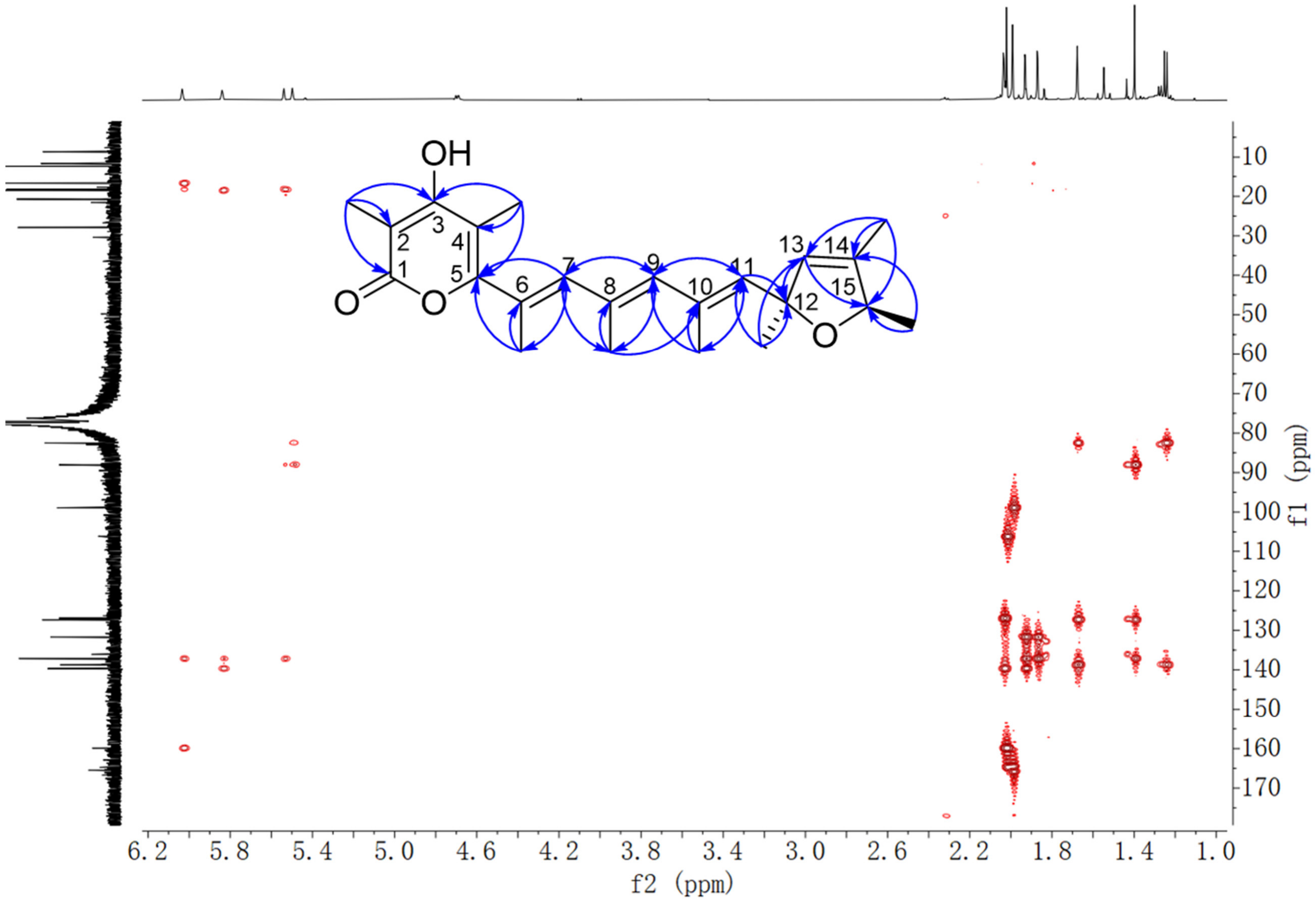

Figure S28. HMBC spectrum of policosidin C (**6**) in $CDCl_3$.

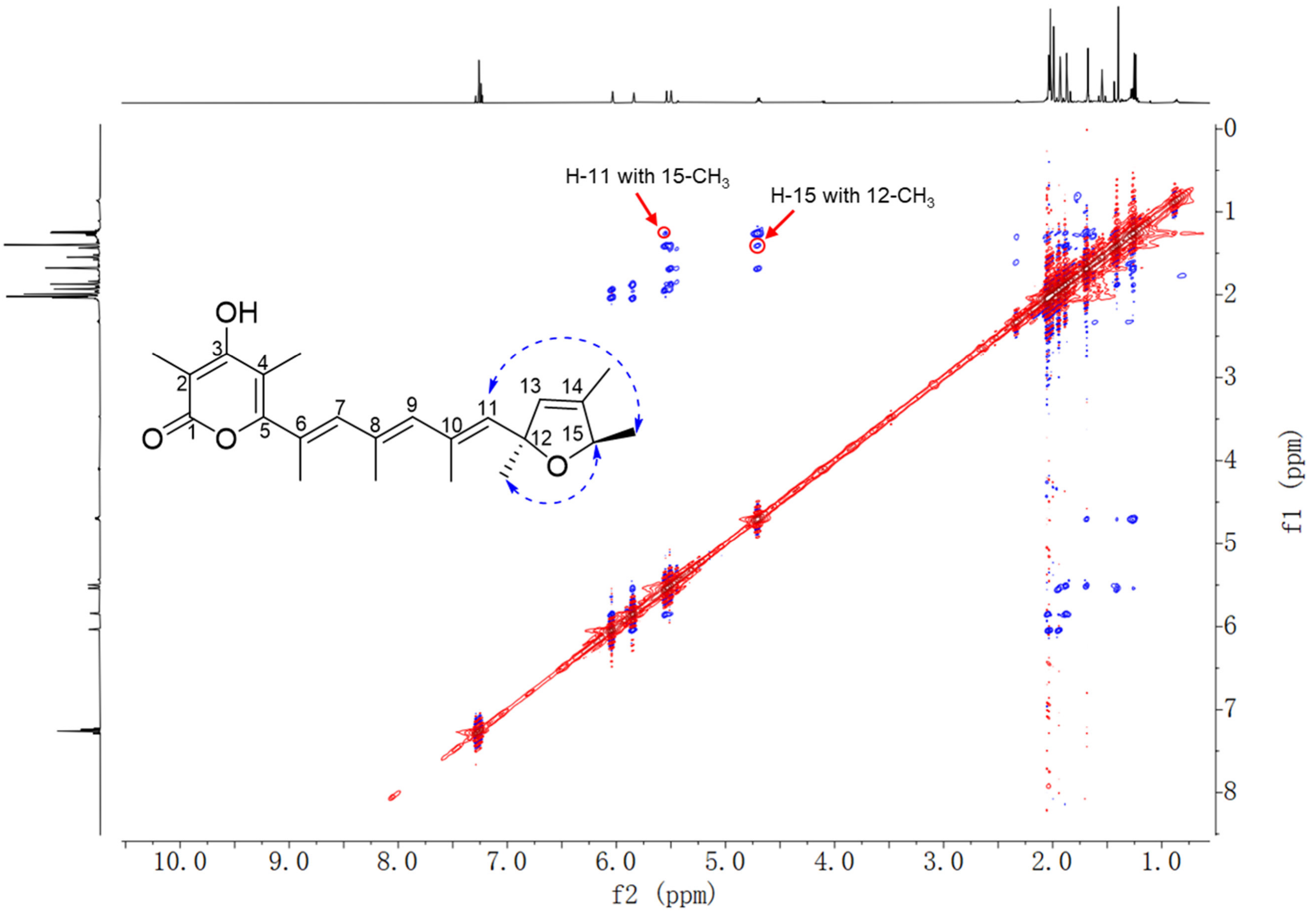

Figure S29. NOESY spectrum of policosidin C (**6**) in $CDCl_3$.

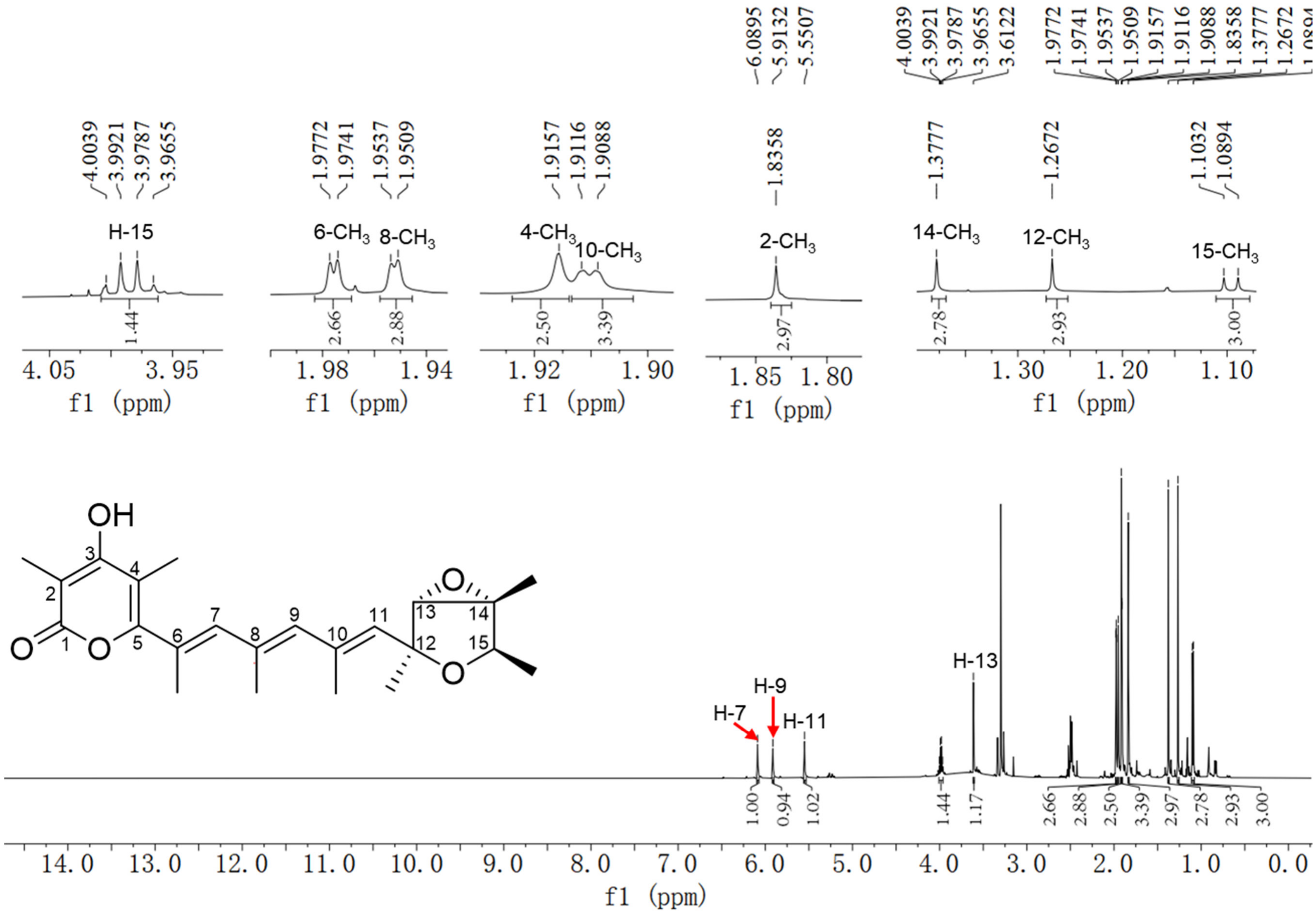

Figure S30. ^{1}H NMR spectrum of policosidin D (**7**) in DMSO-d_6 (500 MHz).

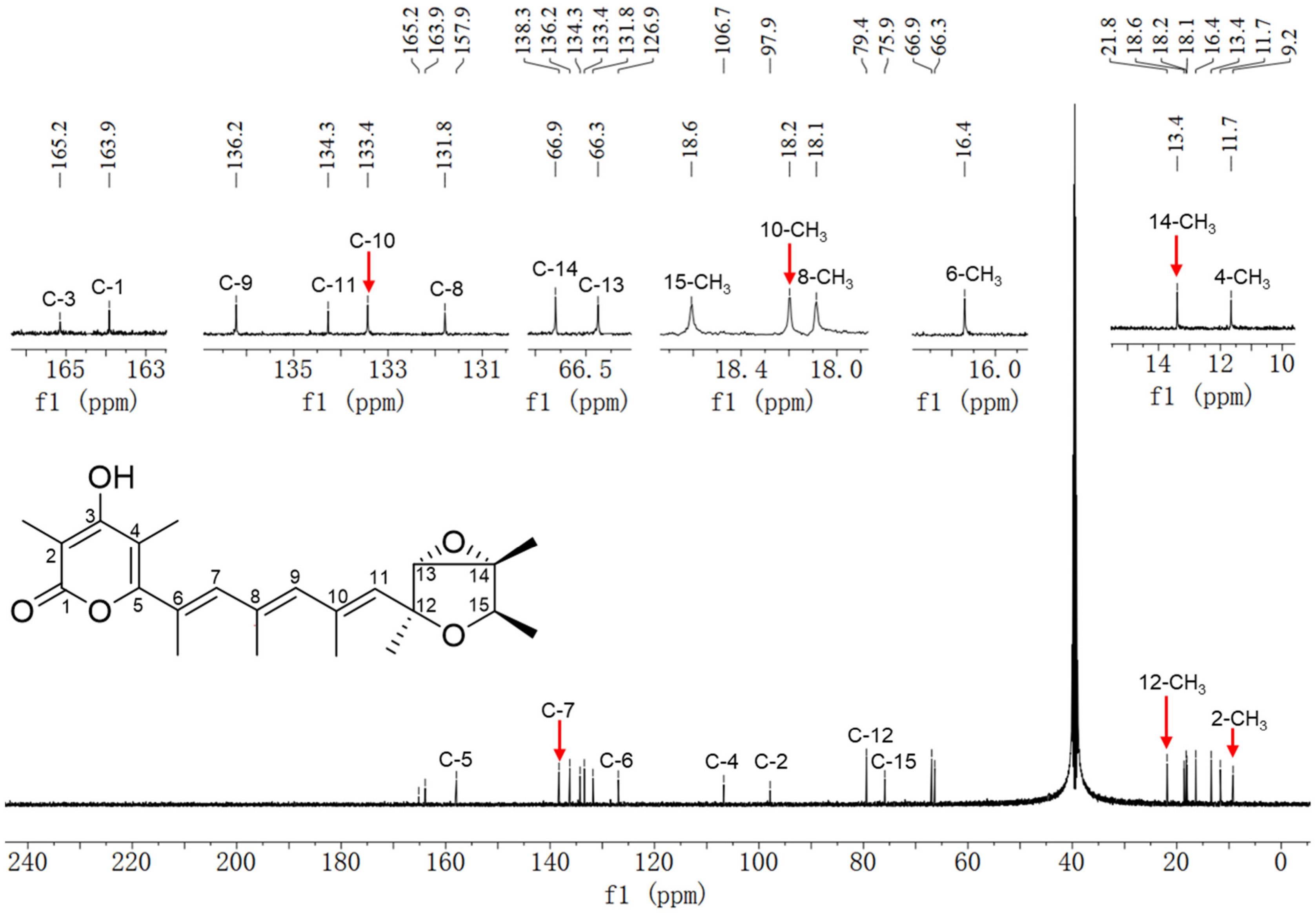

Figure S31. ^{13}C NMR spectrum of policosidin D (**7**) in DMSO-d_6 (125 MHz).

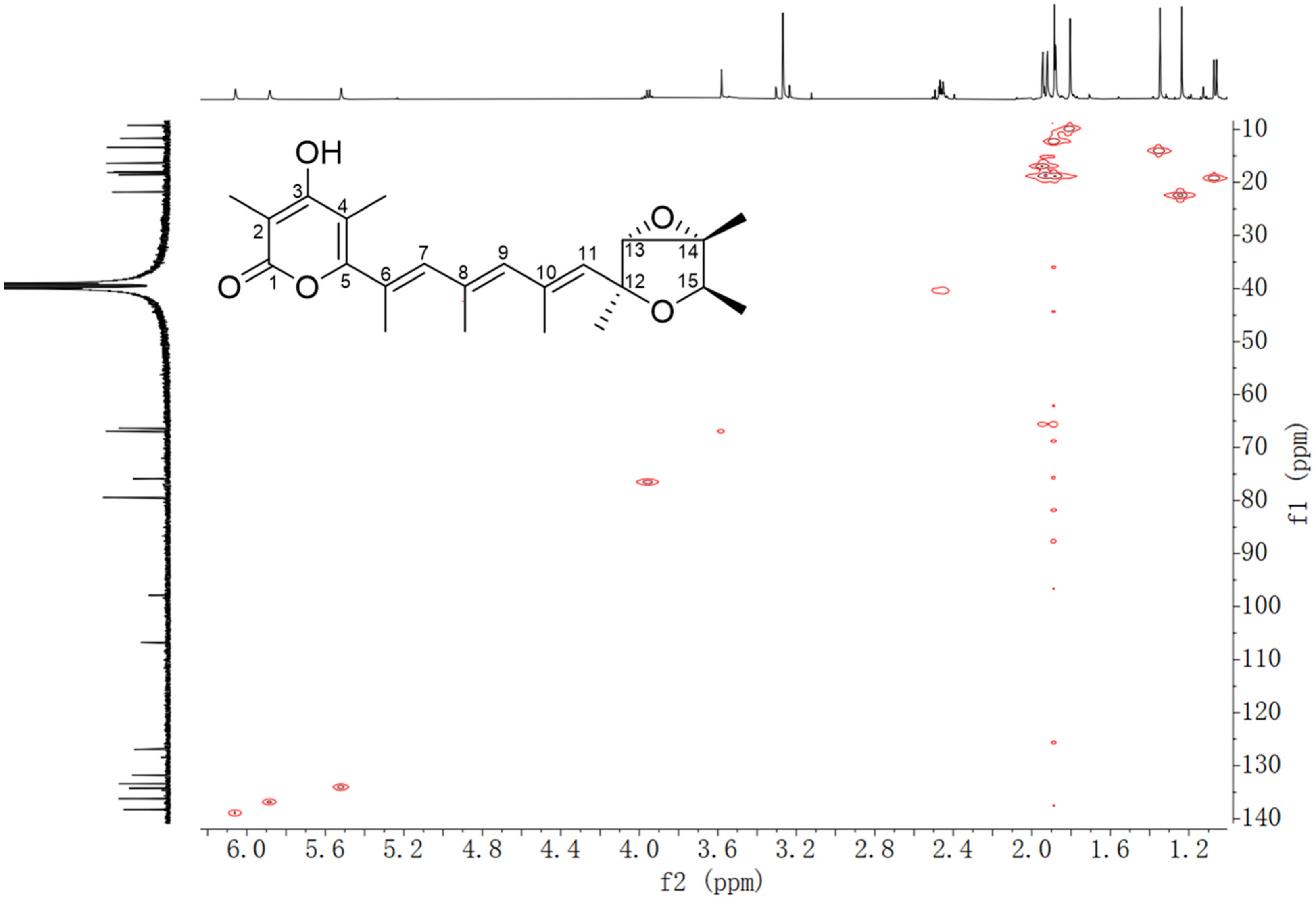

Figure S32. HSQC spectrum of policosidin D (**7**) in DMSO-*d*$_6$.

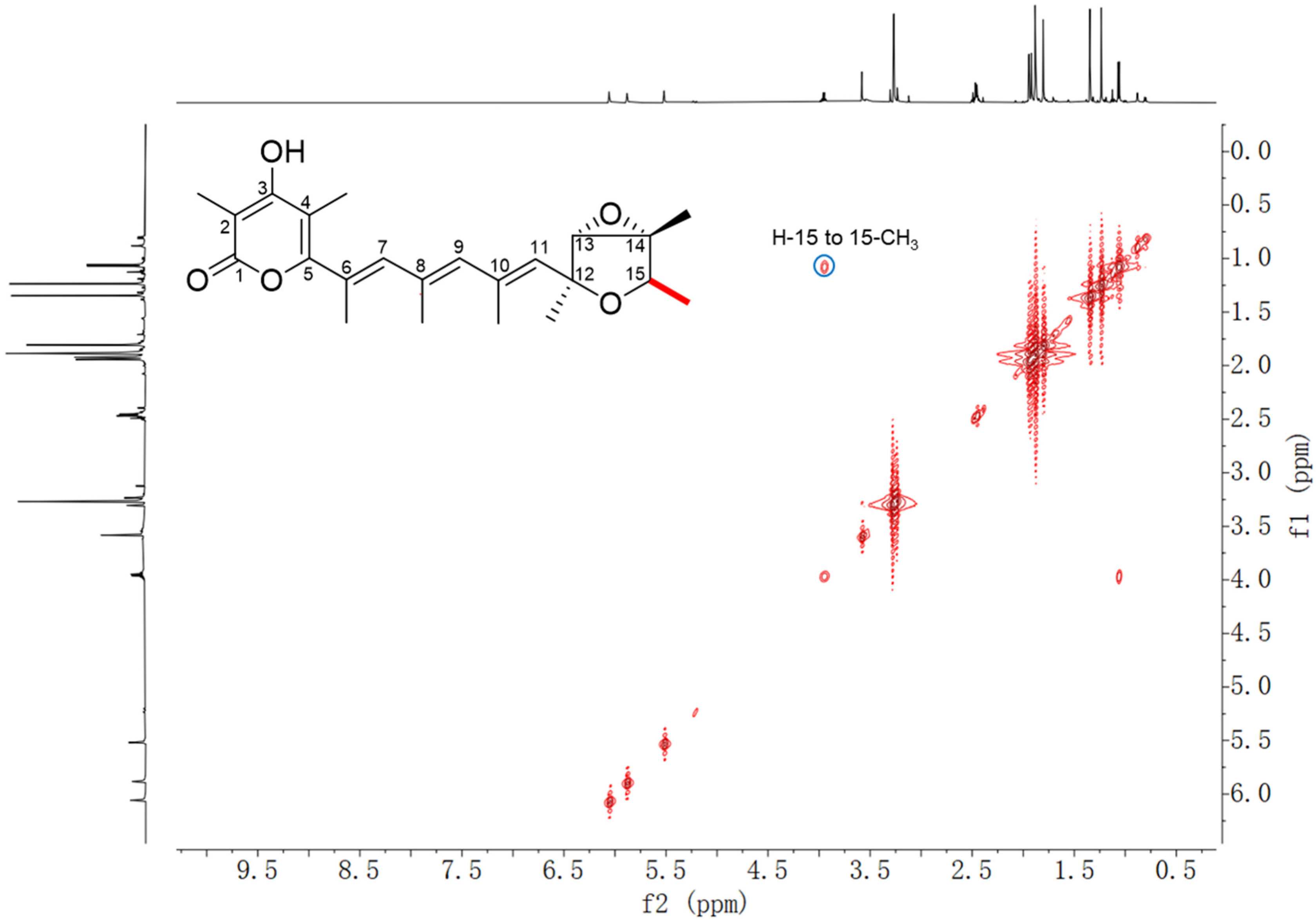

Figure S33. DQF-COSY spectrum of policosidin D (**7**) in DMSO-d_6.

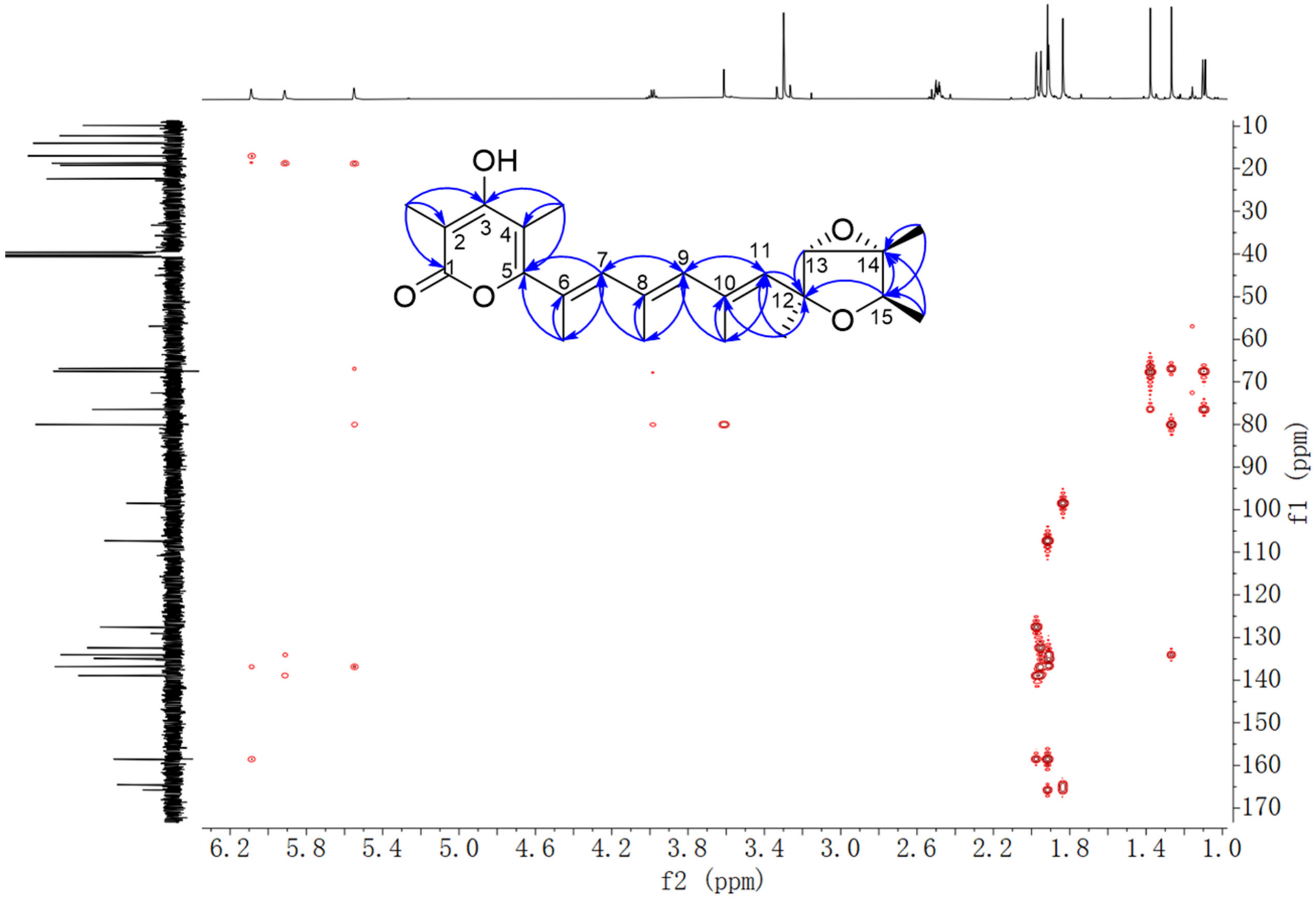

Figure S34. HMBC spectrum of policosidin D (**7**) in DMSO-d_6.

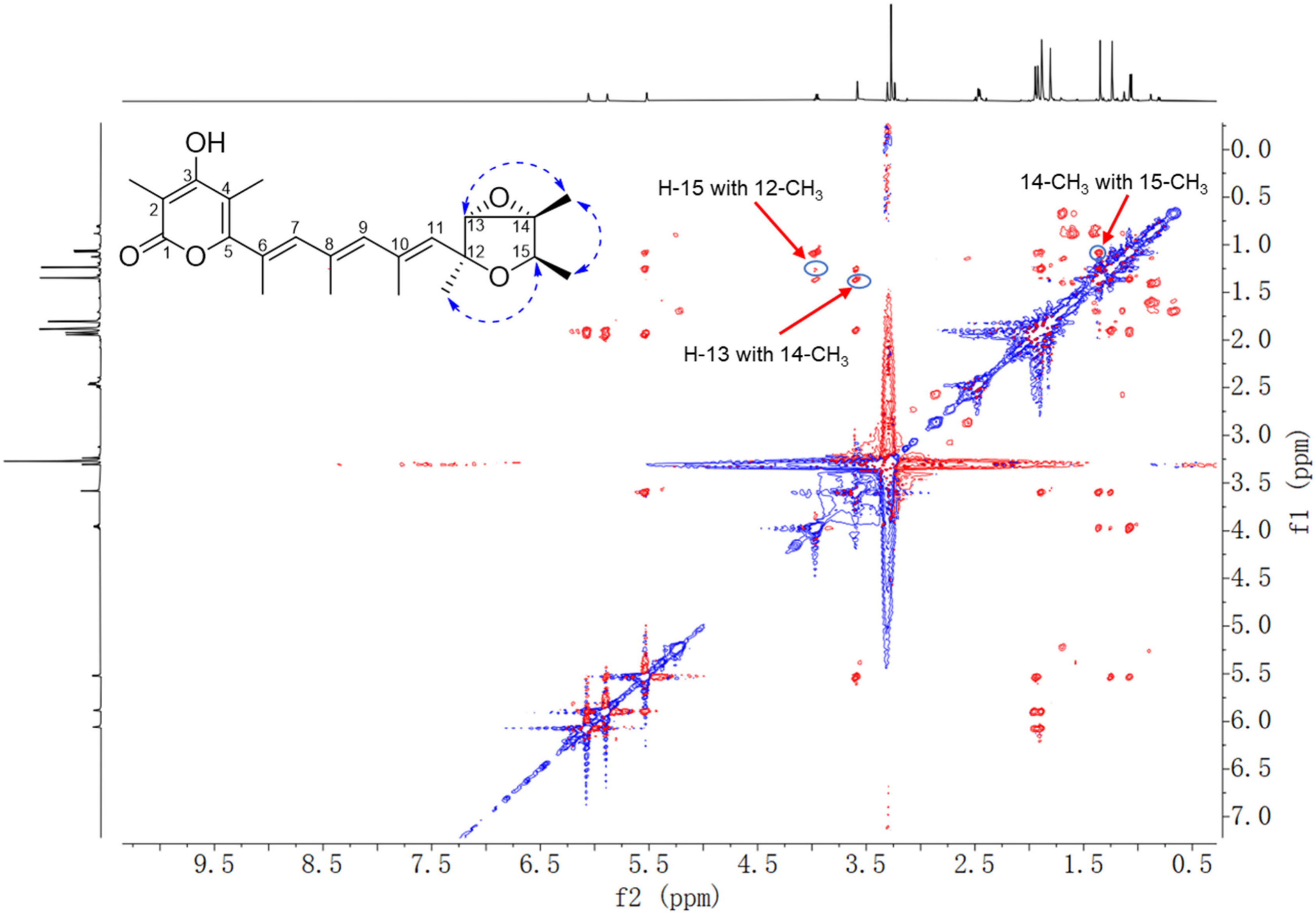

Figure S35. NOESY spectrum of policosidin D (**7**) in DMSO-d_6.

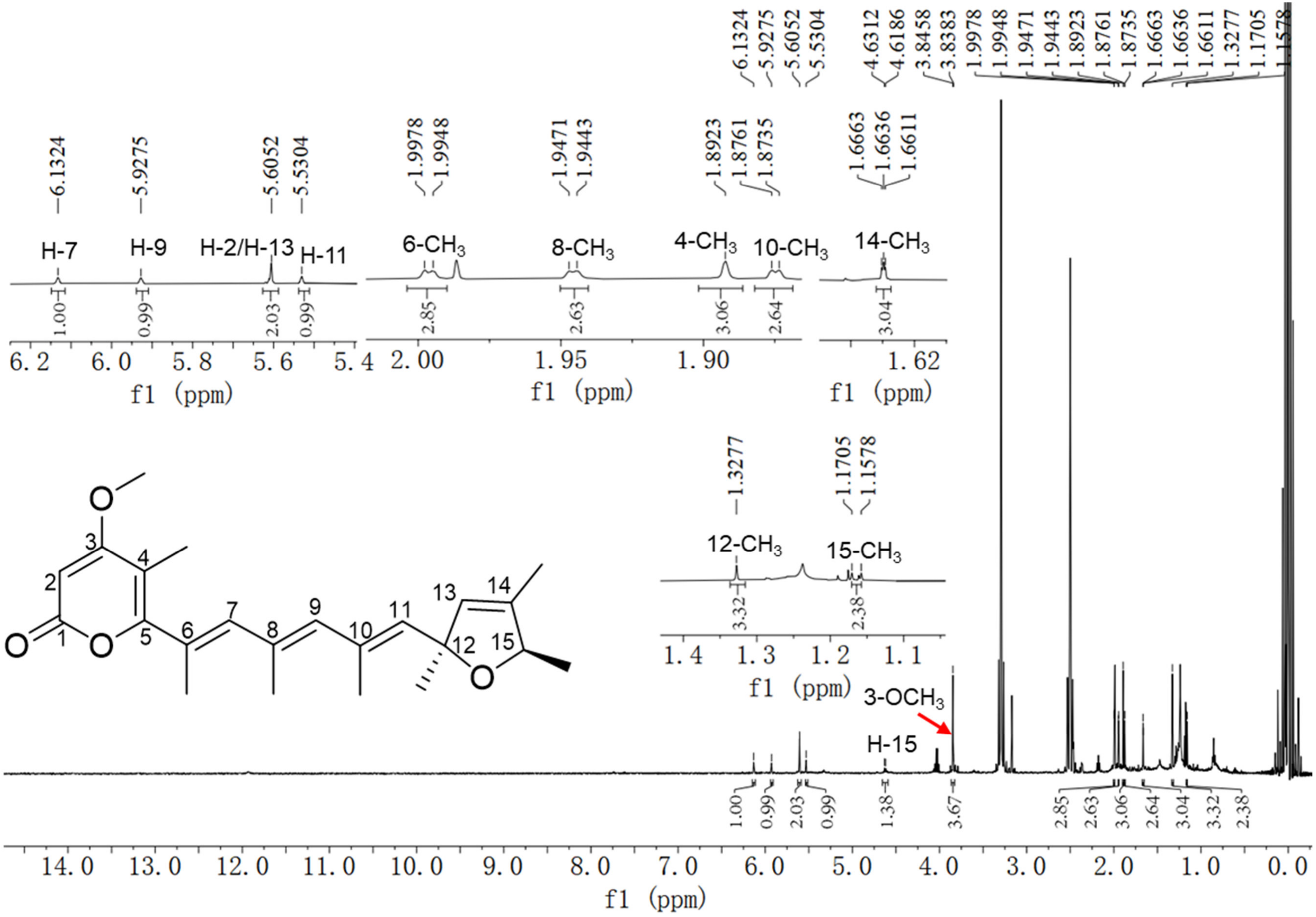

Figure S36. ^{1}H NMR spectrum of poloncosidin B (**8**) in DMSO-*d*$_6$ (500 MHz).

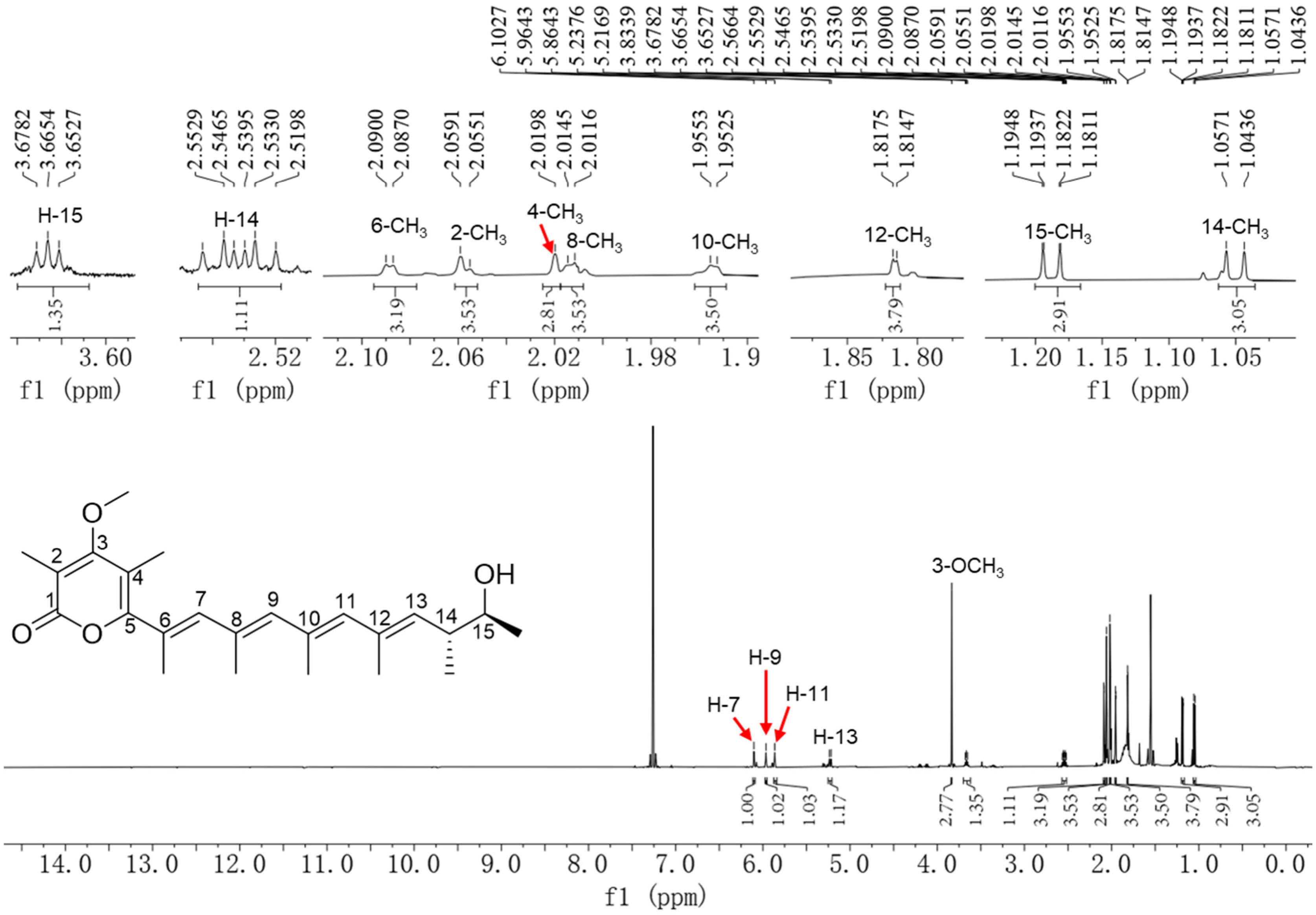

Figure S37. ^{1}H NMR spectrum of policosidin E (**9**) in $CDCl_3$ (500 MHz).

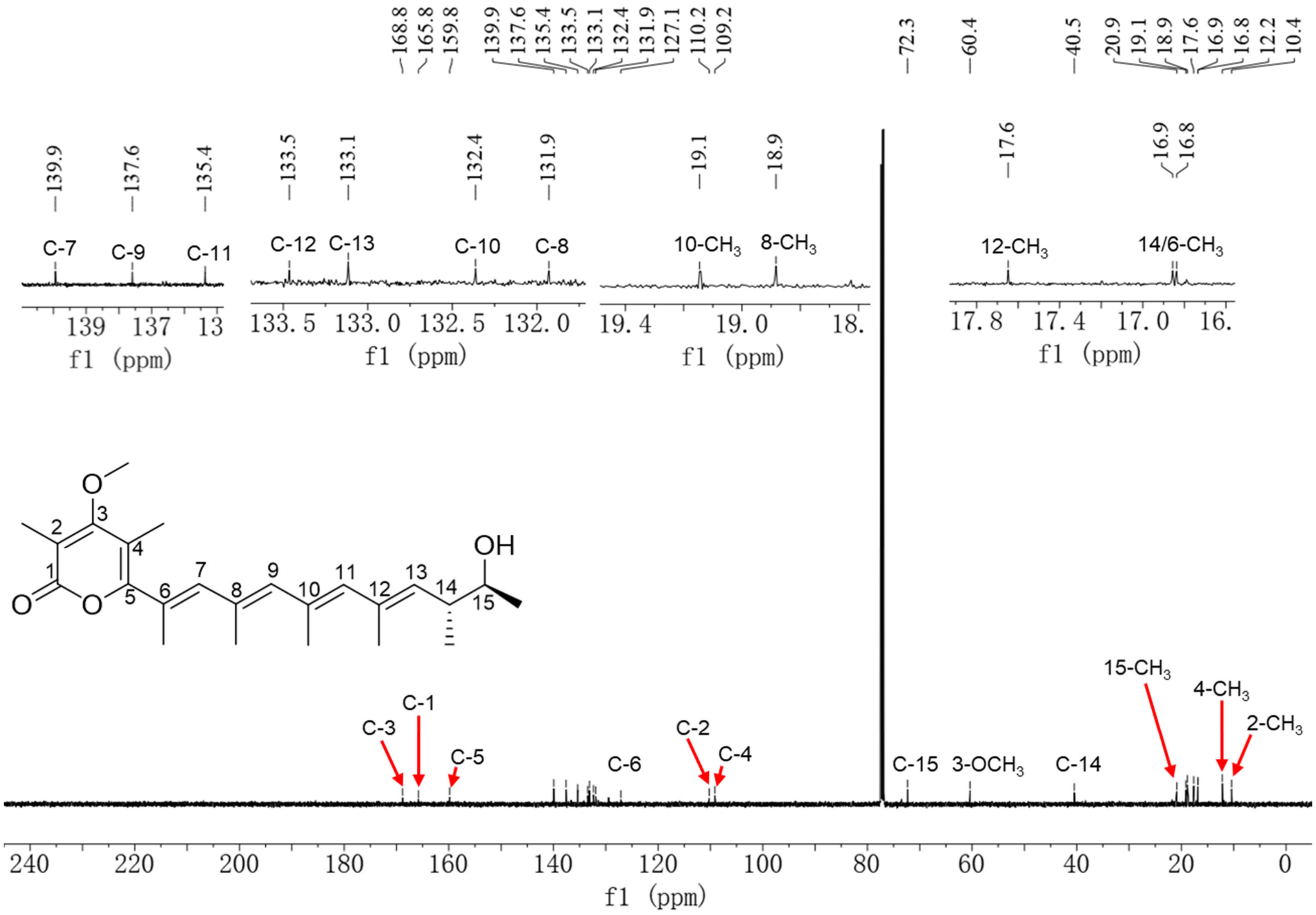

Figure S38. ^{13}C NMR spectrum of policosidin E (**9**) in $CDCl_3$ (125 MHz).

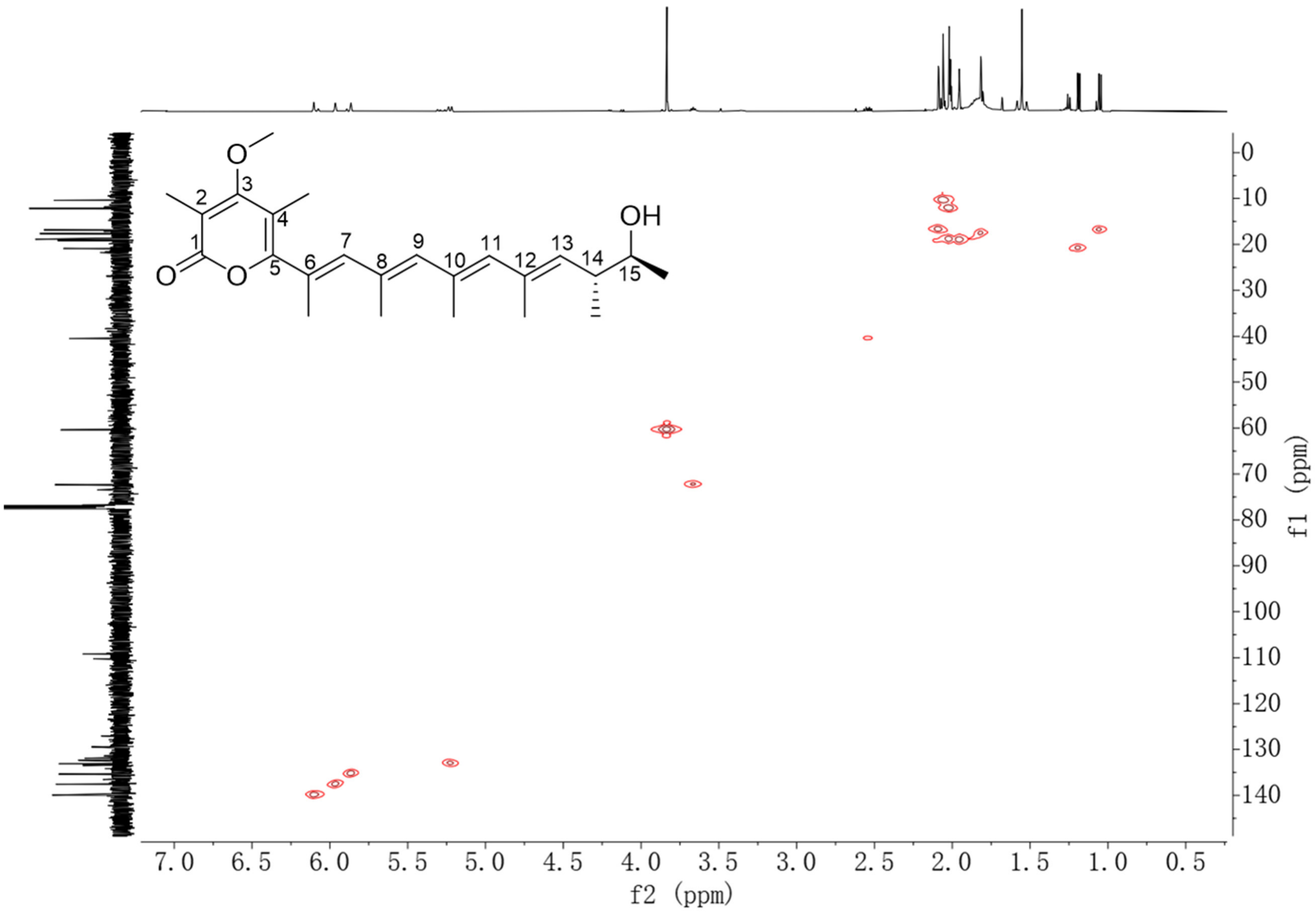

Figure S39. HSQC spectrum of policosidin E (**9**) in $CDCl_3$.

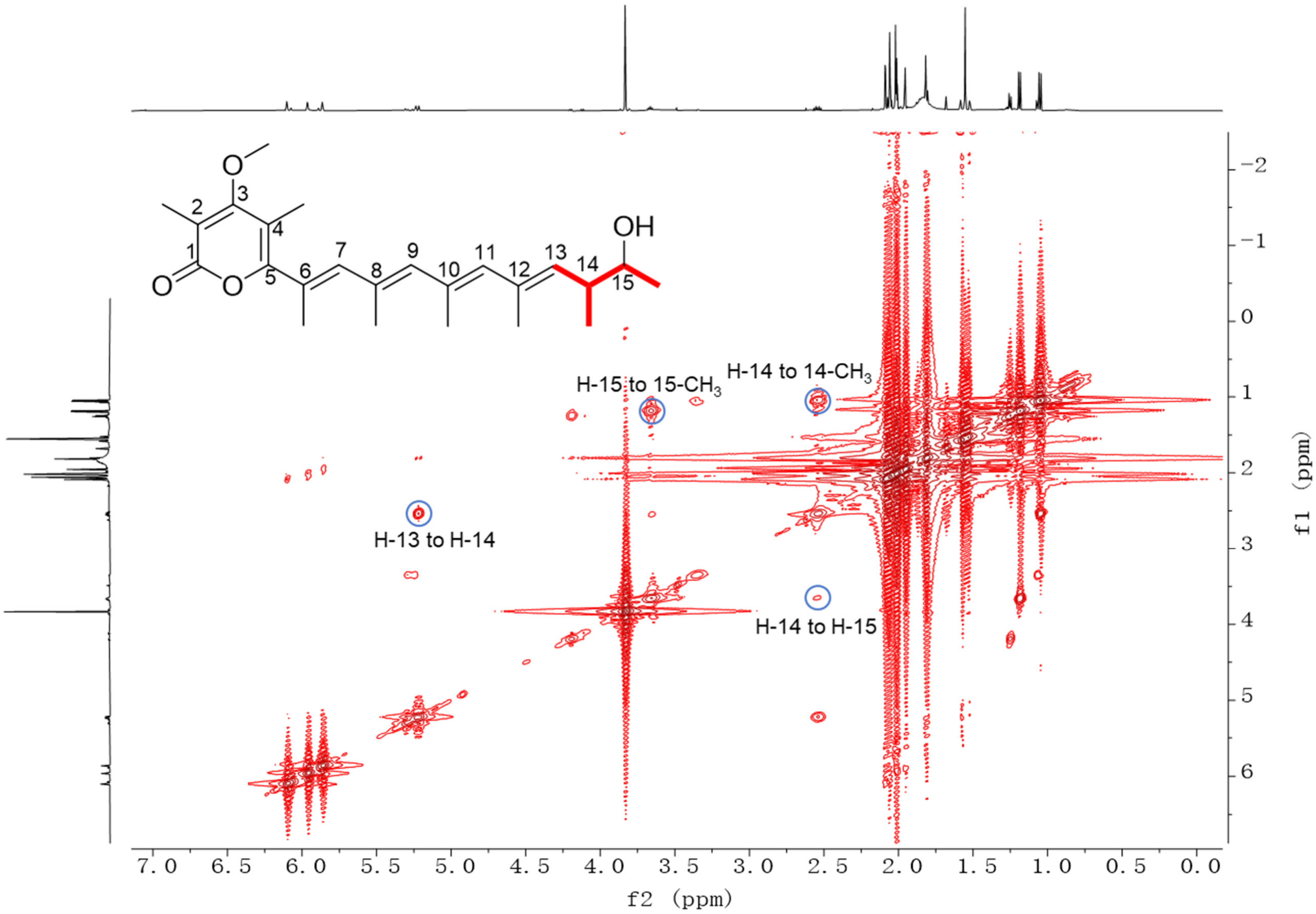

Figure S40. DQF-COSY spectrum of policosidin E (**9**) in $CDCl_3$.

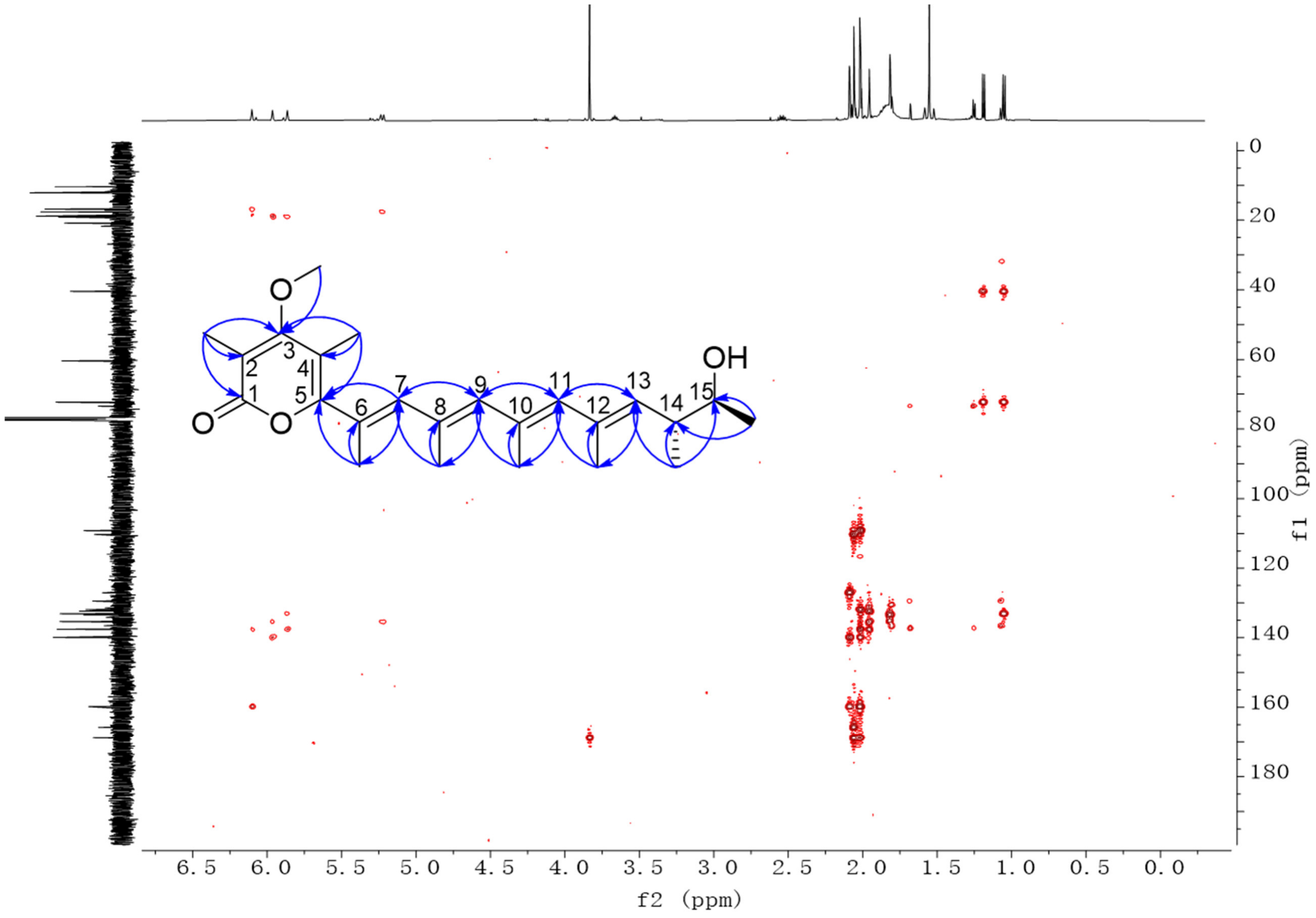

Figure S41. HMBC spectrum of policosidin E (**9**) in $CDCl_3$.

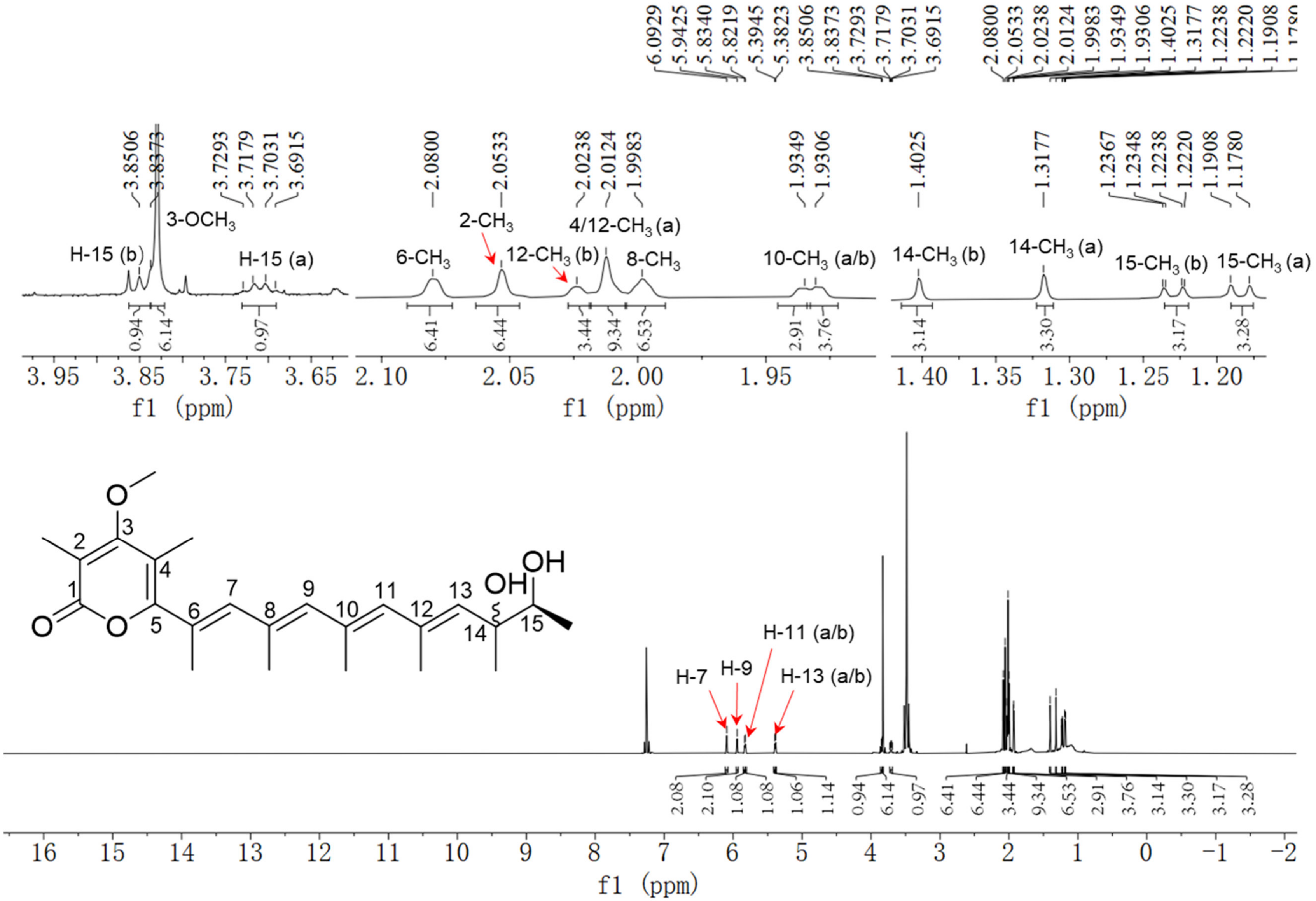

Figure S42. ^{1}H NMR spectrum of policosidin F (**10**) as a diastereomeric pair in $CDCl_3$ (500 MHz).

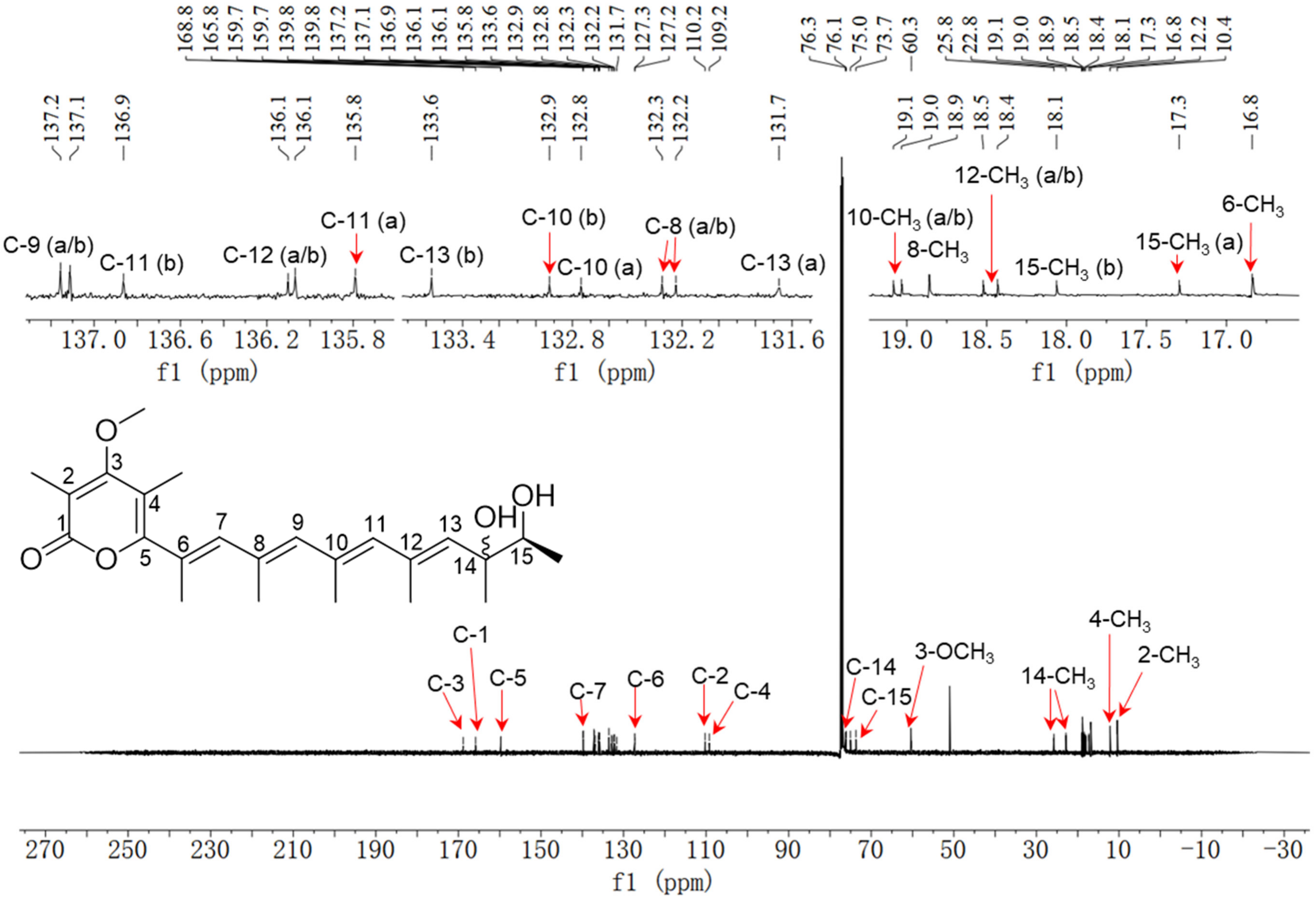

Figure S43. ^{13}C NMR spectrum of policosidin F (**10**) as a diastereomeric pair in $CDCl_3$ (125 MHz).

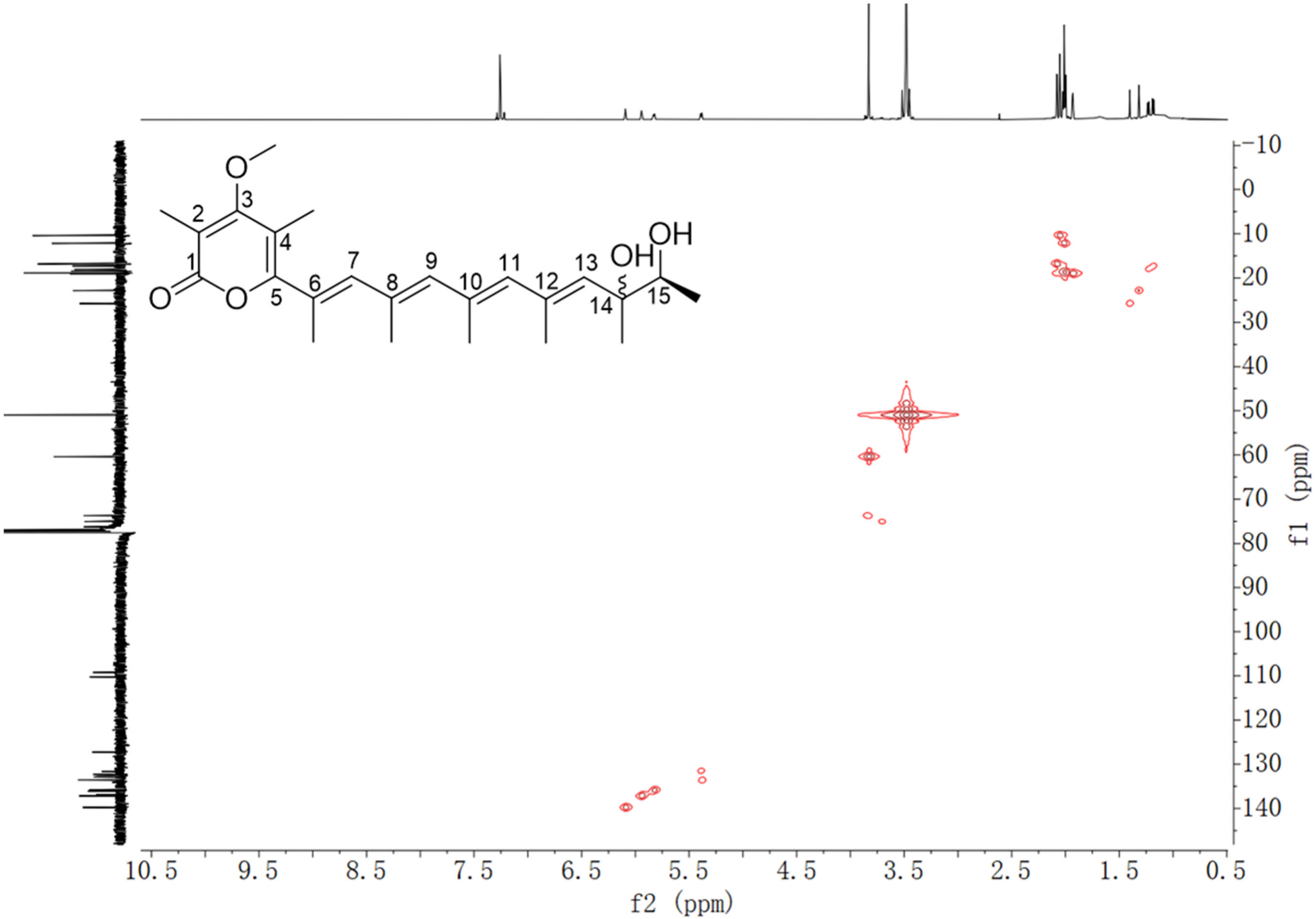

Figure S44. HSQC spectrum of policosidin F (**10**) as a diastereomeric pair in $CDCl_3$.

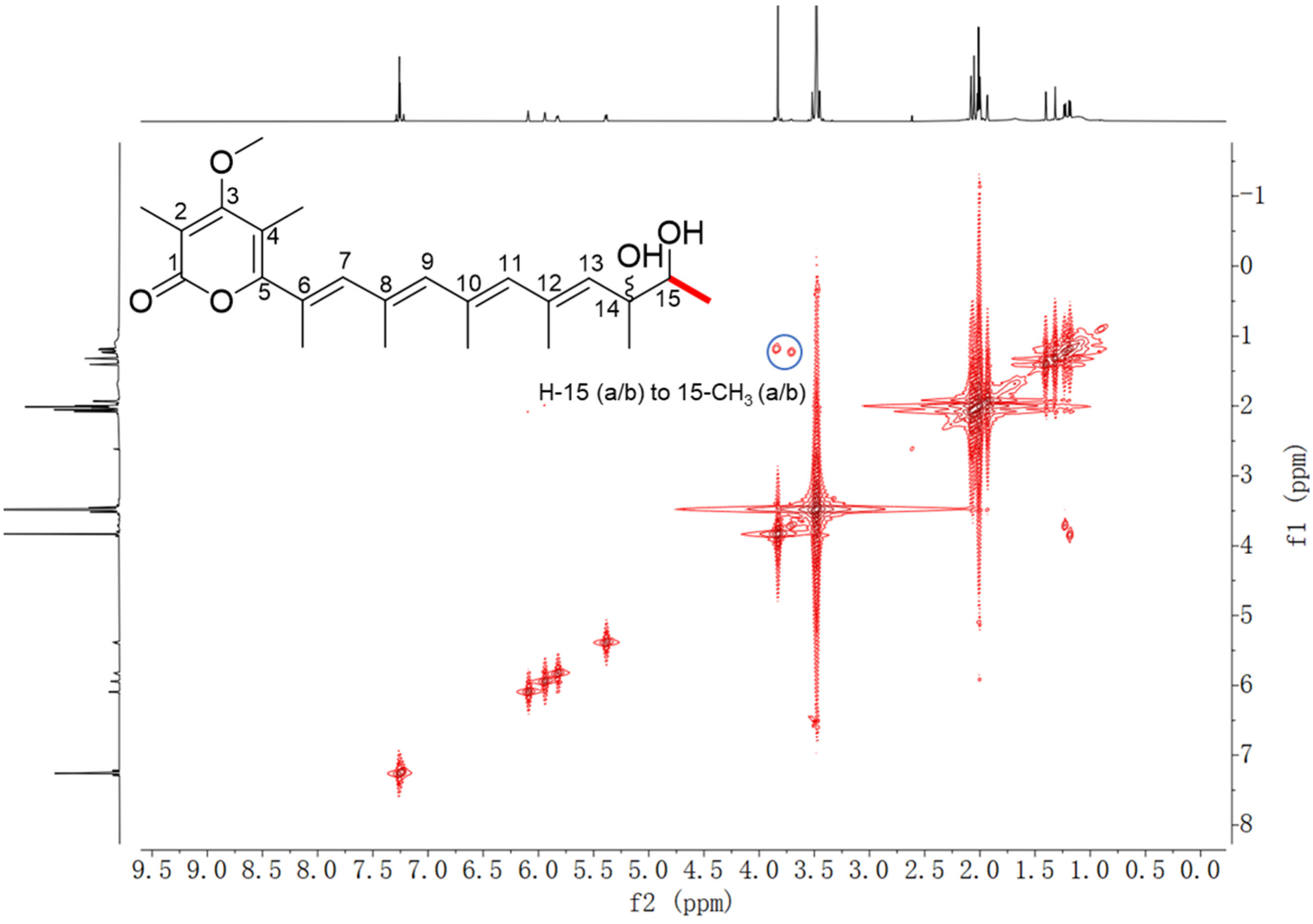

Figure S45. DQF-COSY spectrum of policosidin F (**10**) as a diastereomeric pair in $CDCl_3$.

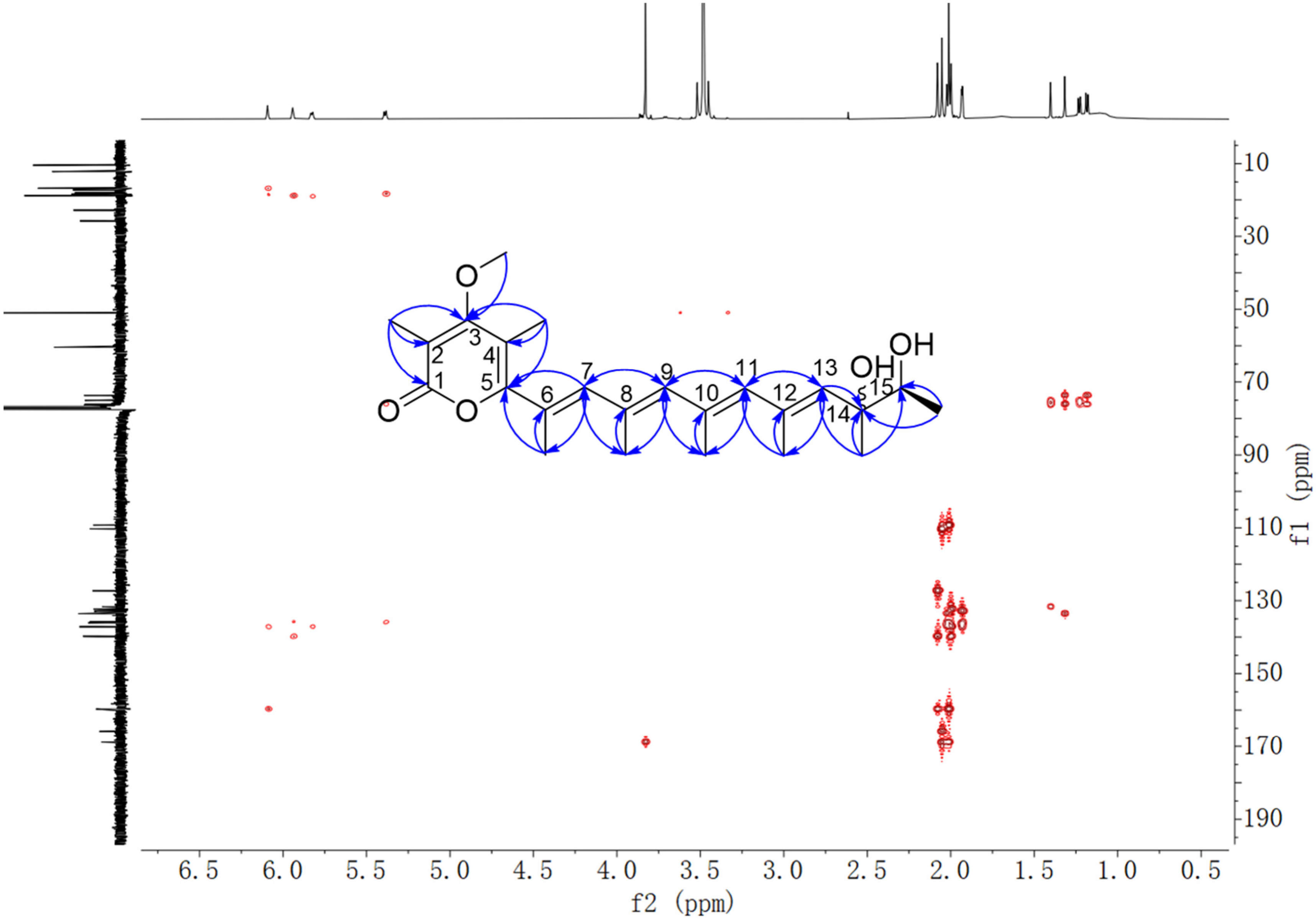

Figure S46. HMBC spectrum of policosidin F (**10**) as a diastereomeric pair in $CDCl_3$.

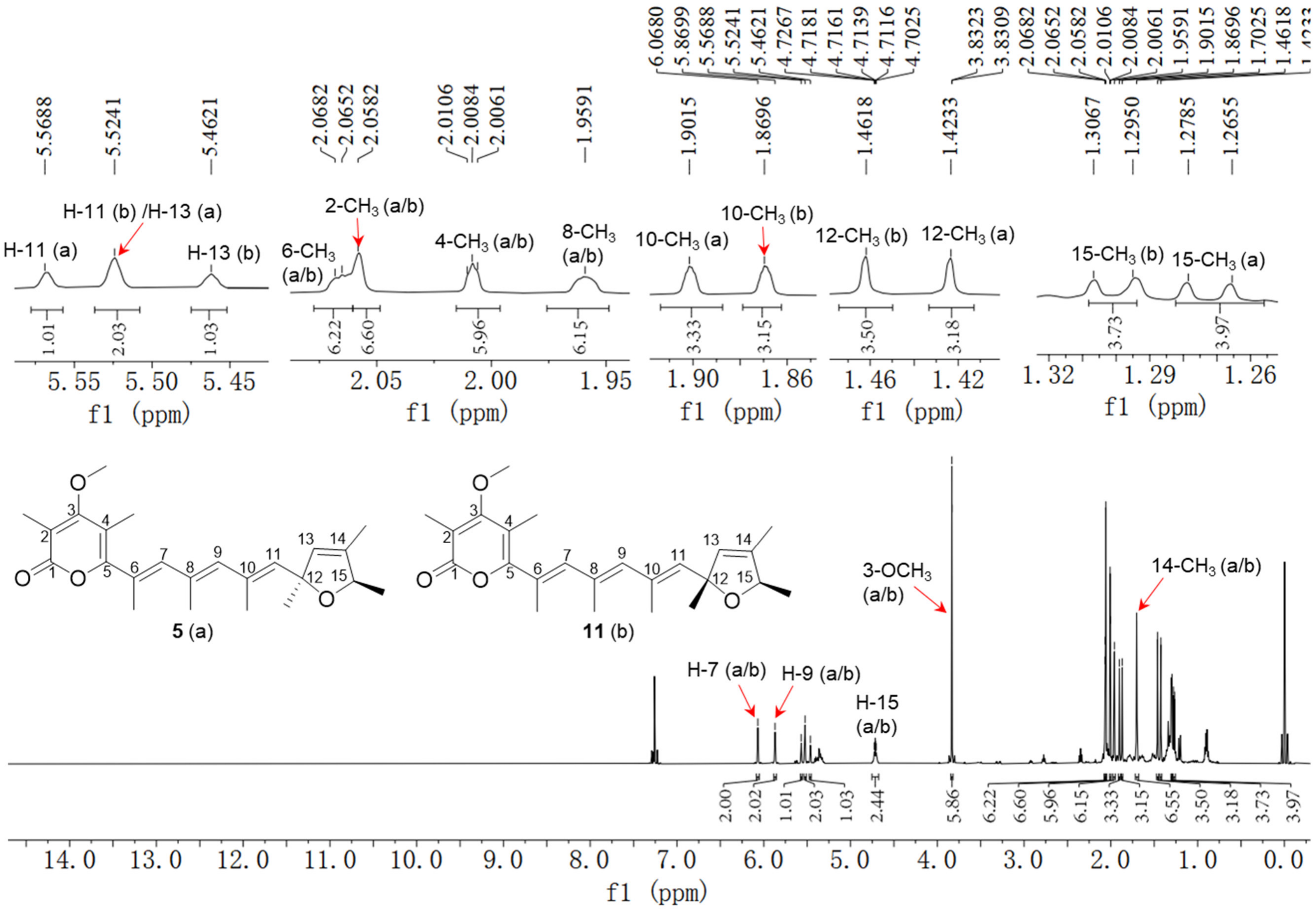

Figure S47. ^{1}H NMR spectrum of a diastereomeric pair containing poloncosidin A (**5**) and policosidin G (**11**) in $CDCl_3$ (500 MHz).

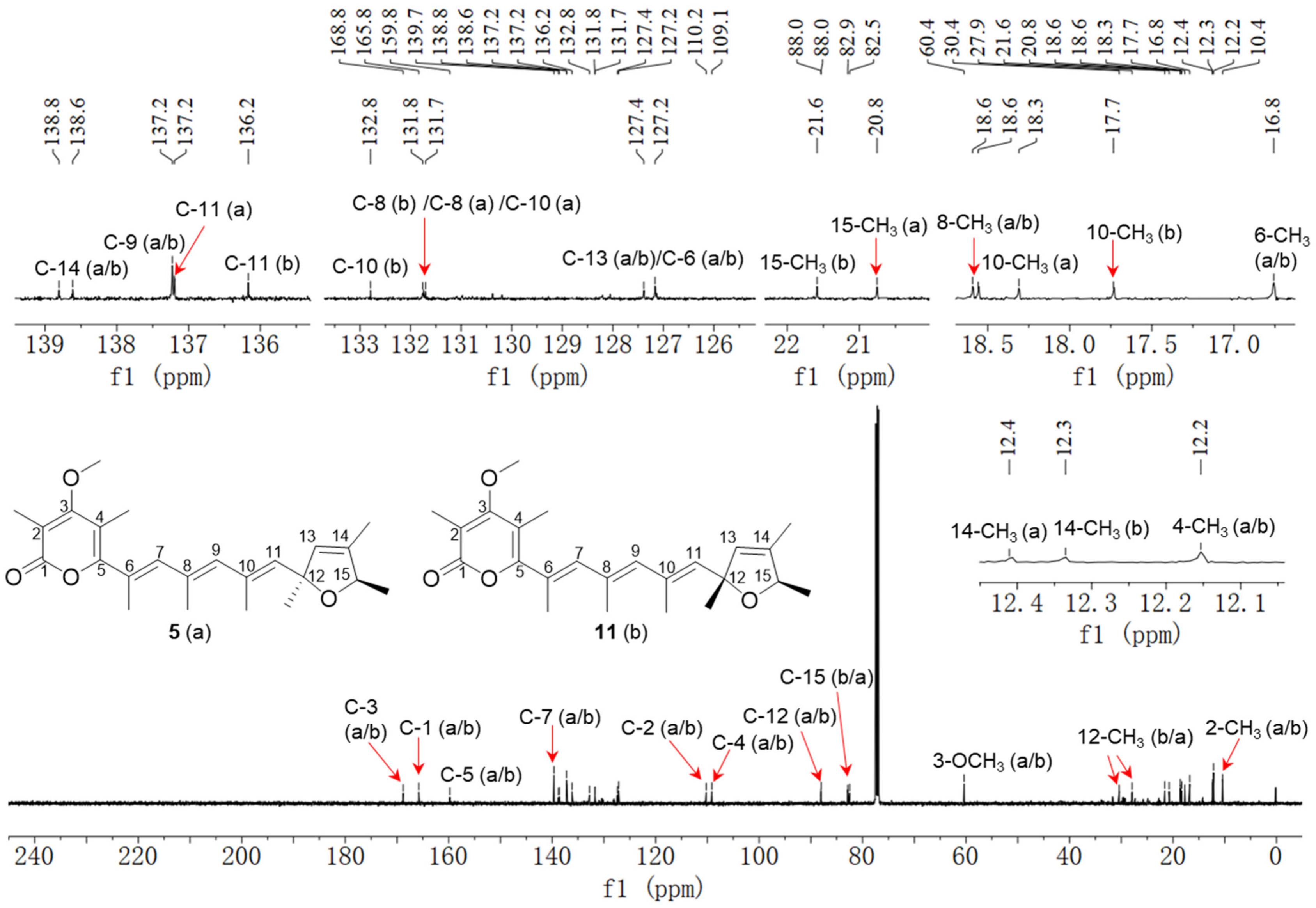

Figure S48. ^{13}C NMR spectrum of a diastereomeric pair containing poloncosidin A (**5**) and policosidin G (**11**) in $CDCl_3$ (125 MHz).

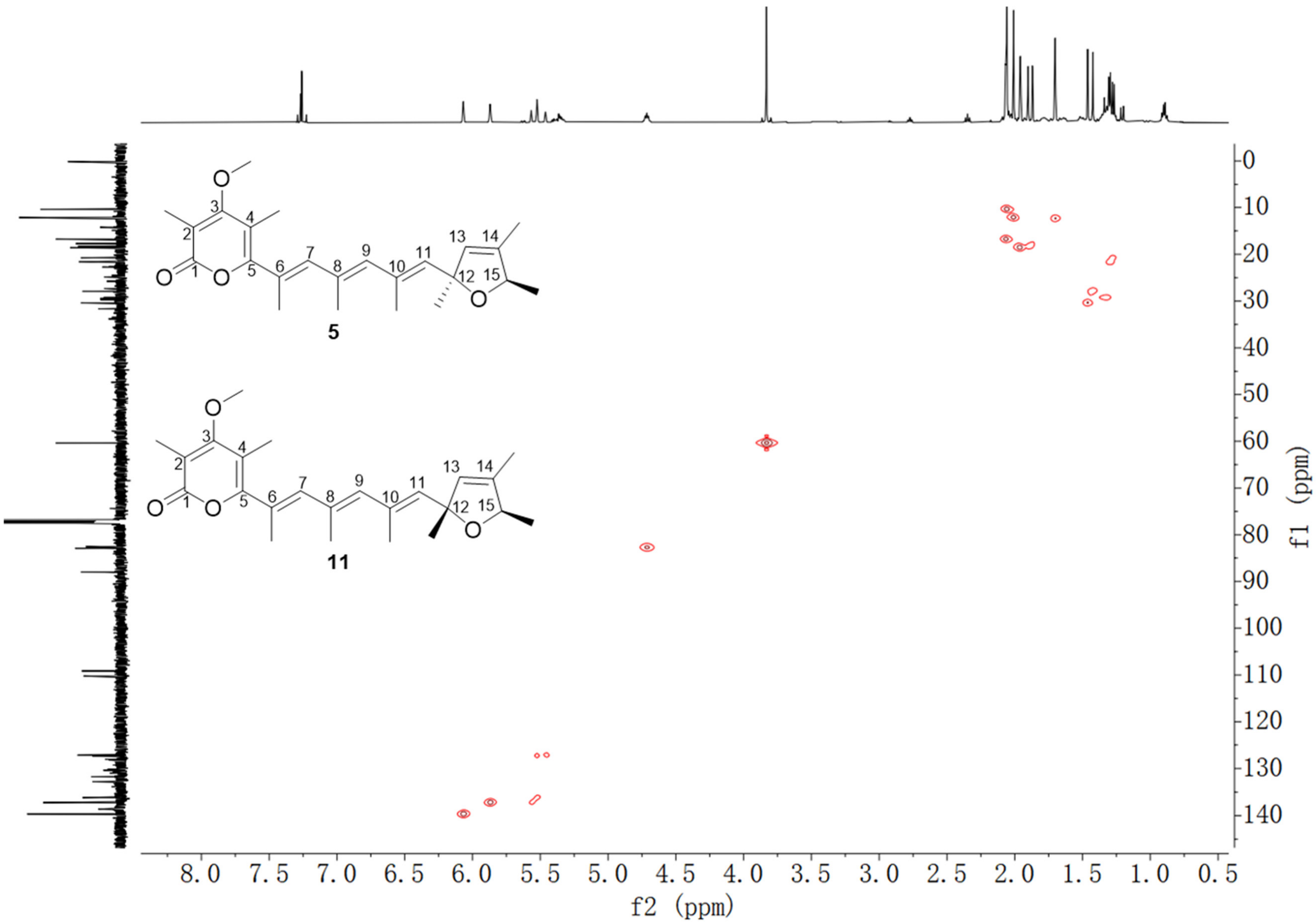

Figure S49. HSQC spectrum of a diastereomeric pair containing poloncosidin A (**5**) and policosidin G (**11**) in $CDCl_3$.

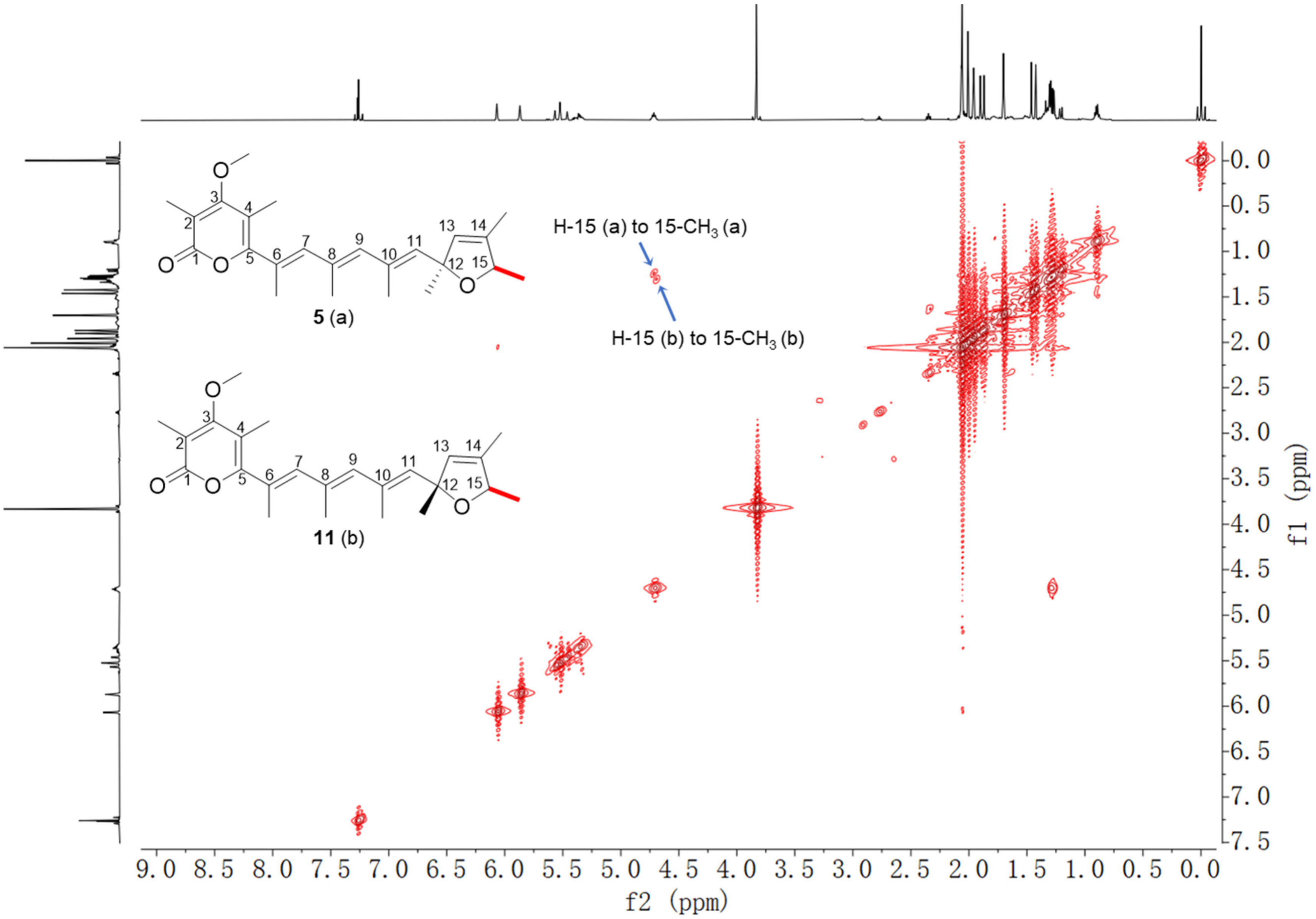

Figure S50. DQF-COSY spectrum of a diastereomeric pair containing poloncosidin A (**5**) and policosidin G (**11**) in $CDCl_3$.

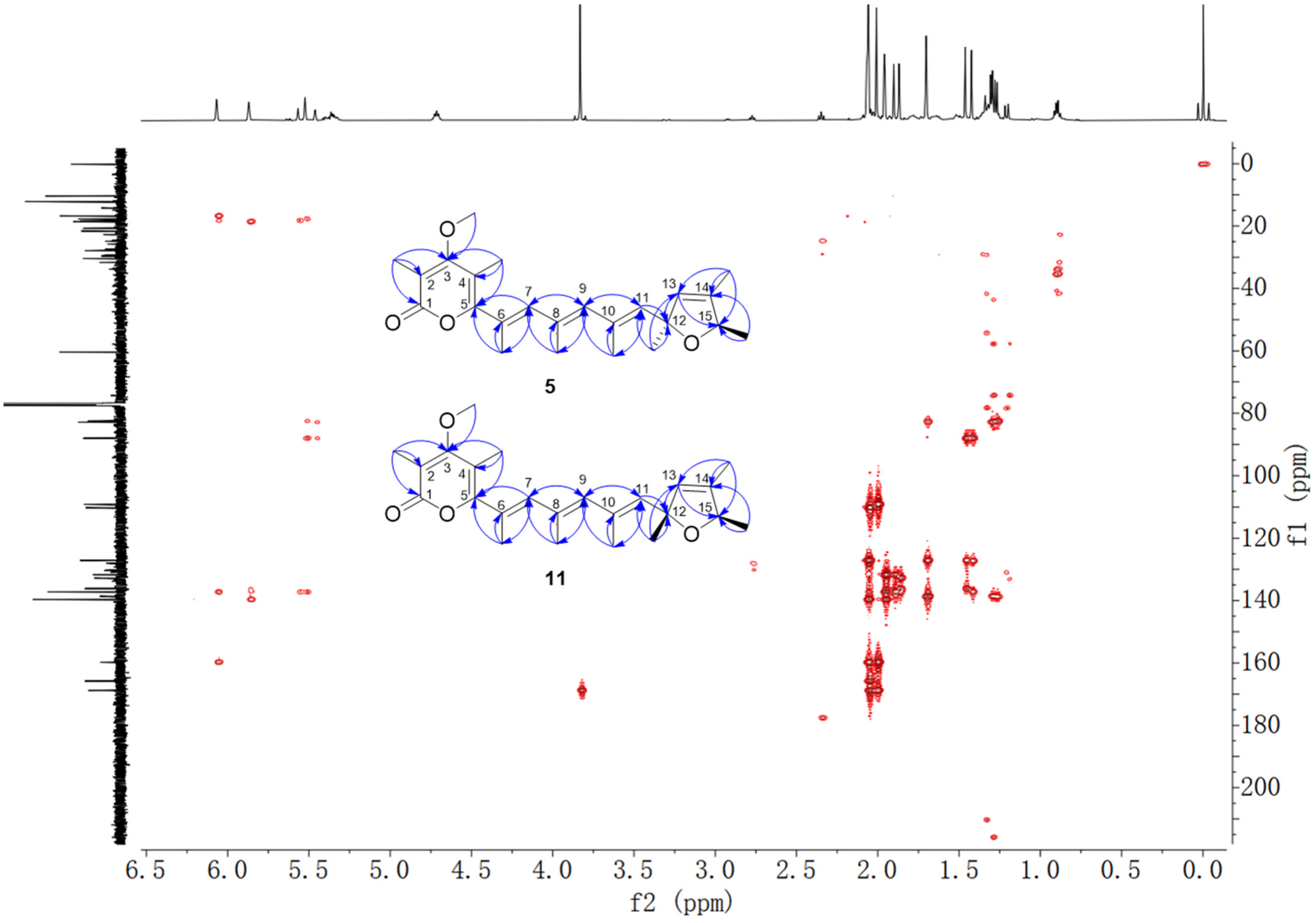

Figure S51. HMBC spectrum of a diastereomeric pair containing poloncosidin A (**5**) and policosidin G (**11**) in $CDCl_3$.

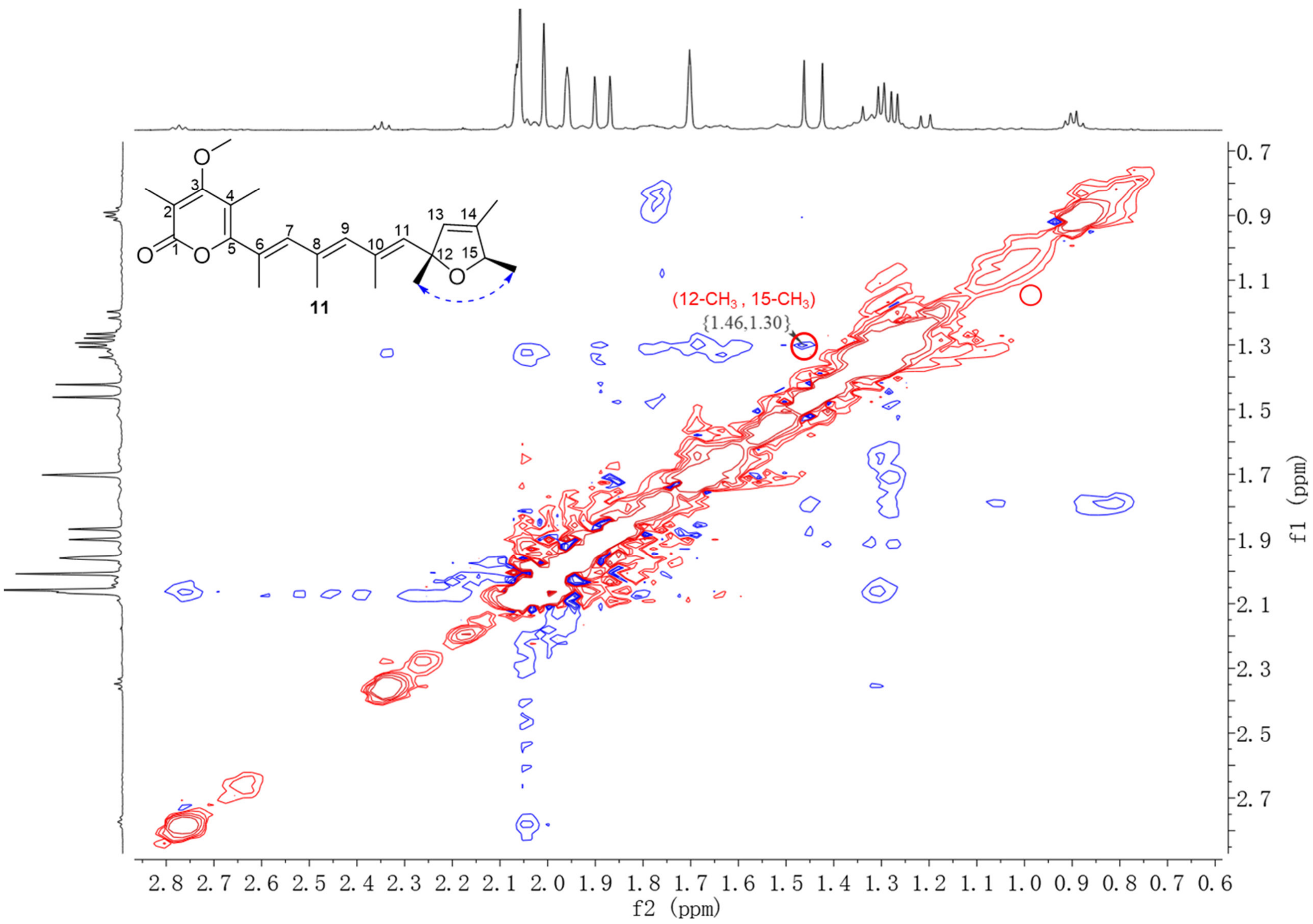

Figure S52. NOESY spectrum of a diastereomeric pair containing poloncosidin A (**5**) and policosidin G (**11**) in $CDCl_3$.

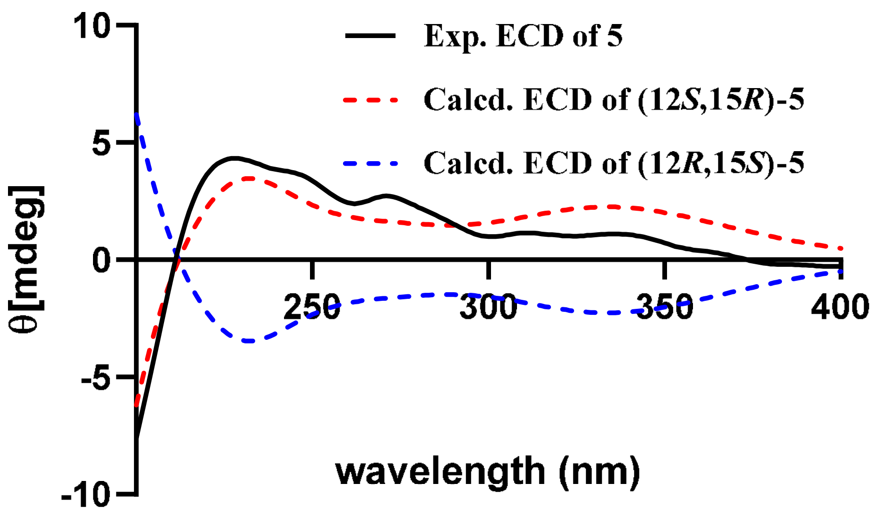

Figure S53. The experimental and calculated ECD spectra of **5** in MeOH.

The experimental ECD spectrum of **5** corresponds well to the calculated one of (12*S*, 15*R*).

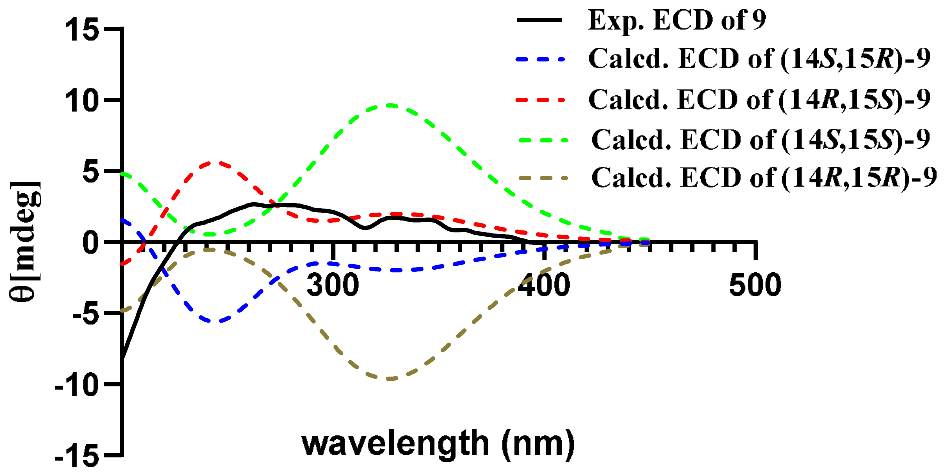

Figure S54. The experimental and calculated ECD spectra of **9** in MeOH.

The experimental ECD spectrum of **9** corresponds well to the calculated one of (14*R*, 15*S*).

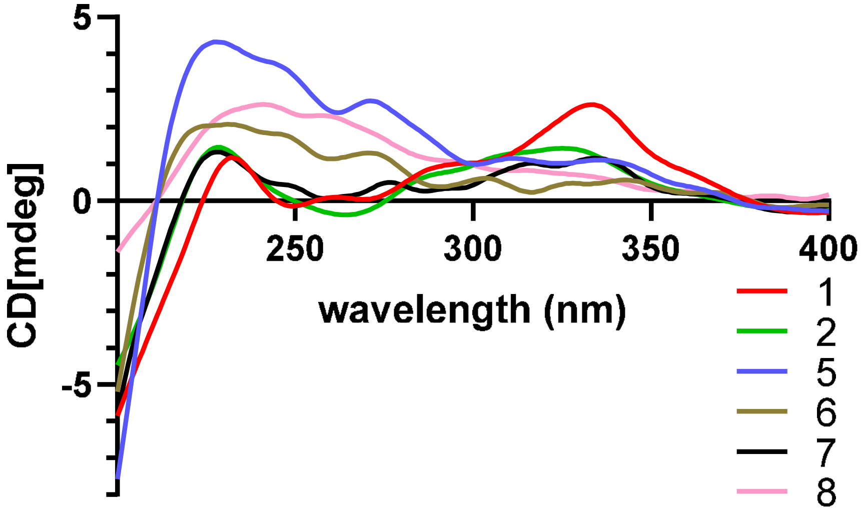

Figure S55. The experimental ECD spectra of compounds **1**, **2**, **5**–**8** in MeOH.

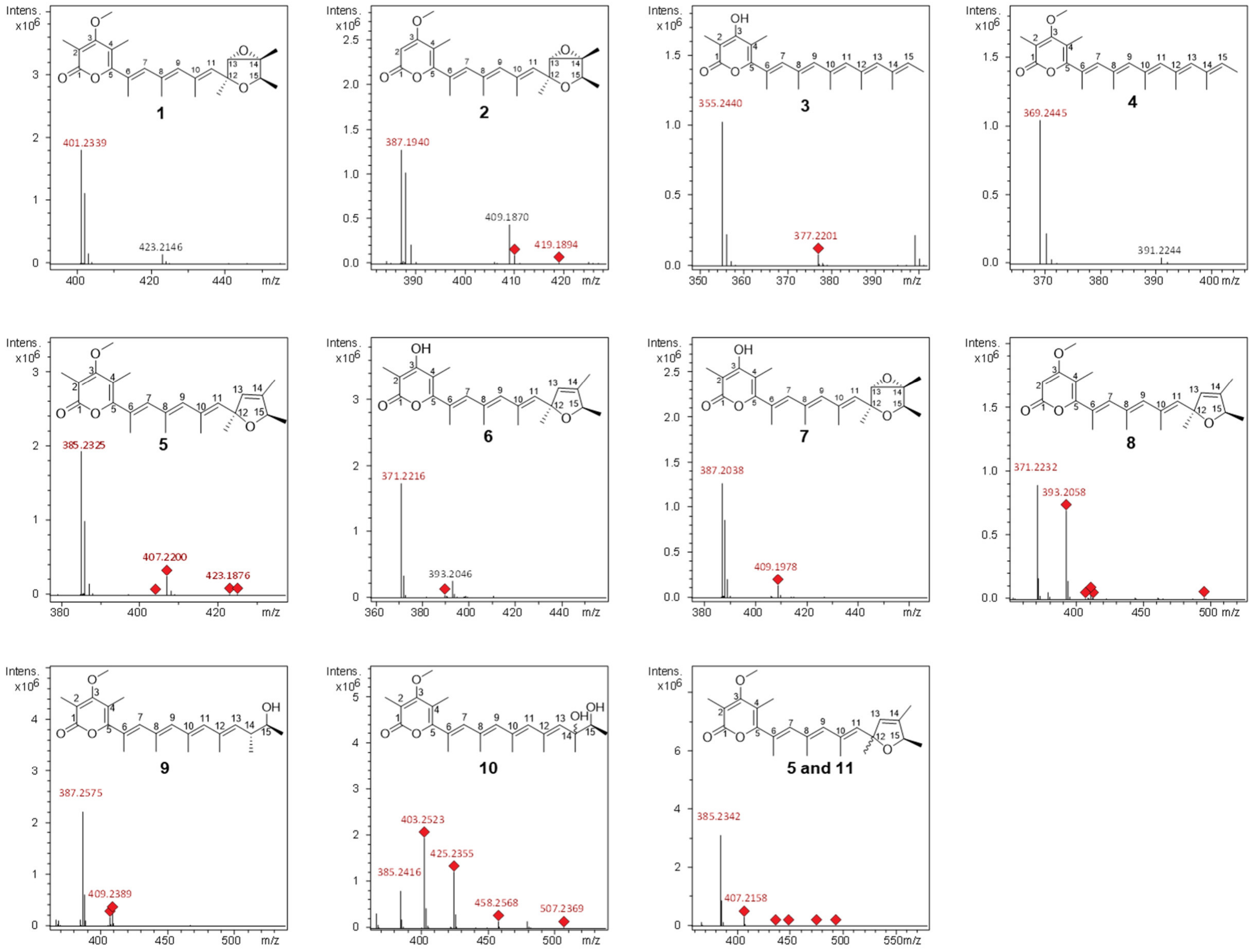

Figure S56. Mass spectra of compounds **1**–**11**.

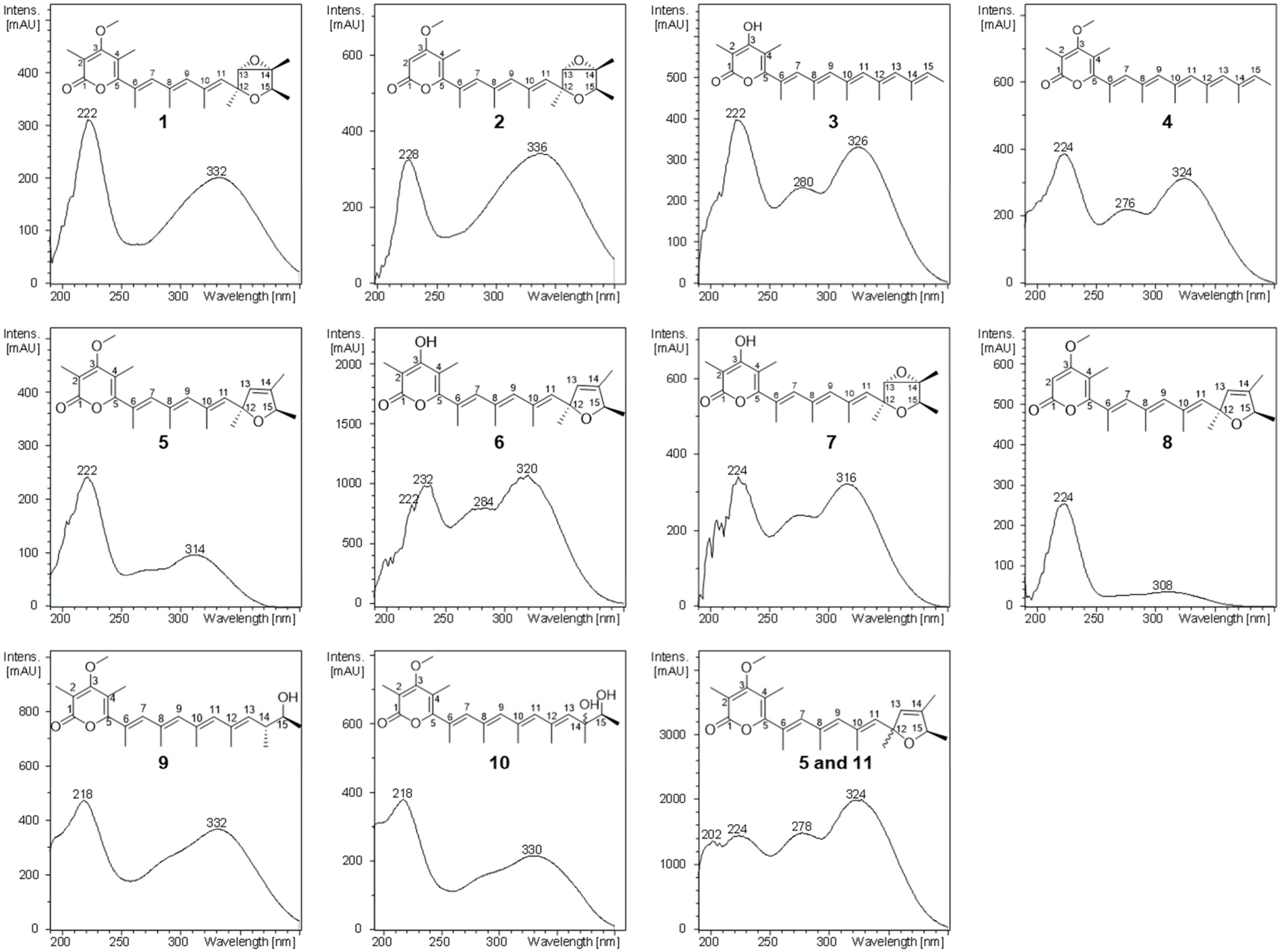

Figure S57. UV spectra of compounds **1**–**11**.

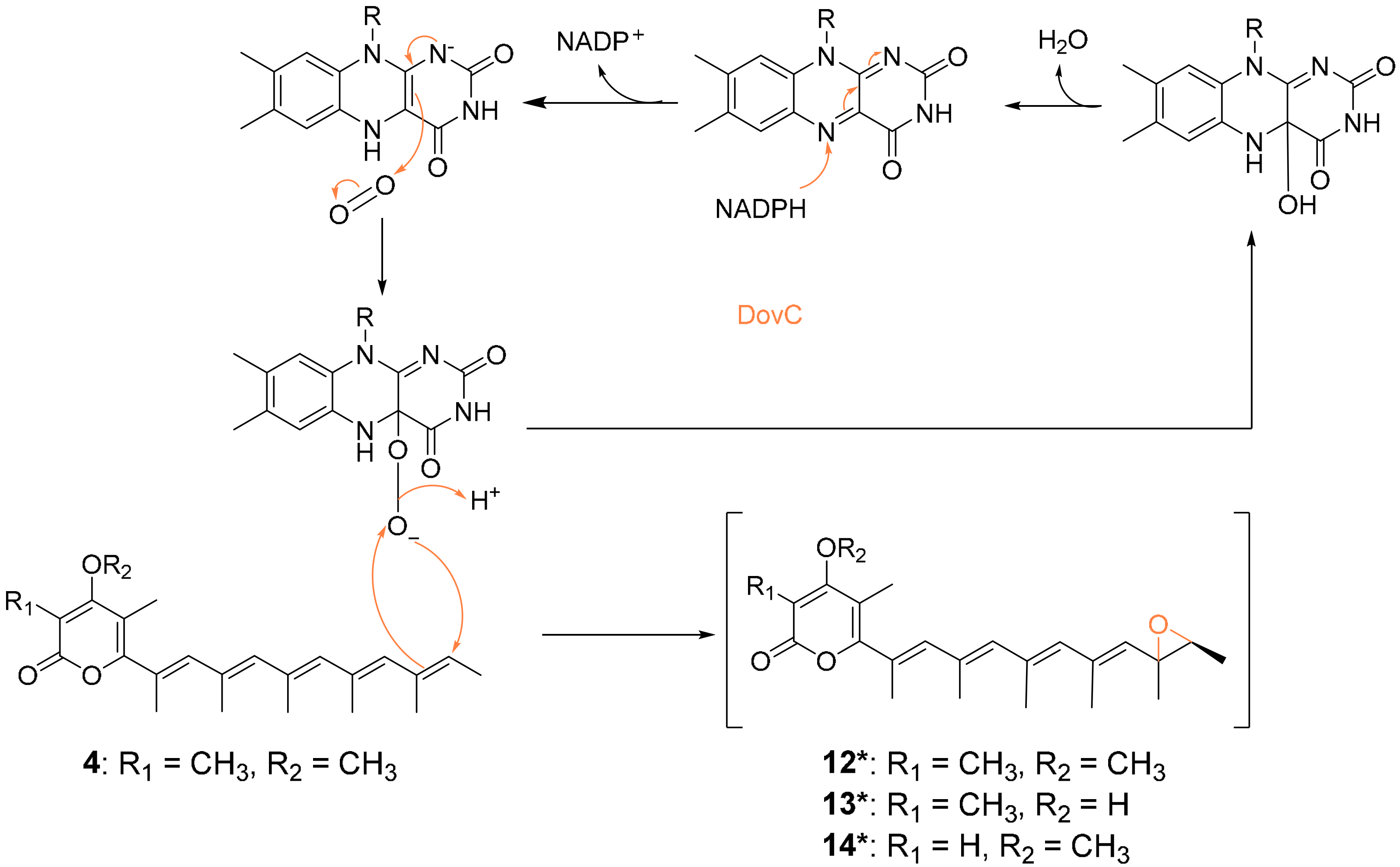

Figure S58. Proposed mechanism of the DovC epoxidation.

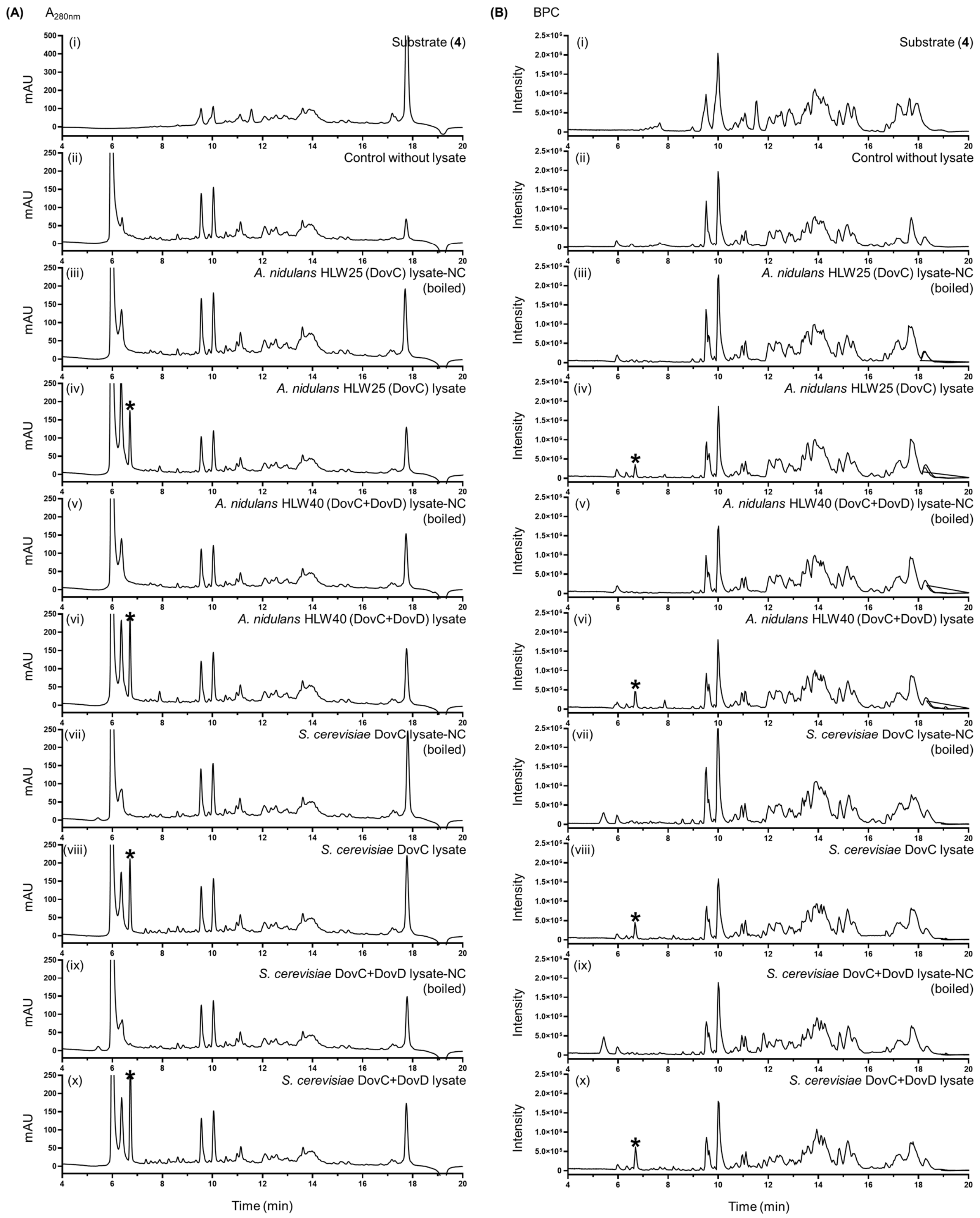

Figure S59. LC-MS analysis of *in vitro* assays with DovC- and DovD-containing lysates.

(A) Illustration of UV absorptions at 280 nm and (B) Detection of ions at m/z from 100 – 1500. The peak at 6.6 min, marked with an asterisk (*), has an $[M+H]^+$ of 377.145 ± 0.005 and corresponds to that of riboflavin. This peak was not detected in incubation mixtures with boiled lysates and is therefore likely a hydrolytic product of FAD in the assays by host enzymes. This phenomenon has also been observed in a previous study.[17]

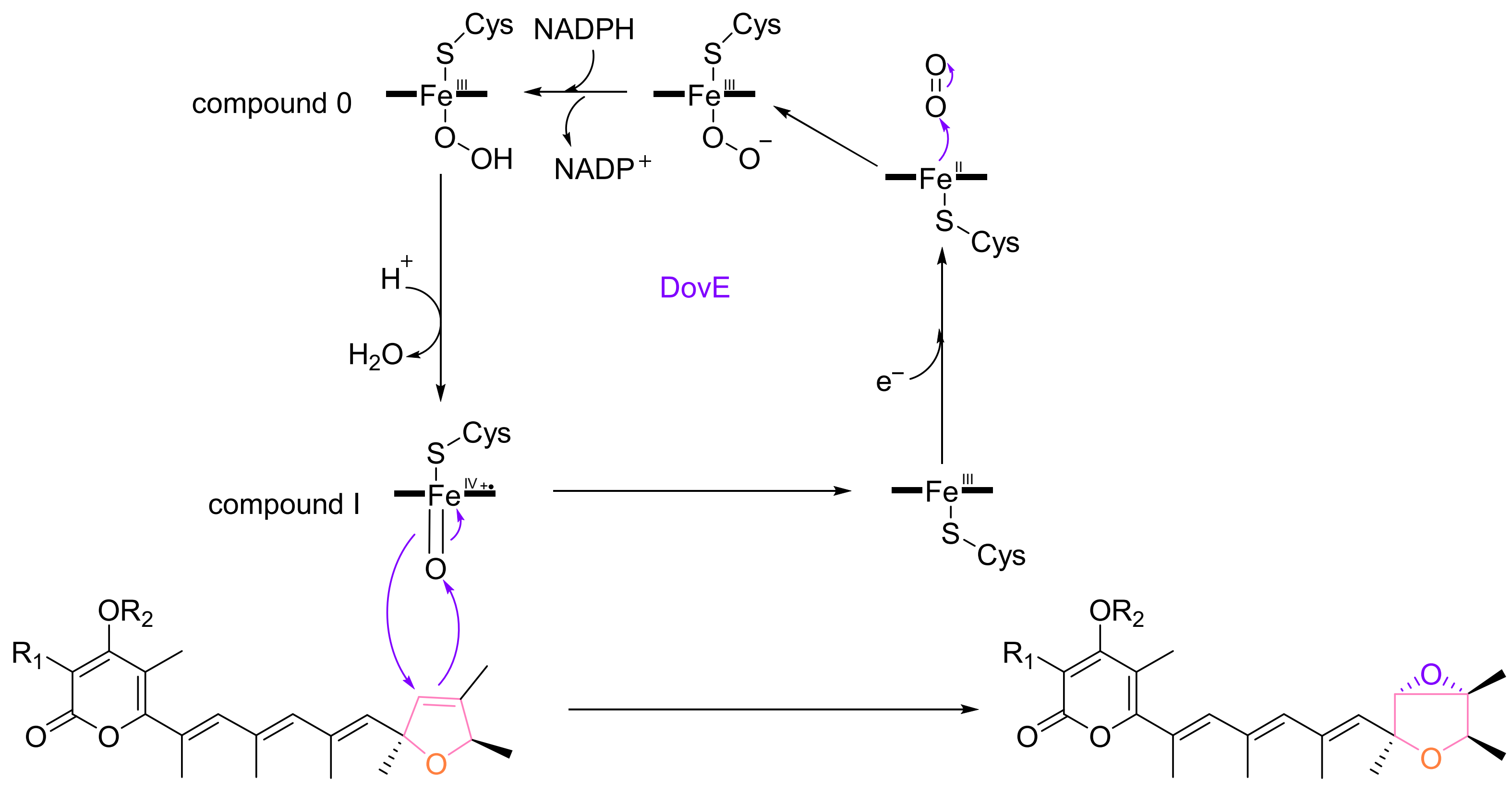

5: $R_1 = CH_3$, $R_2 = CH_3$
6: $R_1 = CH_3$, $R_2 = H$
8: $R_1 = H$, $R_2 = CH_3$

1: $R_1 = CH_3$, $R_2 = CH_3$
2: $R_1 = H$, $R_2 = CH_3$
7: $R_1 = CH_3$, $R_2 = H$

Figure S60. Proposed mechanism of the DovE epoxidation.

In the presence of DovABCE and absence of DovD

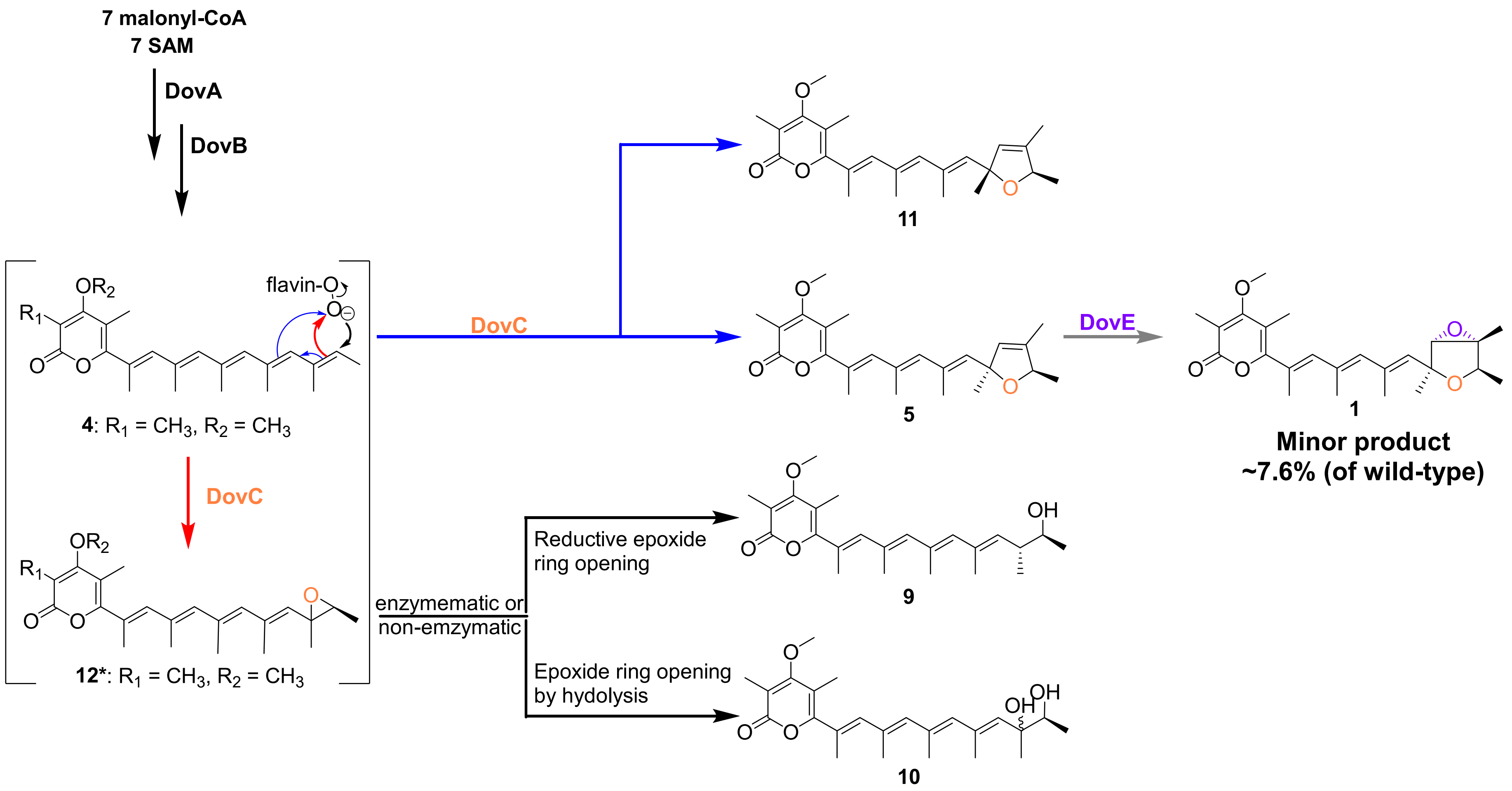

Figure S61. Proposed biosynthetic pathway of shunt products in the absence of DovD.

Supplementary References

1 M. R. Green and J. Sambrook, *Molecular cloning: a laboratory manual*, Cold Spring Harbor Laboratory Press, Cold Spring Harbor, New York, 2012, 4th.

2 K. R. Oldenburg, K. T. Vo, S. Michaelis and C. Paddon, *Nucleic Acids Res.*, 1997, **25**, 451.

3 A. P. Jacobus and J. Gross, *PLoS. One.*, 2015, **10**, e0119221.

4 R. S. Goswami, *Methods Mol. Biol.*, 2012, **835**, 255.

5 J. Fan, G. Liao, F. Kindinger, L. Ludwig-Radtke, W.-B. Yin and S.-M. Li, *J. Am. Chem. Soc.*, 2019, **141**, 4225.

6 Z.-H. Li, Y. Dai, J. Zhou, L. Yang and S.-M. Li, *Org. Lett.*, 2025, **27**, 2433.

7 Soo-Jin Choo, Hae-Ryong Park, In-Ja Ryoo, Jong-Pyung Kim, Bong-Sik Yun, Chang-Jin Kim, Kazuo Shin-ya, and Ick-Dong Yoo, *J. Antibiot.*, 2005, **58**, 210.

8 Yan Xu, Miao Zhang, Qing-Ai Liu, Lian-Dong Hu, Wan Li, Hua-Jie Zhu, Li Liu and Fei Cao, *Nat. Prod. Commun.*, 2018, **13**, 1329.

9 Y. Li, X. Li, X. Li, S. Yang, B. Wang and H. Li, *Int. J. Mol. Sci.*, 2022, **23**, 5567.

10 F. M. Ausubel, R. Brent, R. E. Kingston, D. D. Moore, J. G. Seidman, J. A. Smith and K. Struhl, *Current Protocols in Molecular Biology*, John Wiley and Sons Inc, New York, 1996.

11 Noel M O'Boyle, Tim Vandermeersch, Christopher J Flynn, Anita R Maguire, Geoffrey R Hutchison, *J. Cheminform.*, 2011, **3**, 8.

12 T. Bruhn, A. Schaumlöffel, Y. Hemberger and G. Bringmann, *Chirality*, 2013, **25**, 243.

13 C. Bond, Y. Tang and L. Li, *Fungal Genet. Biol.*, 2016, **89**, 52.

14 A. Grundmann, T. Kuznetsova, S. S. Afiyatullov and S.-M. Li, *ChemBioChem*, 2008, **9**, 2059.

15 Y.-M. Chiang, M. Ahuja, C. E. Oakley, R. Entwistle, A. Asokan, C. Zutz, C. C. C. Wang and B. R. Oakley, *Angew. Chem., Int. Ed.*, 2016, **55**, 1662.

16 F. Kindinger, J. Nies, A. Becker, T. Zhu and S.-M. Li, *ACS Chem. Biol.*, 2019, **14**, 1227.

17 Y. Dai, X.-L. Xie, H.-F. Dai and S.-M. Li, *Org. Lett.*, 2023, **25**, 4092.
